# Functional Analysis of Odorant-Degrading Enzymes Responding to the Repellent Activity of 2,3-Dimethyl-6-(1-Hydroxy)-Pyrazine Against *Plutella xylostella*

**DOI:** 10.3390/insects17101070

**Published:** 2026-10-16

**Authors:** You-Lu Zhou, Xian-Wen Wang, Bu-Guo Wang, Zi-Xian Wang, Qing Li, Hai-Qun Cao, Quan Gao

**Affiliations:** 1 Key Laboratory of Agri-Products Quality and Biosafety, Anhui Agricultural University, Ministry of Education, Hefei 230036, China; 2 Anhui Province Engineering Laboratory for Green Pesticide Development and Application, School of Plant Protection, Anhui Agricultural University, Hefei 230036, China

**Keywords:** *Plutella xylostella*, 2,3-dimethyl-6-(1-hydroxy)-pyrazine, repellent activity, odorant-degrading enzyme, glutathione *S*-transferases

## Abstract

*Plutella xylostella* is an important cruciferous crop pest with critical resistance to many chemical insecticides; 2,3-dimethyl-6-(1-hydroxy)-pyrazine has shown obvious repellent activity against *P. xylostella* with significant effects on the olfactory system. Here, we obtained 55 different expressions ODE (odorant-degrading enzyme) genes from the antennae of *P. xylostella* for 2,3-dimethyl-6-(1-hydroxy)-pyrazine treatment using RNA-seq. Three candidate *PxylODEs* were identified using RT-qPCR: *PxylCYP9f2*, *PxylGSTe1*, and *PxylGSTs1.* They each had log_2_-fold change values of 0.67, 2.39, and 2.08, respectively. *PxylGSTs1* had a significant expression level in the antennae of *P. xylostella* adults, where the content in males was 3.60-fold higher than in females. Moreover, the binding style between PxylODEs and pyrazine was mainly hydrophobic, and the binding energy was −5.82 kcal mol^−1^ of *PxylGSTs1*, determined using molecular dynamics and docking. Finally, PxylGSTS1 was calculated to have a higher binding affinity to pyrazine with a Ki value of 11.63 μmol L^−1^ according to a fluorescence competitive binding experiment, which was validated with mutations of the TYR7 side chain. These findings illustrate that PxylGSTS1 plays an important role in the response to 2,3-dimethyl-6-(1-hydroxy)-pyrazine, which can be used in the green control of *P. xylostella* and other Lepidopteran pests in the field.

## Simple Summary

Extensive resistance due to the widespread application of chemical insecticides is detrimental to the green control of *Plutella xylostella*. In our previous study, we found that 2,3-dimethyl-6-(1-hydroxy)-pyrazine has significant repellent activity against *P. xylostella*, influencing the olfactory system. This study illustrates that *PxylGSTs1* is a primary target responding to the repellent activity of 2,3-dimethyl-6-(1-hydroxy)-pyrazine, which was highly expressed in male antennae. Moreover, binding between PxylGSTS1 and pyrazine is mainly hydrophobic, and the TYR7 side chain is validated via site-directed mutation. These data provide novel insights into the green control of Lepidoptera insects, especially 2,3-dimethyl-6-(1-hydroxy)-pyrazine, which can be used as a lead compound.

## 1. Introduction

Diamondback moth (DBM, *Plutella xylostella*) is one of the most important and destructive cruciferous crop pests. It causes serious product and economic losses worldwide. Currently, chemical insecticides are the main method of controlling *P. xylostella* in the field, which has led to clear negative effects on green agricultural development, including pesticide residues, environmental pollution, and extensive resistance [1,2]. It has been determined that *P. xylostella* shows strong resistance to multiple insecticides with contact or stomach toxicity, including organophosphorus, carbamate, organochlorine, and pyrethroid insecticides [3,4,5]. The resistance ratio of *P. xylostella* to chlorantraniliprole showed significant differences between various regions in China; the value in 2021 was 1428-fold and 6642-fold in Jiangsu and Guangdong, respectively [6]. Simultaneously, the DBM in Gansu has been found to have a higher level of resistance to lambda-cyhalothrin, with the control effect on the field colony reaching only 29.03% [7]. It was found that the DBM in Yunnan showed high-level resistance to abamectin and beta-cypermethrin, while middle-level resistance was maintained for spinosad, indoxacarb, and chlorfenapyr [8]. In addition, the DBM, multi-treated with chlorantraniliprole, showed cross-resistance to matrine, chlorantraniliprole, and rotenone. However, the change in ratio was not significant [9]. Subsequently, the DBM showed high-level resistance to hexaflumuron and lufenuron, possibly due to the levels and habits of the insecticides utilized [10]. Hence, a novel compound with repellent or antifeedant activity has been considered a research hotspot for the green control of *P. xylostella*.

Recently, natural volatile components have shown significant potential as effective ingredients to control agricultural pests. For example, when the treatment dosage of volatile components (myrcene: γ-terpinene: linalool: camphor: terpinen-4-ol = 1:5:3:4:3, mass ratio) in *Pelargonium hortorum* was 10 μg/μL, obvious repellent activity to *P. xylostella* was observed, and the oviposition repellent activity was sustained for 26 days [11]. The extract from dried roots of *Angelica pubescens* demonstrated several types of repellent activity to female and male *P. xylostella* adults; caryophyllene oxide was identified as the main active ingredient with repellent rates of 74.00% and 60.00%, respectively, when the treatment concentration was 0.63 mg/mL [12]. Moreover, it was shown that Neem oil had significant antifeedant activity against *P. xylostella*, which can inhibit the eclosion behavior of adults [13]. The three components D-limonene, 2-phenylethyl alcohol, and methyl 2-methoxybenzoate (3:2:1) are effective attractants, which hold great potential for monitoring and managing *P. xylostella* populations [14]. Age and sex had interaction effects on EAG (Electroantennography) responses of *P. xylostella* to trans-2-hexenal, trans-2-hexenol, heptanal, and cis-3-hexen-1-ol; females had significantly stronger responses than males, and values were enhanced with increasing concentrations [15]. In our previous study, it was determined that pyrazines showed significant repellent activity to *P. xylostella*, especially 2,3-dimethyl-6-(1-hydroxy)-pyrazine, which had clear influences on the olfactory system [16,17,18].

The antenna is an important communication organ for perceiving external volatile substances in adult Lepidoptera, where odorant receptors (ORs) identify the combination of substances and odorant-binding proteins (OBPs) or the compounds transferred to ORs by OBPs [19,20]. Adults showed different responses based on the activity of the volatile compounds, which are affected by odorant-degrading enzymes (ODEs) after a period [21]. ODEs are essential in preserving the olfactory acuity of insects by removing redundant odorants in the antennae, which usually contain carboxylesterases (CXEs), cytochrome P450 (CYPs), glutathione S-transferases (GSTs), and UDP-glycosyltransferases (UGTs) [22]. It has been reported that insect CXEs can degrade many plant volatile components, pheromones, hormones, and pesticides [23,24]. CYPs are another important ODE: their primary function is to detoxify chemical insecticides within the body. Several CYPs can remove terpenoids from the antennae and detoxify host terpenoids to overcome plant defenses [25,26]. Moreover, some GSTs and UGTs were determined to be linked to odorant degradation; GST-msolf1 restricted to pheromone sensilla could inactivate the sex pheromone blend in *Manduca sexta* [27]; and the antenna aldehyde oxidase gene from *P. xylostella*, PxylAOX3, oxidized both sex pheromone compounds and plant-derived aldehydes [28]. GmolCXE1 and GmolCXE5 in *Grapholita molesta* were responsible for hydrolyzing the acetate sex pheromone (Z/E)-8-dodecenyl, while GmolCXE14 and GmolCXE21 were involved in metabolizing the ester host plant volatiles ethyl butanoate and ethyl hexanoate [29]. Additionally, DponCYP345E2 and DponCYP6DE1 of *Dendroctonus ponderosae* can catalyze α-pinene; CYP3 P450 genes of *D. armandi* can degrade pinene, 3-carene, and limonene; all of these compounds show significant repellent or fumigant activity [30,31]. Hence, it can be concluded that ODEs play an important role in the degradation of insecticidal components.

ODEs showed high specificity to different compounds belonging to well-studied detoxification enzyme classes [32]. Cytochrome P450s, usually associated with xenobiotic degradation, could protect insect olfactory receptor neurons against volatile natural toxins or insecticides. This has been implicated more directly in pheromone metabolism [33]. It has been reported that *CYP6g1* in *D. melanogaster* was associated with dichloro-diphenyl-trichloroethane and neonicotinoid resistance, one of the most highly expressed P450s in the antennae [34,35]. Delta (GSTD) and epsilon (GSTE) GSTs are the only GST groups implicated in insecticide resistance; GSTE4 also shows a higher level of antennal expression than any of the other GSTs [36]. Some CXEs are male antennae-specific or antennae-enriched in *Antheraea polyphemus*, *Popillia japonica*, *Spodoptera littoralis*, and *Drosophila melanogaster*, which have been functionally characterized and shown to be involved in odorant inactivation [37,38,39,40]. SexiCXE10 in *S. exigua* is preferred to hydrolyze plant volatiles [41]. Furthermore, some *α*-esterases were known to be involved in organophosphate resistance, where the expression level was enhanced to accelerate organophosphate hydrolysis or mutations were produced to reduce sensitivity [42,43]. In *S. exigua*, GST activity was regulated by exposure to plant extracts, heavy metals, and insecticides; the delta GSTs, *SeGSTd1* and *SeGSTd3*, have been implicated in resistance to chlorpyrifos and cypermethrin [44]. Therefore, an in-depth study on PxylODEs is an important strategy for the green control of *P. xylostella*.

In the previous study, we performed a comparative transcriptomic analysis of *P. xylostella* antennae between blank controls and those treated with 2,3-dimethyl-6-(1-hydroxy)-pyrazine, which showed significant repellent activity against adults, with clear negative effects on PxylOR31 and PxylPBP2 [16,17,18]. Based on *P. xylostella* genomic data saved in the NCBI database (National Center for Biotechnology Information) and previous RNA-seq studies on the antenna, 55 ODE genes which mainly belong to CYPs and GSTs were obtained and identified using RT-qPCR. Subsequently, in order to determine the main ODE responding to 2,3-dimethyl-6-(1-hydroxy)-pyrazine, we assayed the binding efficacy and sites of PxylODEs to pyrazine and performed molecular docking after heterologous expression. Moreover, a fluorescence competitive binding experiment was performed to illustrate the ligand-binding properties; the characteristics and function of the main ODE were validated using site-directed mutation. These results represent the analysis of PxylODEs responding to pyrazine or other volatile components with repellent activity, which is important for the utilization of these compounds as green pesticide ingredients and the green control of *P. xylostella* and other Lepidopteran pests.

## 2. Materials and Methods

### 2.1. Biological Samples

*P. xylostella* were continuously reared at the School of Plant Protection, Anhui Agricultural University, and assayed using standard procedures. Temperature was set at 27 °C, and relative humidity was 80% with a 16 h:8 h (light:darkness) photoperiod. Adults were used as test insects in this study.

### 2.2. Chemicals

The compound 2,3-dimethyl-6-(1-hydroxy)-pyrazine with a purity of >98% was obtained according to the established synthesis method in our previous study [18]. NMR (nuclear magnetic resonance spectroscopy) and UPLC-Qtof MS (ultra-performance liquid chromatography–time of flight mass spectrometry) were used to validate the structure and molecular mass of 2,3-dimethyl-6-(1-hydroxy)-pyrazine.

### 2.3. Selection of ODEs Responded to Repellent Activity of 2,3-Dimethyl-6-(1-Hydroxy)-Pyrazine

Thirty random *P. xylostella* adults were treated with 0.001 mg of 2,3-dimethyl-6-(1-hydroxy)-pyrazine as an individual treatment; three replicates and a blank control treatment were set. Next, *P. xylostella* antennae were collected in RNase-free Eppendorf tubes and total RNA was extracted with TRIzol reagent (Invitrogen, Carlsbad, CA, USA) according to the manufacturer’s protocol. RNA quality was evaluated using agarose gel electrophoresis with 1% gels and nucleic acid spectrometry, and RNA integrity number (RIN) was determined by BGI (Shenzhen, China) using an Illumina HiSeq 4000 platform (Illumina, San Diego, CA, USA). Clean reads were obtained by removing low-quality reads, reads contaminated with adapters, and reads with a high content of unknown nucleobases. Hierarchical indexing for spliced alignment of transcripts (HISAT2) was used to compare sequences with the reference genome (GCA_000330985.1) with a ratio of clean reads of more than 47% [45]. The randomness, coverage, and sequencing saturation of transcripts indicated that data can be used for further analysis. De novo assembly of the trimmed reads was performed with the Trinity software package. High-quality clean reads were aligned with reference gene sequences using Bowtie 2 software with default parameters by BGI [46]. The FPKM (Fragments Per Kilobase Million) method was used to calculate and normalize the expression level of genes, and DEGs were analyzed by the DEGs Package (DEGseq) [47]. GO (Gene Ontology) and KEGG (Kyoto Encyclopedia of Genes and Genomes) enrichment analyses were used to assign DEGs into functional categories based on the corresponding databases using the phyper function in R. The *p*-values were calculated and adjusted using the false discovery rate (FDR), and results with a Q value ≤ 0.05 were considered significant [16,17,18]. The obtained RNA-sequence data has been submitted to the NCBI database (PRJNA987853). Based on the RNA-seq data, fifty-five ODE genes were identified via an NCBI database blast.

Subsequently, to screen candidate target ODEs responding to the repellent activity of 2,3-dimethyl-6-(1-hydroxy)-pyrazine, validation was performed using RT-qPCR. *RPS3* was selected as a housekeeping gene; primers were designed and synthesized using Beacon Designer 8 software (Premier Biosoft International, Palo Alto, CA, USA), avoiding the primer interval for expressed RNAs (Table S2). Simultaneously, gradient dilution templates were used to evaluate the amplification efficiency of candidate and housekeeping genes; three replicates of each gene were processed.

Then, the product of first-strand cDNA was diluted 5-fold and used as the template for RT-qPCR. The reaction was assayed using the ChamQ universal SYBR qPCR master mix kit (Vazyme, Nanjing, China) with thermocycling conditions as follows: an initial denaturation step at 95 °C for 30 s, 40 cycles of denaturation at 95 °C for 5 s, and annealing at 60 °C for 30 s, with denaturation at 95 °C for 15 s, followed by 60 °C for 1 min, 95 °C for 15 s, and 60 °C for 15 s. The reaction system is listed in Table S4. The relative expression level of selected genes was calculated via the 2^−ΔΔCt^ method compared with the blank control treatment, which was used to assess the quantitative analysis of related genes.

### 2.4. Relative Expression Levels of the Three ODEs in P. xylostella Adults

For in-depth clarity on the characterization of these ODEs, the relative expression levels of *PxylCYP9f2*, *PxylGSTe1*, and *PxylGSTs1* in various single *P. xylostella* adult body parts (antenna, head, thorax, abdomen, leg, and wing) were determined without any treatments using RT-qPCR. The primers used in this experiment were the same as those shown in Table S5; the reaction system procedure is listed in Table S3.

Sequence analysis and phylogenetic tree creation were performed using DNAMAN (Lynnon Biosoft, San Ramon, CA, USA), SignalP 5.0 [48], and PhyloSuite [49]. The ORF sequences of *PxylGSTs* and five representative insects from the order Lepidoptera (Table S6, File S1), including *Bombyx mori*, *Cnaphalocrocis medinalis*, *Spodoptera litura*, *Eriocrania semipurpurella*, and *Spodoptera littoralis,* were selected and aligned using MAFFT (multiple alignment using fast Fourier transform). The sequences used to analyze *PxylCYPs* were obtained from *P. xylostella* and five representative insects (Table S7, File S2): *S. littoralis*, *B. mori*, *Helicoverpa armigera*, *S. frugiperda*, and *Chilo suppressalis*. The motifs were assessed using MEME (multiple em for motif elicitation); the online website (https://meme-suite.org/meme/index.html accessed on 16 May 2025) was used to input TBtools software (TBtools.v1.09854) to obtain visualization files [50].

### 2.5. Cloning of Candidate ODE Genes

Antenna RNA was extracted and cDNA was synthesized according to the above methods; one hundred random *P. xylostella* adults were used. Primer 5.0 software was used to design the specific primers for the candidate ODEs, as listed in Table S4. Subsequently, this amplification was assayed according to the protocol of the Prime STAR^®^ HS (Premix) kit (Takara, Kusatsu, Japan) with cDNA as the template. The reaction system is listed in Table S5; the procedure followed an initial denaturation step at 98 °C for 3 min; 34 cycles of denaturation at 98 °C for 10 s; annealing at 55 °C for 10 s; and extension at 72 °C for 45 s (*PxylGSTe1, PxylGSTs1*) or 75 s (*PxylCYP9f2*), followed by 72 °C for 5 min, and the product was stored at 4 °C.

The 1% agarose gel electrophoresis was used to detect the candidate target fragment after the PCR amplification procedure was completed (Figure S1). The right fragment was cut and placed in a new 1.5 mL EP (Eppendorf) tube. Then, the sample was purified using the SanPrep column DNA gel extraction kit (Sangon Bio, Shanghai, China) with standard operations. The obtained DNA solution was stored at −20 °C and was used as a template to convert the PCR product using the Hieff clone^®^ zero TOPO-blunt simple cloning kit (Yeasen, Shanghai, China). The reaction system is listed in Table S8, showing reactions for 5 min at the specified temperature. Subsequently, the *DH5α* competent cell (100 μL) was placed on ice for 5 min, and then mixed with 10 μL of the above-mentioned product. The mixture was placed in a water bath (42 °C) for 45 s after an ice bath for 30 min, and then 500 μL of LB medium with antibiotics was added. Next, treatment was conducted at 200 rpm at 37 °C for 60 min; 100 μL of solution was distributed on the surface of the solid LB (Luria–Bertani) medium plate with Amp^+^, which was inverted and cultivated overnight at 37 °C.

A single colony was added to the 1.5 mL EP tube with 10 μL of nuclease-free water; positive clone validation was performed using the MonAmp^™^ 2 × Trio taq mix kit (Monad, Shanghai, China) with a corresponding reaction system (Table S9). The reaction procedure was determined as an initial denaturation step at 94 °C for 5 min; 34 cycles of denaturation at 94 °C for 30 s; annealing at 55 °C for 30 s; and extension at 72 °C for 30 s, followed by 72 °C for 5 min. Finally, it was stored at 12 °C. Subsequently, the product was placed into the LB medium containing Amp^+^ after detection and validation. It was cultivated at 37 °C and 200 rpm, and then the solution was sequenced according to the standard method. Finally, the candidate target fragment plasmid was extracted using the FastPure^®^ plasmid mini kit (Vazyme, Nanjing, China) with the related protocol; the obtained DNA solution was stored at −20 °C after the concentration was determined.

### 2.6. Construction of Candidate ODE Expression Vector

Based on the prediction results of a signal peptide, the CDS (coding sequence) was obtained after the signal peptide nucleotide sequences were removed from the ORF region. The homologous arm primers were designed using Primer Premier 5.0 software and are shown in Table S10; enzyme cleavage sites were introduced and marked with an underline. Amplification of candidate target ODE fragments and recycling of the PCR product were assayed according to the above methods; finally, the DNA solution was stored at ™20 °C.

The plasmid of the pET-30 (+) vector was used to assay the double enzyme digestion reaction with restriction endonucleases, *NcoI*, *NdeI,* and *XhoI*, which was conducted using QuickCut™*NcoI*, QuickCut™*NdeI*, and QuickCut™*XhoI* kits (TaKaRa, Dalian, China). The reaction system is listed in Table S11; the solution was mixed and centrifuged before the PCR. After the reaction was completed, a linearized plasmid DNA solution was obtained and stored at −20 °C. Then, the MonClone^™^ single-assembly cloning mix kit was used to assay the homologous recombination reaction, and the linearized plasmid DNA solution was mixed with candidate target DNA solution of homologous arm fragments in an ice bath. The reaction system is listed in Table S12, and the sample was stored at −20 °C until the recombination product was converted.

### 2.7. Extraction to Recombination Plasmid

The *DH5ɑ* competent cell was placed on ice for 5 min; 10 μL of the above recombination product was added to the system and was then placed on ice for 30 min. Subsequently, this system was immersed in water (42 °C) for 45 s and mixed with 500 μL of LB medium without any antibiotics after being placed on ice for 5 min. Then, 100 μL of the solution was distributed on the surface of a solid LB medium plate with Kan^+^, which was inverted and cultivated overnight at 37 °C.

A single colony was obtained and placed into a 1.5 mL EP tube to detect positive clones of the recombination product; the reaction system is listed in Table S13 according to the standard PCR procedure. In total, 1% agarose gel electrophoresis was used to detect the PCR amplification product; the right colony was cultivated in LB medium containing Kan^+^ at 37 °C and 200 rpm, validated by the sequencing procedure. Then, the recombination plasmid was extracted according to the above method, and the DNA solution of the recombination plasmid was quantified and stored at −20 °C.

### 2.8. Prokaryotic Recombination Expression and Purification of Candidate ODEs

Firstly, 10 ng of recombination plasmid was transferred to the *BL21* competent cells via the above-mentioned method; positive clones were obtained after the validation step was completed. Then, the right bacterial solution was placed in 5 mL of LB medium containing Kan^+^ and cultivated at 37 °C and 200 rpm overnight. The bacterial solution was transferred into a new LB medium containing Kan^+^ at a concentration of 50 μg/mL for the small induction test at 37 °C and 200 rpm; it was performed until the value of OD_600_ ranged from 0.5 to 0.6. Subsequently, IPTG (Isopropyl-beta-D-thiogalactopyranoside, final concentration 1 mM) was added to the solution to induce the expression of candidate target proteins, and the culture was cultivated for 6 h at 37 °C and 200 rpm; the blank control was set up simultaneously. Next, the precipitate was treated with an ultrasonic cell crusher after the supernatant was removed. The buffer solution was mixed with several proteins and placed in boiling water for 10 min; it was analyzed using SDS-PAGE (sodium dodecyl sulfate–polyacrylamide gel electrophoresis). Subsequently, the large-scale induction of the candidate proteins was assayed via a similar method at a larger scale (Figure S2).

Candidate proteins were purified using a HyPur T Ni-NTA 6FF (His-tag, Sangon Bio, Shanghai, China) prepacked gravity column kit with standard procedures. The high-purity protein solutions were selected to be assayed in the next study. Dialysis refolding treatment is necessary for highly expressed candidate proteins; the gradient concentrations of urea were set at 6 mol/L, 4 mol/L, 3 mol/L, 2 mol/L, 1 mol/L, and 0, respectively. The dialysis was performed at 4 °C for 2 h, and the formulation was composed of 5% glycerol, 1% L-arginine, 2% glycine, and different concentrations of urea, dissolved in PBS (phosphate-buffered saline) buffer solution. The candidate proteins were purified and placed into new 1.5 mL EP tubes, respectively. Finally, the concentration of proteins was detected using the BCA (Bicinchoninic acid) protein quantification kit (Vazyme, Nanjing, China) with a microplate reader.

### 2.9. Ligand Binding Characteristics of Candidate ODEs

Candidate proteins were diluted to 2 μmol/L using PBS buffer solution with a pH value of 7.4; the N-phenyl-1-naphthylamine (1-NPN) fluorescent probe and 2,3-dimethyl-6-(1-hydroxy)-pyrazine were diluted to a final concentration of 1 mmol/L via HPLC-grade methanol, respectively. Evaluation of the ligand binding characteristics of candidate ODEs in *P. xylostella* was determined using a fluorescence spectrophotometer (F-7000, Hitachi, Tokyo, Japan). Firstly, the dissociation constant K1-NPN between candidate ODEs and 1-NPN was determined; the emission spectra were recorded between 350 and 500 nm with an excitation wavelength of 337 nm. The protein solution and 1-NPN aliquot were added to a 10 mm quartz cuvette, and the maximum fluorescence intensities were recorded when the reaction ceased and the intensity was stable. The values were titrated with aliquots to final concentrations of 2–20 μmol/L, with an increasing rate of 2 μmol/L; each treatment was repeated 3 times.

Next, the binding affinity of ODEs and 2,3-dimethyl-6-(1-hydroxy)-pyrazine was measured and recorded until the intensity of the mixture decreased to 50%, and the maximum concentration was 32 μmol/L with an increasing rate of 4 μmol/L. The dissociation constants (K*i*) of competitors to ODEs were calculated from the corresponding IC_50_ values using the equation K*i* = [IC_50_]/(1 + [1-NPN]/*K*_1-NPN_), where [IC_50_] is the concentration of the competitor with half the initial fluorescence intensity; [1-NPN] is the free concentration of 1-NPN; and K_1-NPN_ is the dissociation constant of the protein and 1-NPN complex.

### 2.10. Molecular Dynamics and Docking Analysis for Candidate ODEs

To determine the link stability between ODEs and the ligand, 100 ns of molecular dynamics was assessed. Protein and ligand typing were performed using the charmm36 field and the match algorithm, respectively. Subsequently, the TIP3P H_2_O molecule model was used to solve the complex system; the distance to the boundary was set to 7 Å. PME (particle mesh Ewald) was used to calculate the long-range interaction effect, and sodium chloride (0.145 mol/L) was used to neutralize the charge of the system. Standard dynamics cascading was used to initialize the dynamic simulation after the solvation of the system. Firstly, energy minimization of the protein skeleton was performed using the 1000-step steepest descent method and the 5000-step conjugate gradient method under heavy atom constraints to remove unfavorable spatial hindrance in the system. Then, a heating phase and an equilibrium phase of 200 ps were performed using a time step of 2 fs under the harmonic constraint of the protein skeleton. All simulations were performed under periodic boundary conditions of the NPT (non-parametric transformer) ensemble (constant pressure and temperature) at 300 K; the distance cutoff value for counting non-bonded interaction pairs was 14 Å, and the local point was 12 Å. In addition, SHAKE (secure hash algorithm with keccak) was used to constrain all covalent bonds involving hydrogen. After the initial kinetic temperature and energy were ensured to reach equilibrium, 100 ns of the unconstrained sampling stage was performed with NAMD (nanoscale molecular dynamics, 2.13-GPU version, University of Illinois, Champaign); temperature and pressure control were implemented using Langzhiwan dynamics and pistons, respectively.

Trajectory analysis was performed for composite systems: the RMSD (root mean square deviation) and interactions such as hydrogen bonding and hydrophobic contact at the interface between each frame and the first frame were surveyed. This is an effective tool for evaluating the overall trajectory stability of a system, corresponding to the classical Euclidean distance between two structures. It is an effective tool for evaluating the overall trajectory stability of a system. Firstly, the 3D structure of 2,3-dimethyl-6-(1-hydroxy)-pyrazine was drawn using Chemdraw 20.0 software (Perkin Elmer, Waltham, MA, USA) [16]. The homology and 3D structure of PxylODEs were determined according to the results of multiple sequence alignment and phylogenetic tree analysis; the modeling prediction of PxylODEs was performed on the website https://swissmodel.expasy.org/ accessed on 20 June 2025, a homology modeling server. Then, the protein structures in the PDB (protein data bank) database with amino acid sequence similarity greater than 30% were retrieved, and the protein structure PDB format files were downloaded. The structures of 2,3-dimethyl-6-(1-hydroxy)-pyrazine and candidate proteins were imported into BIOVIA Discovery Studio, and their structures were optimized using the energy minimization algorithm in the CHARMM (chemistry at Harvard macromolecular mechanics) function. Subsequently, prepared protein and ligand functions were used to complete the pre-treatment steps: hydrogen atom addition and valence state repair. The CDOCKER module was used to validate molecular docking based on molecular dynamics analysis, and visualization and calculation of the binding energy of the molecular docking structure were performed via PyMOL 3.1.8 (python molecular viewer) software.

### 2.11. Analysis of the Binding Mechanism Between PxylGSTS1 and Ligand

The sequence of *PxylGSTs1* was inserted into SnapGene [51]; the amino acids at the four possible key binding sites of PxylGSTS1 and 2,3-dimethyl-6-(1-hydroxy)-pyrazine were replaced with alanine based on the results of molecular docking. Secondly, the new amino acid sequences were inserted using the Swiss Model for modeling prediction; the binding energy of these four proteins was calculated and used as the basis to proceed with the next analysis. CE design software (V1.04, Vazyme, Nanjing, China) was chosen to obtain the single-base (or continuous multi-base) site for directed mutagenesis primers, as shown in Table S14. PCR amplification was performed using the Mut Express^®^ II Fast Mutagenesis Kit V2 (Vazyme, Nanjing, China) with the amplification reaction system of the target plasmid (Table S15).

The reaction procedure was determined as an initial denaturation step at 95 °C for 30 s, 30 cycles of denaturation at 95 °C for 15 s, annealing at 70 °C for 15 s, and extension at 72 °C for 3 min, followed by 72 °C for 5 min, and was finally stored at 4 °C. Subsequently, the product was validated using 1% agarose gel electrophoresis, and the correct plasmid was used to assay the next Dpn I digestion procedure with the reaction system at 37 °C for 2 h (Table S16). Then, the recombination reaction was performed after the optimal amount of Dpn I digestion product was selected according to the number of fragment base pairs, and the reaction system was prepared on ice (Table S17). The system was mixed well without shaking, and the reaction solution was collected at the bottom of the tube after brief centrifugation. The reaction was completed at 37 °C for 30 min, and the product was immediately cooled on ice and stored at −20 °C. Moreover, the recombination product was identified, and the plasmid was extracted via the above-mentioned method. The mutant recombination protein was obtained after inducing expression. Subsequently, the PxylGSTS1^Y7A^ protein was purified and refolded by dialysis via the established method in this study; the concentration was determined and used in the next test. Finally, the fluorescence competitive binding experiment was assayed to validate the binding ability between PxylGSTS1^Y7A^ and 2,3-dimethyl-6-(1-hydroxy)-pyrazine using the above-mentioned method, which also compared PxylGSTS1.

## 3. Results

### 3.1. Bioinformatics Analysis of Candidate PxylODEs’ Response to 2,3-Dimethyl-6-(1-Hydroxy)-Pyrazine

**Figure 1:**
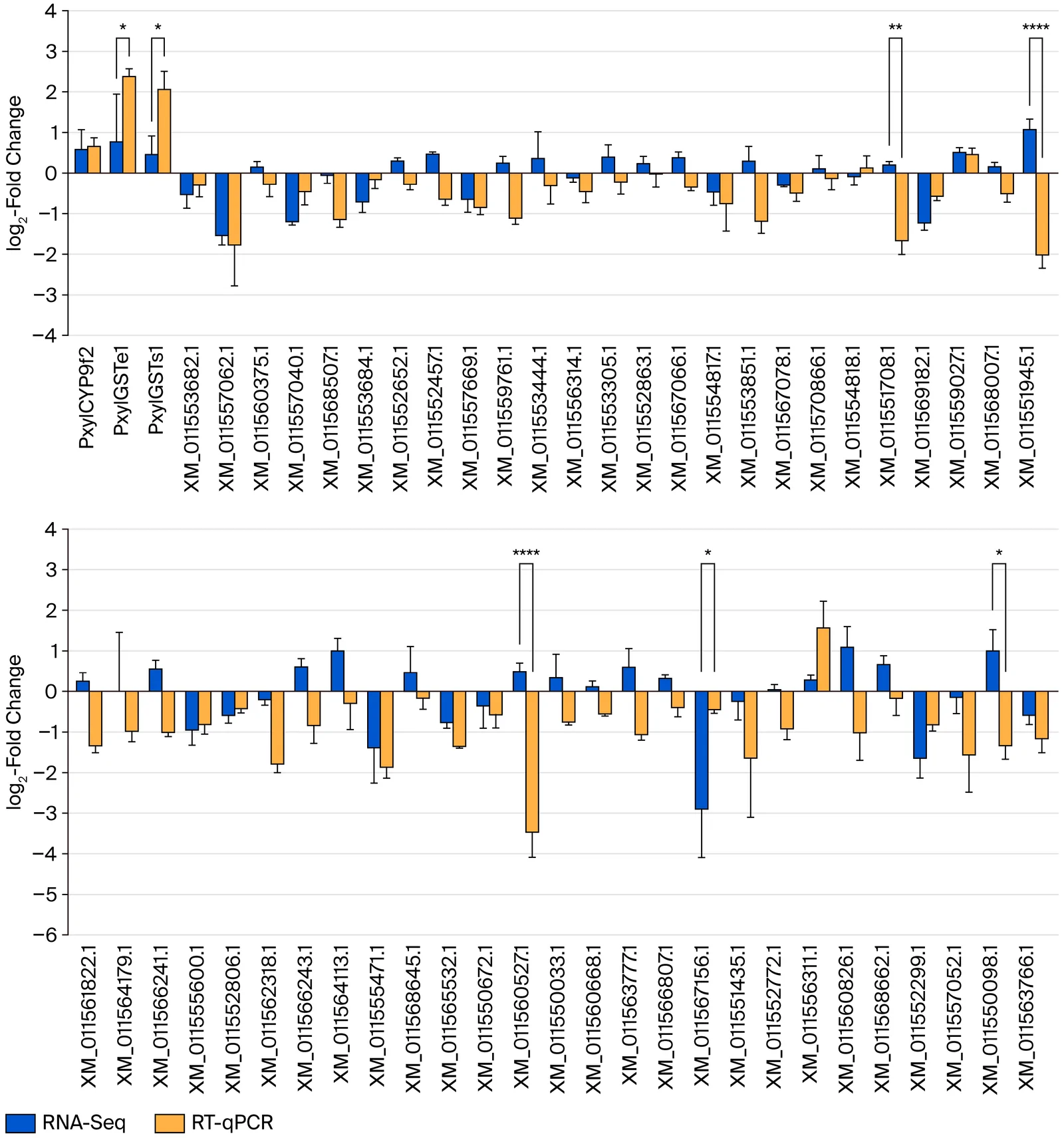
Expression of 55 ODEs obtained via RNA-Seq data in *P. xylostella* treated with 2,3-dimethyl-6-(1-hydroxy)-pyrazine and compared with RT-qPCR. Gene expression (mean ± standard error) was quantified as the relative fold change using the 2^−ΔΔCT^ method, and *RPS3* was used as the housekeeping gene. The bars represented the mean standard error (n = 3), and the asterisks indicated significant differences between RNA-Seq and RT-q-PCR data. *, ** and **** denoted significant difference at *p* < 0.05, 0.01 and 0.0001, respectively.

Based on the RNA-seq results of *P. xylostella* antennae treated with 2,3-dimethyl-6-(1-hydroxy)-pyrazine [16,17,18], a total of 55 differentially expressed ODE genes were selected to identify the response of candidate ODEs to the repellent activity of 2,3-dimethyl-6-(1-hydroxy)-pyrazine against *P. xylostella* adults via RT-qPCR. As shown in Figure 1, most changes in ODEs showed similar trends between RNA-seq and RT-qPCR. The expression levels of *XM_011557236.1*, *XM_011554187.1*, and *XM_011563034.1* showed significant differences compared with others, such as *PxylCYP9f2*, *PxylGSTe1*, and *PxylGSTs1* via BLAST (+ 2.17.0) searching in the NCBI database, respectively. The changes in expression levels for *PxylCYP9f2*, *PxylGSTe1*, and *PxylGSTs1* were 1.59-, 5.25-, and 4.45-fold, with log_2_-fold change values of 0.67, 2.39, and 2.08, respectively. Hence, *PxylCYP9f2*, *PxylGSTe1*, and *PxylGSTs1* were identified as candidate *PxylODEs* that responded to 2,3-dimethyl-6-(1-hydroxy)-pyrazine and were used in further analysis.

**Figure 2:**
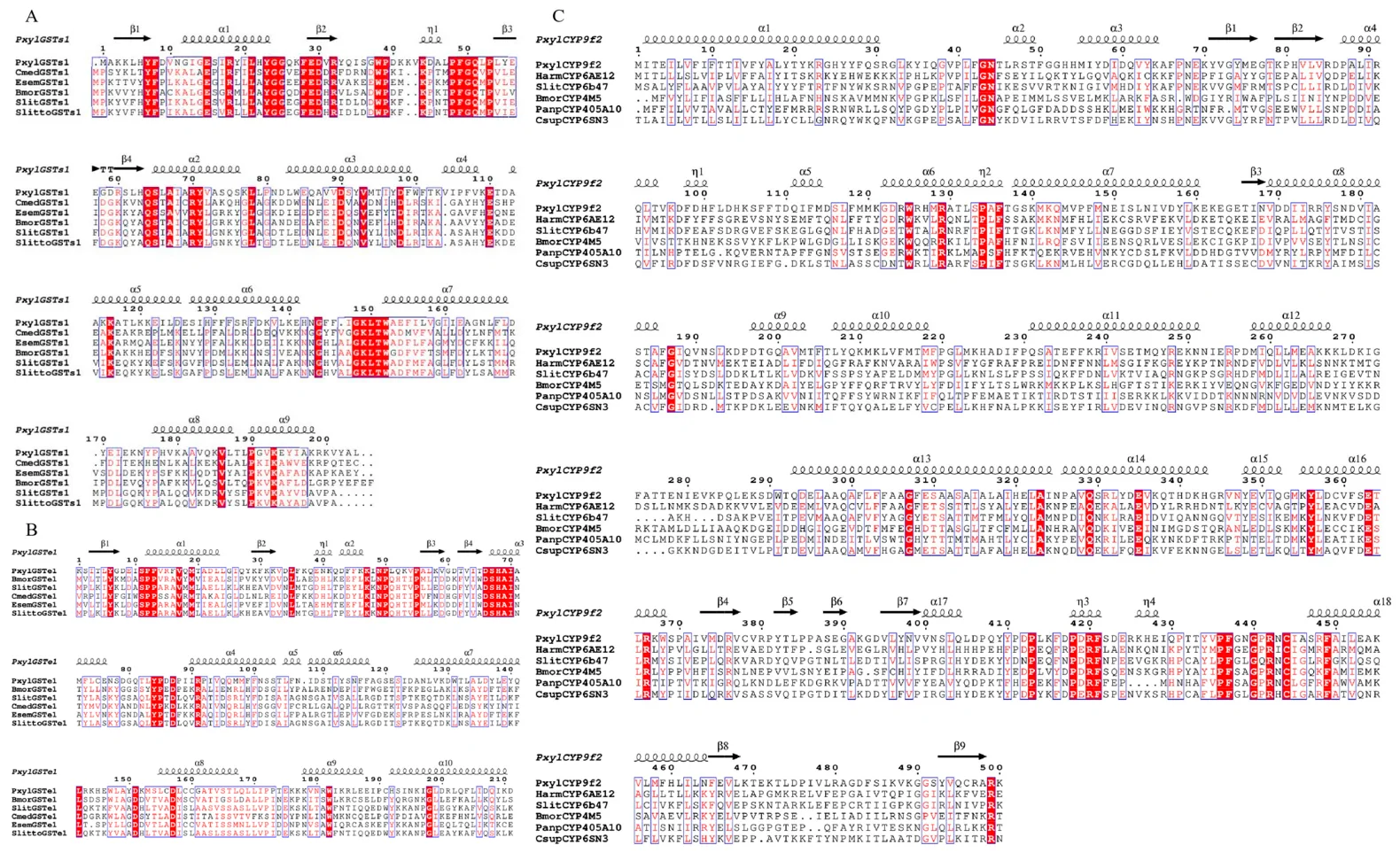
Multiple sequence alignment of the three candidate PxylODEs. The amino acid residues labeled in red represent similarity; the blue background frames represent the sites of amino acids identified; and the columns framed in blue mean that >70% of residues are similar according to physicochemical properties. (**A**) *PxylGSTs1*; (**B**) *PxylGSTe1*; (**C**) *PxylCYP9f2*.

Three selected *PxylODEs* were cloned: the basic properties of the encoded proteins were analyzed using the bioinformatics analysis websites of Expasy (https://web.expasy.org/protparam/, accessed on 1 May 2025) and SignalP (https://www.cbs.dtu.dk/services/SignalP/, accessed on 1 May 2025). The sequence lengths of *PxylCYP9f2*, *PxylGSTe1*, and *PxylGSTs1* were 520, 232, and 204 amino acids (Figure 2). The calculated molecular weights were 59.96, 26.86, and 23.65 kDa, with isoelectric point values of 8.35, 7.59, and 8.50, respectively. In addition, the similarities of *BmorGSTs1*, *CmedGSTs1*, *EsemGSTs1*, *SlitGSTs1*, and *SlittoGSTs1* between *PxylGSTs1* were 31.61%, 36.55%, 32.14%, 31.47%, and 32.29%; this was determined by comparing amino acid sequences using Blast in the NCBI (Figure 2A). Simultaneously, the amino acid sequence similarities of *PxylGSTe1* and *BmorGSTe1*, *EsemGSTe1*, *CmedGSTe1*, *SlittoGSTe1*, and *SlitGSTe1* were calculated as 33.85%, 33.81%, 25.96%, 28.27%, and 28.80%, respectively. As shown in Figure 2C, the similarities between *PxylCYP9f2* and *SlitCYP6b47*, *HarmCYP6AE12*, *CsupCYP6SN3*, *BmorCYP4M5*, and *PanpCYP405A10* were 27.96%, 28.79%, 30.88%, 24.36%, and 20.97%.

**Figure 3:**
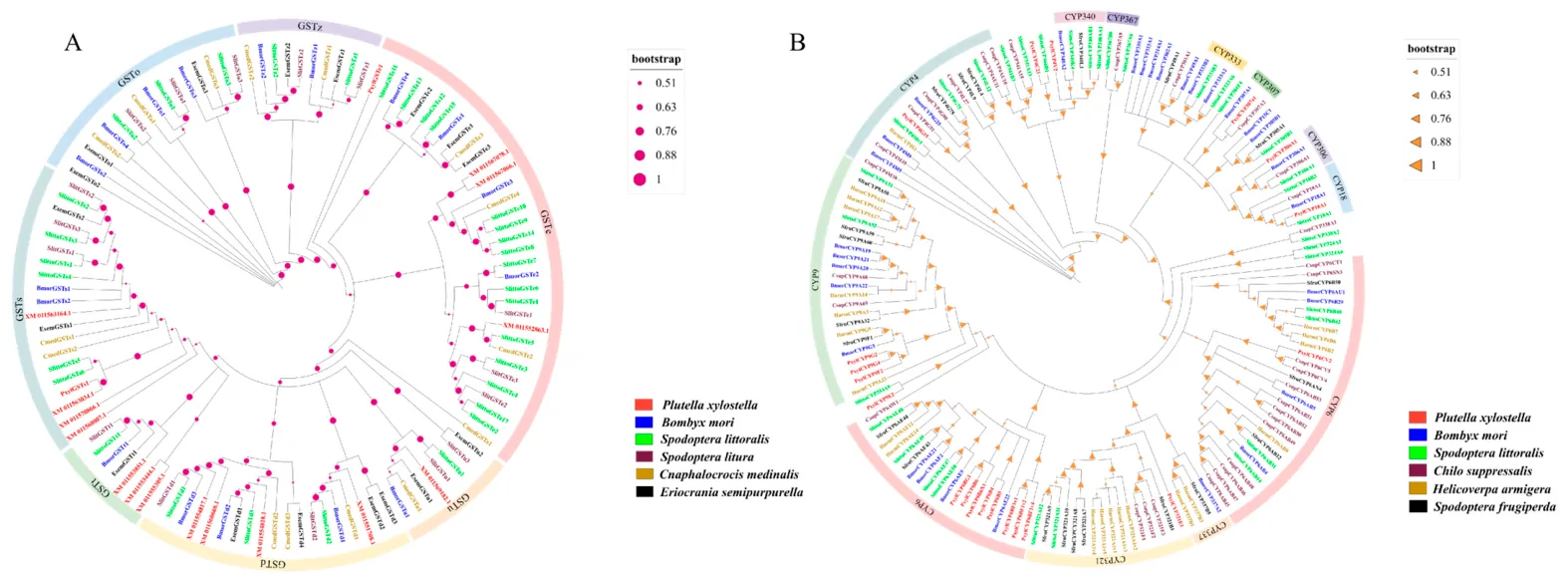
Phylogenetic tree analysis of PxylODEs. Numbers at the edges are local support values (0–1) calculated using the Shimodaira–Hasegawa test implemented in FastTree. (**A**) The data based on GST sequences from *P. xylostella* (red), *B. mori* (blue), *S. littoralis* (green), *S. litura* (purple), *C. medinalis* (orange), and *E. semipurpurella* (black). (**B**) The data based on CYP sequences from *P. xylostella* (red), *B. mori* (blue), *S. littoralis* (green), *Chilo suppressalis* (purple), *H. armigera* (orange), and *S. frugiperda* (black).

A phylogenetic tree was constructed based on the protein–protein BLAST homology of PxylODEs compared to five Lepidopteran ODEs; 114 (Supplementary Material: File S1) and 60 sequences were used to analyze *PxylGSTe1, PxylGSTs1* and *PxylCYP9f2* in this study (Supplementary Material: File S2). The homologies between *PxylGSTs1* and *SlittoGSTs5* and *SlittoGSTs6* were 60.30% and 49.02; the values between *XM_011563164.1* and *BmorGSTs1*, *BmorGSTs2*, *SlittoGSTs1*, and *SlittoGSTs4* were 59.31%, 63.73%, 58.00%, and 61.76%, respectively. These two genes were speculated to belong to the GST family (Figure 3A). The homology between *PxylGSTe1* and *BmorGSTe1* was 33.85%, with a value of 35.43% compared with *SlittoGSTe12*. Moreover, other ODEs showed high homology, such as *S. littoralis* and *B. mori*. The homology between *XM_011567078.1* of *P. xylostella* and *BmorGSTe3* was 53.49%; the value between *XM_011552863.1* and *SlittoGSTe5* was 52.44%; and it was speculated that these two genes belonged to the GSTe family. *XM_011569182.1* showed 66.26% homology to *BmorGSTu1*, identified as the GSTu family. Simultaneously, the homologies between *XM_011551708.1* and *BmorGSTd1*, *SlittoGSTd2* were 69.27% and 66.94%; the values between *XM_011554818.1* and *SlittoGSTd3* and *BmorGSTd2* were 82.41% and 85.92%; and values of 70.00% and 70.14% were identified between *XM_011554817.1* and *BmorGSTd3* and *SlittoGSTd1*. These three genes belonged to the GSTd family. *XM_011553851.1* was determined to belong to the GSTt family due to its homology of 54.50% and 61.26% compared with *BmorGSTt1* and *SlittoGSTt1*. As shown in Figure 3B, most PxyCYPs were clustered at the same branch as some CYPs of *S. littoralis*, *B. mori* and *Chilo suppressalis*; the homologies between *PxylCYP306A1* and *CsupCYP306A1*, *BmorCYP306A1*, and *SlittoCYP306A1* were 70.69%, 71.99%, and 74.40%, respectively, all of which belong to the A subfamily of CYP306. In addition, the similarities between *PxylCYP18A1* and *CsupCYP18A1*, *BmorCYP18A1*, and *SlittoCYP18A1* were 77.65%, 79.78%, and 84.56%, respectively, and were determined to be homologous genes of CYP18A1 in different species and belonging to the CYP18 family of cytochrome P450.

### 3.2. Expression Patterns of Candidate PxylODEs

**Figure 4:**
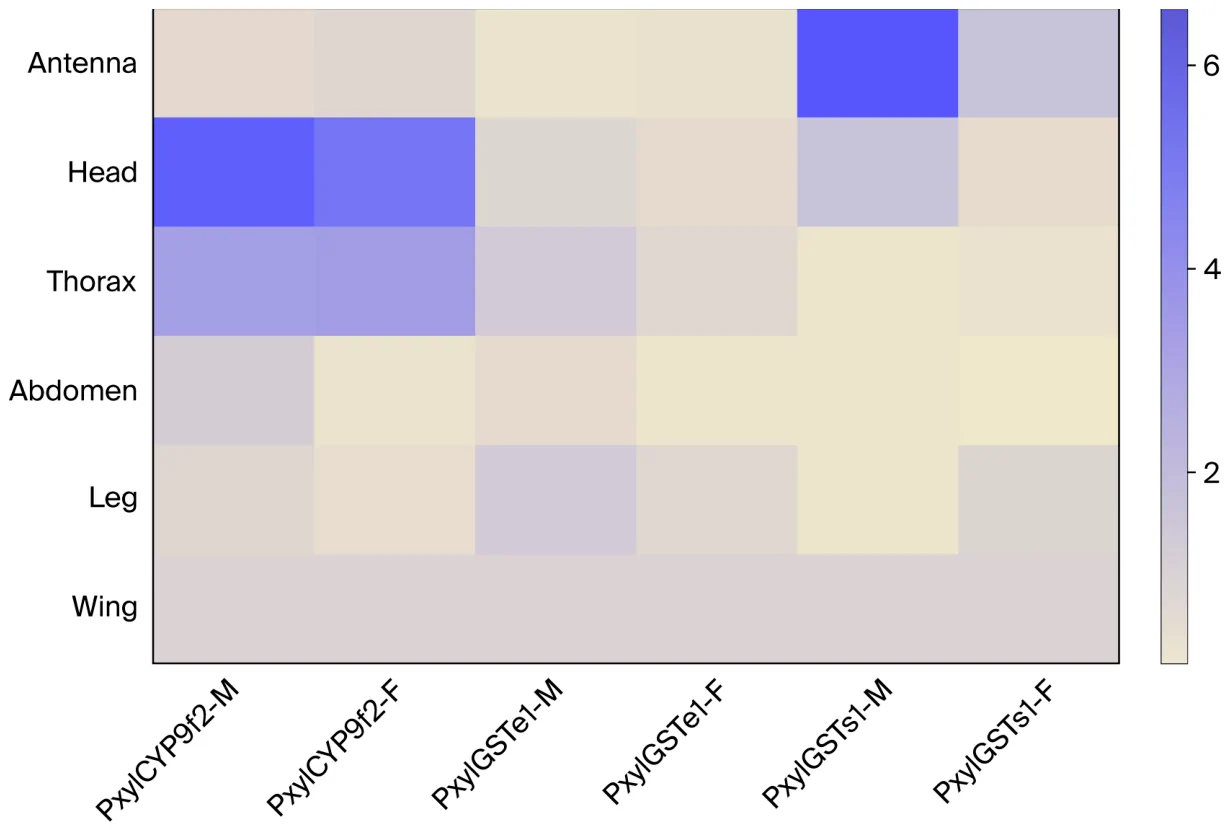
Expression pattern analysis of three candidate PxylODEs in different tissues (antenna, head, thorax, abdomen, leg, and wing) of female and male *P. xylostella* (n = 90). PxylCYPf2-M and PxylCYPf2-F were *PxylCYPf2* in male and female adults; PxylGSTe1-M and PxylGSTe1-F were *PxylGSTe1* in male and female adults; PxylGSTs1-M and PxylGSTs1-F were *PxylGSTs1* in male and female adults.

As shown in Figure 4, *PxylGSTs1* had significant expression levels in the antennae of *P. xylostella* adults, with 3.60-fold higher levels in male than female antennae. The expression level of *PxylGSTs1* in the head section of male adults was 3.00-fold higher than in females; levels in the abdomen were 2.00 times greater. However, the expression levels of *PxylGSTs1* in the female thorax and leg sections were 1.60-fold and 3.80-fold higher than in males, respectively, with no significant differences between female and male wings.

For *PxylCYP9f2*, its expression level in *P. xylostella* adults was identified in the following order from highest to lowest: the head, thorax, wing, antenna, abdomen, and leg. There were no significant differences in the thorax and wings between females and males. Simultaneously, *PxylCYP9f2* levels in the antennae of female adults were 1.20-fold greater than in males, while expression levels in the head, abdomen, and leg of male adults were 1.20-, 4.30-, and 1.50-fold higher than in females. Moreover, expression levels were higher in almost all sections of male *P. xylostella* adults than females; levels in the head, thorax, abdomen, and leg were 1.40-, 1.70-, 4.30-, and 1.80-fold higher than in females.

### 3.3. Structure Modeling and Molecular Docking

To evaluate the stability of 2,3-dimethyl-6-(1-hydroxy)-pyrazine binding with the three PxylODE proteins during the simulation process, the interaction frequency between the RMSD of ODE main strains and the compound was monitored. As shown in Figure S3, the binding between PxylGSTS1 and 2,3-dimethyl-6-(1-hydroxy)-pyrazine remained stable overall throughout the entire simulation cycle. However, high volatility of PxylCYP9F2 and PxylGSTE1 was exhibited at 80 ns, indicating a significant change in conformation that occurred; the interaction frequency reflected relevant information. To further illustrate the binding affinity of the pyrazine ligand with the three PxylODEs, the binding energy for the last 10 ns was calculated using MM-PBSA (molecular mechanics Poisson–Boltzmann surface area). It was found that binding energies for PxylGSTS1, PxylGSTE1, and PxylCYP9F2 were −26.52, −26.52, and −17.39 kcal/mol, respectively. These results indicate that the binding between 2,3-dimethyl-6-(1-hydroxy)-pyrazine and PxylGSTS1 was superior to that with PxylCYP9F2 and PxylGSTE1 from an energy perspective. In general, molecular dynamics simulations revealed the stability and interaction modes of the binding pocket interactions between pyrazine and three candidate PxylODEs.

**Figure 5:**
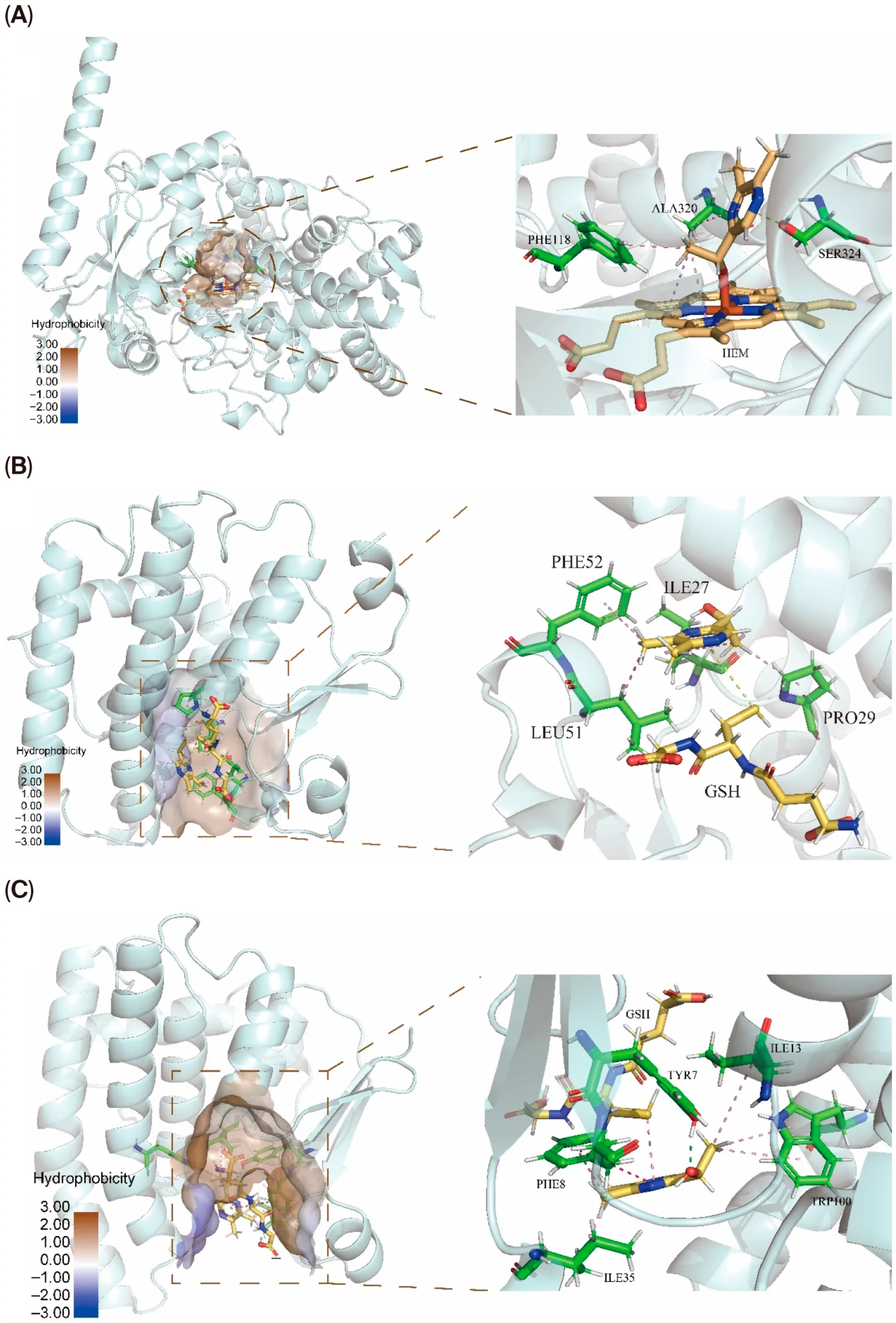
Binding modes and key residues between 2,3-dimethyl-6-(1-hydroxy)-pyrazine and three candidate PxylODEs. (**A**) PxylCYPF2; (**B**) PxylGSTE1; (**C**) PxylGSTS1.

Simultaneously, molecular docking was used to illustrate the key interaction residues and binding energies between 2,3-dimethyl-6-(1-hydroxy)-pyrazine and three PxylODE proteins (Figure 5). It was revealed that pyrazine was bound with the hydrophobic pocket formed by the PHE118 (Phenylalanine 118), ALA320 (*α*-Linolenic acid 320), and SER324 (Serine 324) residues of PxylCYP9F2 with a binding energy of −10.7 kcal/mol (Table 1). The hydrophobic interaction occurred between methyl groups in pyrazine and PHE118 and ALA320. Simultaneously, the lone-pair–π interaction formed between the nitrogen-containing pyrazine six-membered ring and the lone-pair electrons of the oxygen atom on the SER324 side chain, together with the coordination bond between the hydroxyl group and the iron atom on the HEM of the P450 coenzyme. Then, a hydrophobic interaction was established between pyrazine and the hydrophobic pocket of ILE27 (L-Isoleucine 27), PRO29 (Proline 29), LEU51 (Leucine 51), and PHE52 on PxylGSTE1 with a binding energy value of −7.90 kcal/mol. The π–sulfur interaction was identified between the nitrogen-containing six-membered ring and the sulfur atom on coenzyme GSH (glutathione). Furthermore, a non-covalent interaction was found between pyrazine and the hydrophobic pocket of PxylGSTS1; the occurrence of a stable conformation was mainly caused by hydrophobic interaction. The binding energy was −5.82 kcal/mol, the hydrogen bond was found between the pyrazine hydroxyl and side chain of TYR7 (Tyrosine 7). In addition, the π–π stacked hydrophobic interaction was established between the nitrogen-containing six-membered ring and PHE8; the π–sulfur interaction was determined between the compound and the sulfur atom on coenzyme GSH, which was similar to PxylGSTE1. In summary, the binding affinity between pyrazine and PxylCYP9F2 was much higher than that of PxylGST enzymes; it was speculated that P450 enzymes usually fulfill a role in the first stage of metabolic processes, whereas GST enzymes are involved in the second stage of metabolism and modification.

### 3.4. Ligand-Binding Properties of PxylODEs with Respect to 2,3-Dimethyl-6-(1-Hydroxy)-Pyrazine

**Figure 6:**
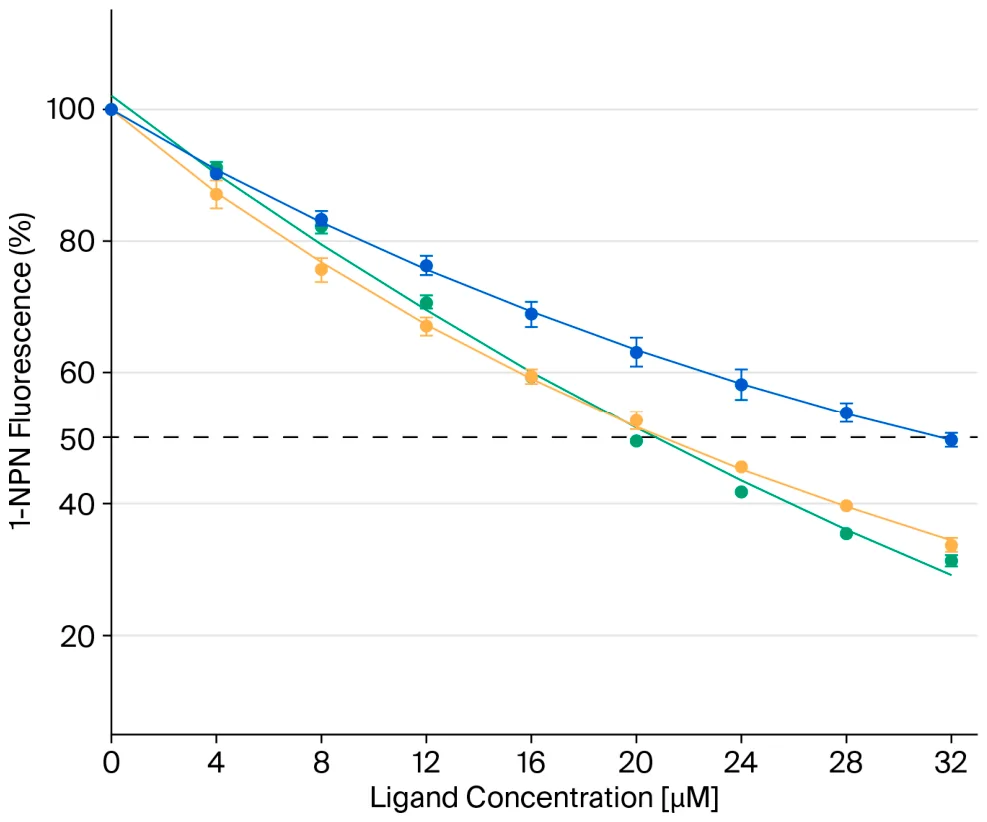
Comparison of binding properties of the three candidate PxylODEs with respect to 2,3-dimethyl-6-(1-hydroxy)-pyrazine. (Blue) PxylGSTS1; (orange) PxylGSTE1; (green) PxylCYPF2. The bars represent mean standard error (n = 90), and dashed line indicated the 1-NPN fluorescence value of 50%.

Firstly, ligand binding was strengthened with increasing concentrations of 1-NPN (Figures S4–S6); it was found that the Kd values (dissociation constant) of PxylCYP9F2, PxylGSTE1, and PxylGSTS1 binding with 1-NPN were 4.88, 4.73, and 5.43 μmol/L, respectively. Hence, 1-NPN can be used for subsequent competitive ligand-binding assays to determine the binding affinity of PxylODEs to 2,3-dimethyl-6-(1-hydroxy)-pyrazine. PxylGSTS1 had a higher binding affinity to 2,3-dimethyl-6-(1-hydroxy)-pyrazine with a Ki value (inhibition constant) of 11.63 μmol/L (Figure 6). Subsequently, the Ki values of PxylCYP9F2 and PxylGSTE1 were calculated to be 14.79 and 15.32 μmol/L, respectively. Then, we inferred that PxylGSTS1 may play an important role in responding to the repellent activity of 2,3-dimethyl-6-(1-hydroxy)-pyrazine against *P. xylostella* adults.

### 3.5. Validation of the Function of PxylGSTS1 in Response to 2,3-Dimethyl-6-(1-Hydroxy)-Pyrazine

Based on the data obtained, PxylGSTS1 was chosen to validate the binding site with 2,3-dimethyl-6-(1-hydroxy)-pyrazine. Firstly, we screened possible sites such as PHE167, ILE35, VAL108 (Valine 108), TRP100 (Tryptophan 100), PHE8, and TYR7 using molecular dynamic analysis; TYR7, PHE8, ILE13, ILE35, and TRP100 were screened via DS 2021 (Discovery studio) software. The four sites, TYR7, PHE8, ILE35, and TRP100, were selected to be mutated to alanine (ALA), with the binding energy shown in Table S18. It was found that the binding energy of the mutation sites TYR7 and TRP100 significantly decreased; the hydrogen bond was formed by the hydroxyl group on pyrazine and the side chain of TYR7. Hence, tyrosine at position 7 of PxylGSTS1 was selected to assay site-directed mutation to TYR7Ala, named PxylGSTS1^Y7A^.

**Figure 7:**
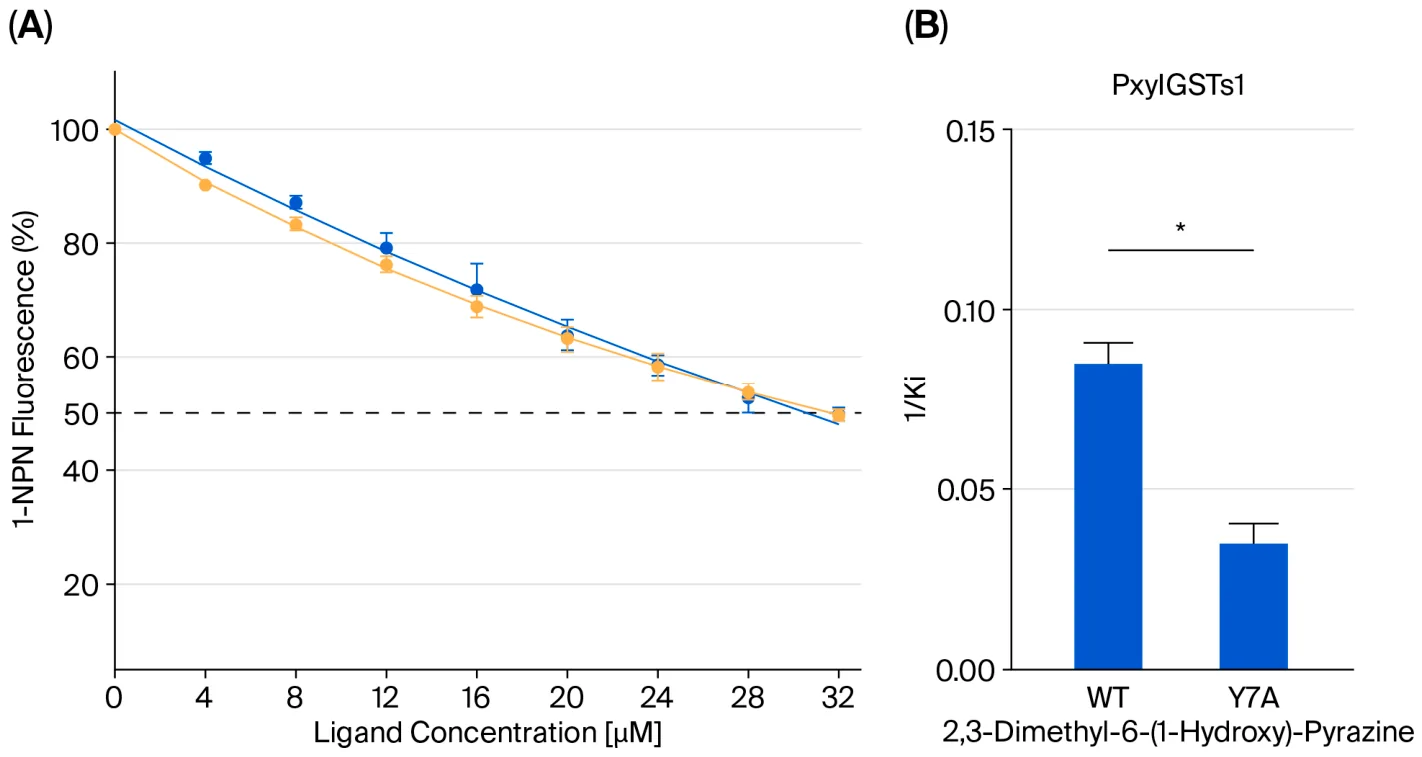
Comparison of binding properties of PxylGSTS1 and PxylGSTS1^YA7^ mutations with respect to 2,3-dimethyl-6-(1-hydroxy)-pyrazine. The bars represent mean standard error (n = 90), and * denoted significant difference between wild and mutant *P. xylostella* at a *p* <0.05. (**A**) Fluorescence competitive binding curve, (blue) PxylGSTS1; (orange) PxylGSTS1^YA7^. (**B**) Analysis of significant differences. Dashed line indicates the 1-NPN fluorescence value of 50%.

Subsequently, the Kd value of mutation-type PxylGSTS1^Y7A^ binding with 1-NPN was determined to be 6.73 μmol/L (Figure S7), showing a significant decrease in the binding affinity compared with the wild type (5.43 μmol/L). It was shown that the TYR7 site may play an important role in the binding procedure of PxylGSTS1 and the ligand. Then, the Ki values of PxylGSTS1 and PxylGSTS1^Y7A^ binding measured with 2,3-dimethyl-6-(1-hydroxy)-pyrazine were determined as 11.63 and 27.86 μmol/L, respectively (Figure 7). There was significant difference between the wild and mutation type, indicating that the mutation of the TYR7 site has a clear influence on the binding ability of the protein and ligand.

## 4. Discussion

Currently, *P. xylostella* is one of the most significant pests, causing serious damage to cruciferous crops and exhibiting resistance to many chemical insecticides. We found that 2,3-dimethyl-6-(1-hydroxy)-pyrazine has clear negative effects on its olfactory system, and PxylOR31 and PxylPBP2 were identified as the main molecular targets [16,17,18]. After the pyrazine was volatilized to the *P. xylostella* antennae, this compound would be identified and combined by the PxylPBP2. Then, this complex was transferred to PxylOR31, which would be activated and to transferred to the lymph; then, the OR can be activated to guide related insect behavior, changing the chemical signal into an electrical signal to be conducted to the nervous center [52,53]. Meanwhile, the redundant compounds were removed by the ODEs, which may have had several effects on the activity of pyrazines. Based on genomic and transcriptomic sequencing, multiple studies have shown that the expression of ODEs in insects is an effective strategy of degrading the external environment, which plays an important role in the development of resistance to insecticides [54,55]. It was found that 25 esterases, 56 P450s, 31 GSTs, and 9 UGTs are expressed in *Drosophila melanogaster* antennae; these largely uncharacterized enzymes are prime candidates for ODE functions. Cytochrome P450s are often associated with xenobiotic degradation, which is required to rapidly hydrolyze odorants and has been suggested to protect insect olfactory receptor neurons against volatile natural toxins or insecticides [56,57]. Esterases belong to the hydrolase enzyme family and play critical regulatory roles in pyrethroid degradation; it has been determined that resistant insects are also known to contain pyrethroid hydrolases [58,59]. Transcriptomics-based analysis of *Anopheles albimanus* revealed differential resistance against α-cypermethrin and deltamethrin; the cytochrome P450 CYP9K1 was overexpressed 14 and 68 times [60].

ODE is an important tool to recognize, as it degrades volatile compounds in the antennae of insects, which is an indispensable part of the olfactory system. More than 50 CYPs out of 87 CYPs were expressed in the *Drosophila* antenna, which is the primary olfactory tissue [61]. Moreover, the role of specific CYPs in degrading particular odorants and pheromones in vitro was shown, while only one CYP gene directly affected olfactory neuron responses to a particular odorant in vivo in other insects [30,62]. Cyp4p1 and Cyp6d5 are broadly expressed in *Drosophila* antennae but are not expressed by olfactory neurons, which have long been thought to mediate odorant degradation. Several CYPs in the antennae of *Dendroctonus ponderosae* were capable of removing terpenoids and detoxifying host terpenoids to overcome plant defenses [28,63]. It has been reported that insect CXEs could degrade ester, amide, and carbamate bonds of many plant volatiles, insect pheromones, hormones, and pesticides, which are highly expressed in the antennae of male silkmoths [24]. SlCXE7 and SlCXE10 are predominantly expressed in the antenna sensilla of *S. littoralis* and play key roles in the degradation of pheromones and plant volatile components. In our study, fifty-five PxylODEs were determined via RNA-seq in the antennae of *P. xylostella*; we obtained three candidate ODEs responding to the repellent activity of 2,3-dimethyl-6-(1-hydroxy)-pyrazine.

Understanding the binding style, site, and energy between the ligand and receptor is essential for revealing the molecular mechanism of insecticidal activity or resistance; molecular docking and fluorescence competitive binding tests are effective tools. The binding between 2,3-dimethyl-6-(1-hydroxy)-pyrazine and PxylGSTS1 is a hydrophobic interaction where the energy is −5.82 kcal/mol. It was calculated via a fluorescence competitive binding experiment, where PxylGSTS1 had a higher binding affinity to pyrazine with a Ki value of 11.63 μmol/L, validated with a mutation to the TYR7 side chain. It was found that SlCXE7 efficiently hydrolyzed Z3-6:Ac as a green volatile emitted by host plants; the K_cat_ and specific activity were 36 and 2.4 × 10^4^ mol/s, respectively [64]. *Cis*-jasmone was bound in the hydrophobic pocket of the LoryGSTd1 protein and interacted with amino acid residues ILE103, ARG68, LEU99, THR72, ALA69, and GLU66 via eight hydrophobic interactions; the binding energy was −5.6 kcal/mol [65]. The mean binding energy between BgCCE021d1 and (Z)-3-hexenyl hexanoate, (E)-2-hexenyl acetate, and geranyl acetate was −4.3 kcal/mol, which showed higher affinity than BgCCE021e3 with a value of −3.6 kcal/mol [66]. The binding energy between BminGSTd3 and undecanol was −31.96 kcal/mol; E64 and S65 were deduced as significant binding sites; and residues included T6, G8, S9, S10, P11, L33, T51, I52, P53, E64, S65, R66, Y105, Y113, F117, F203, and F207 within 4 Å [67]. It was determined that the binding energies between *α*-pinene and TyunCYP4G2 and TyunCYP6DF1 were −5.46 kcal/mol and −5.18 kcal/mol, and the amino acid binding sites were tyrosine 4 and arginine 502, respectively [68]. Thus, during the treatment period, pyrazine can change the expression level of several PxylODEs, which may mediate the degradation of pyrazines.

Hence, PxylGSTS1 can be used as an interesting molecular site to develop novel repellent ingredients, a creative strategy for the green control of *P. xylostella*. Finally, the contents of this study are limited, as most results regarding PxylODEs characteristics were obtained from MD and other simulation methods, in addition to site-directed mutation. To develop the pyrazines as novel active ingredients or lead compounds based on these ODEs, more in-depth validation experiments like RNAi are essential in future research.

## 5. Conclusions

In summary, our findings highlight several fundamental questions regarding the degradation of volatile compounds in the antennae of *P. xylostella*, especially the components with antifeedant or repellent activity. *PxylGSTs1* was identified as the main receptor responding to 2,3-dimethyl-6-(1-hydroxy)-pyrazine with clear repellent activity; it is highly expressed in male antennae. Simultaneously, the binding between PxylGSTS1 and pyrazine is mainly hydrophobic, and the side chain of TYR7 was validated via site-directed mutation. These data provide novel insights into the creation of new active insecticidal ingredients for the green control of Lepidoptera insects, especially PxylGSTS1, which can be used as a molecular site.

## Figures and Tables

**Table 1:** Binding energies and interaction residues between 2,3-dimethyl-6-(1-hydroxy)-pyrazine and the three PxylODE proteins.

Complex	Binding Energy (kcal/mol)	Interaction Residues
PxylCYP9F2-pyrazine	−10.7	PHE118, ALA320, SER324, HEM
PxylGSTE1-pyrazine	−7.9	ILE27, PRO29, LEU51, PHE52, GSH
PxylGSTS1-pyrazine	−5.8	TYR7, PHE8, ILE13, ILE35, TRP100, GSH

## Data Availability

Transcriptome datasets are also available on the NCBI website; the accession number is PRJNA987853.

## Supplementary Materials

The following supporting information can be downloaded at https://www.mdpi.com/article/10.3390/insects17101070/s1: Figure S1: Original nucleic acid electrophoresis images for the *PxylGSTe1*, *PxylGSTs1* (A) and *PxylCYP9f2* (B); Figure S2: Original protein electrophoresis images for the PxylGSTE1, PxylGSTS1 and PxylCYP9F2 (M: Protein Marker, 1: supernatant of expression product, 2: purified protein, 3: precipitation of expression product, 4: purified protein, 5: precipitation of expression product, 6: purified protein); Figure S3: RMSD between PxylODE receptors and pyrazine ligand; Figure S4: Binding curves and scatchard plots (insert) of the fluorescence probe 1-NPN to PxylCYP9F2, indicated the Kd value was 4.88 ± 0.80 μmol L^−1^; Figure S5: Binding curves and scatchard plots (insert) of the fluorescence probe 1-NPN to PxylGSTE1, indicated the Kd value was 4.73 ± 0.97 μmol L^−1^; Figure S6: Binding curves and scatchard plots (insert) of the fluorescence probe 1-NPN to PxylGSTS1, indicated the Kd value was 5.43 ± 0.45 μmol L^−1^; Figure S7: Binding curves and scatchard plots (insert) of the fluorescence probe 1-NPN to PxylGSTS1^Y7A^, indicated the Kd value was 6.73 ± 1.68 μmol L^−1^; Table S1: Reverse transcription reaction system; Table S2: RT-qPCR primers of ODEs gene in *P. xylostella*; Table S3: RT-qPCR reaction system; Table S4: Amplification primers of candidate ODE genes; Table S5: Amplification of target fragment reaction system; Table S6: Sequences information used to phylogenetic tree analysis to PxylGST; Table S7: Sequences information used to phylogenetic tree analysis to PxylCYP; Table S8: Link reaction system of recycled products; Table S9: Reaction system of positive clone validation to single colony; Table S10: Prokaryotic expression primers of *P. xylostella* ODEs; Table S11: Double enzyme digestion reaction system; Table S12: Homologous recombination reaction system; Table S13: PCR reaction system of recombination product colony; Table S14: Site directed mutagenesis primer design; Table S15: Site directed mutagenesis PCR amplification reaction system; Table S16: Digestion reaction system; Table S17: Recombination reaction system; Table S18: Binding energy of 2,3-dimethyl-6-(1-hydroxy)-pyrazine between four PxylGSTS1 mutation sites; File S1: Sequences used to phylogenetic tree analysis to PxylGSTs; File S2: Sequences used to phylogenetic tree analysis to PxylCYPs.
