# Supplementary material for: Functional Analysis of Odorant-Degrading Enzymes Responding to the Repellent Activity of 2,3-Dimethyl-6-(1-Hydroxy)-Pyrazine Against Plutella xylostella
Source: Insects. 2026 Oct 16;17(10):1070. doi: 10.3390/insects17101070 (PMC13705310; doi:10.3390/insects17101070)
Supplement: Supplementary file 1 [file insects-17-01070-s001.zip › insects-4540032-supplementary.pdf]

Supplemental Information

Functional analysis of odorant-degrading enzymes responding to the repellent activity of 2,3-dimethyl-6-(1-hydroxy)-pyrazine against *Plutella xylostella*

Yanlu Zhou[a,b,1], Xianwen Wang[a,b,1], Buguo Wang[a,b], Zixian Wang[a,b], Qing Li[a,b], Haiqun Cao[a,b], Quan Gao[a,b,*]

[a] Key Laboratory of Agri-products Quality and Biosafety (Anhui Agricultural University), Ministry of Education, Hefei 230036, China

[b] Anhui Province Engineering Laboratory for Green Pesticide Development and Application, School of Plant Protection, Anhui Agricultural University, Hefei 230036, China

[1] These authors contributed equally.

[*] Correspondence: quangao@ahau.edu.cn (Q.G.)

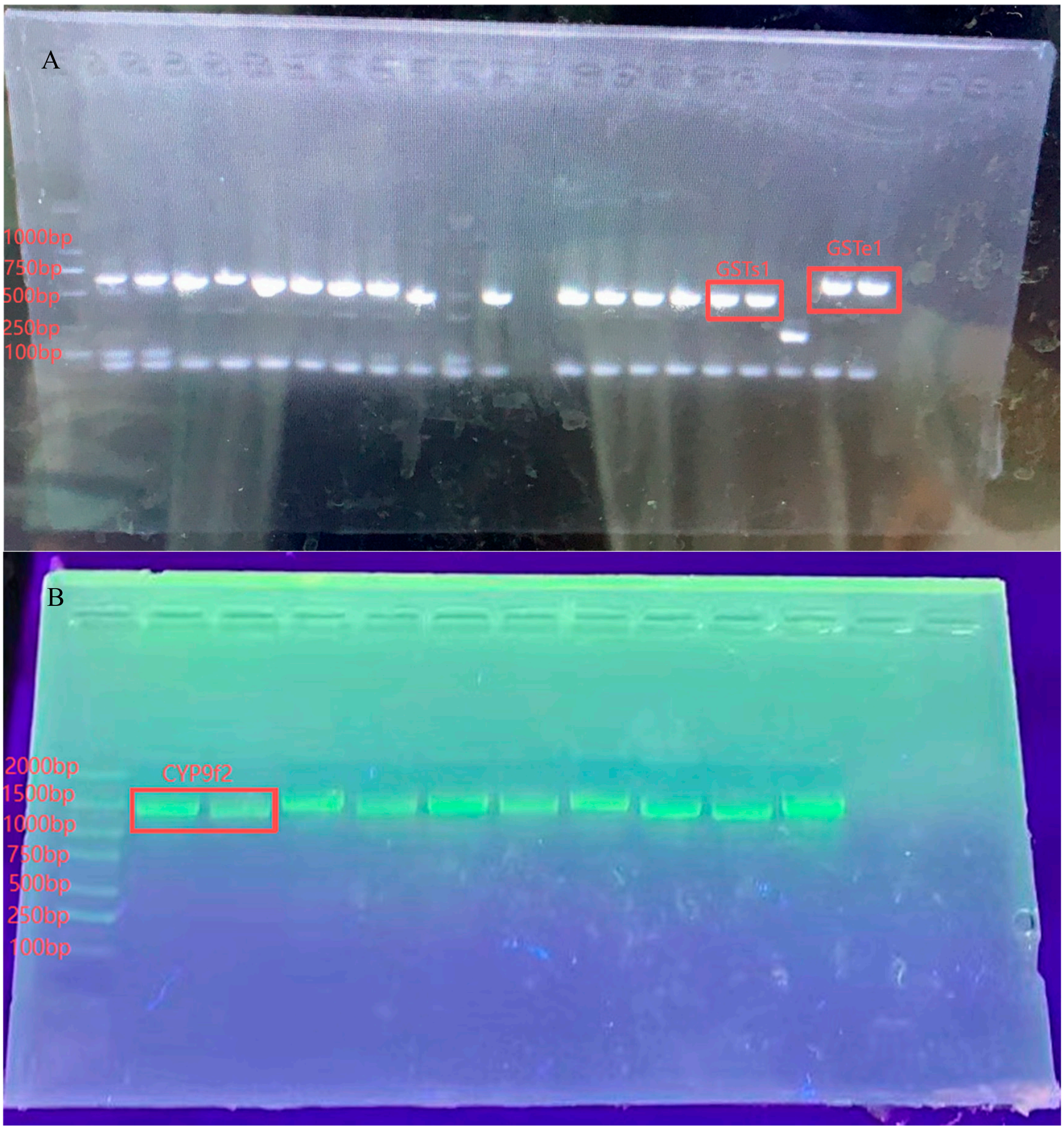

Figure S1 Original nucleic acid electrophoresis images for the *PxylGSTe1*, *PxylGSTs1* (A) and *PxylCYP9f2* (B)

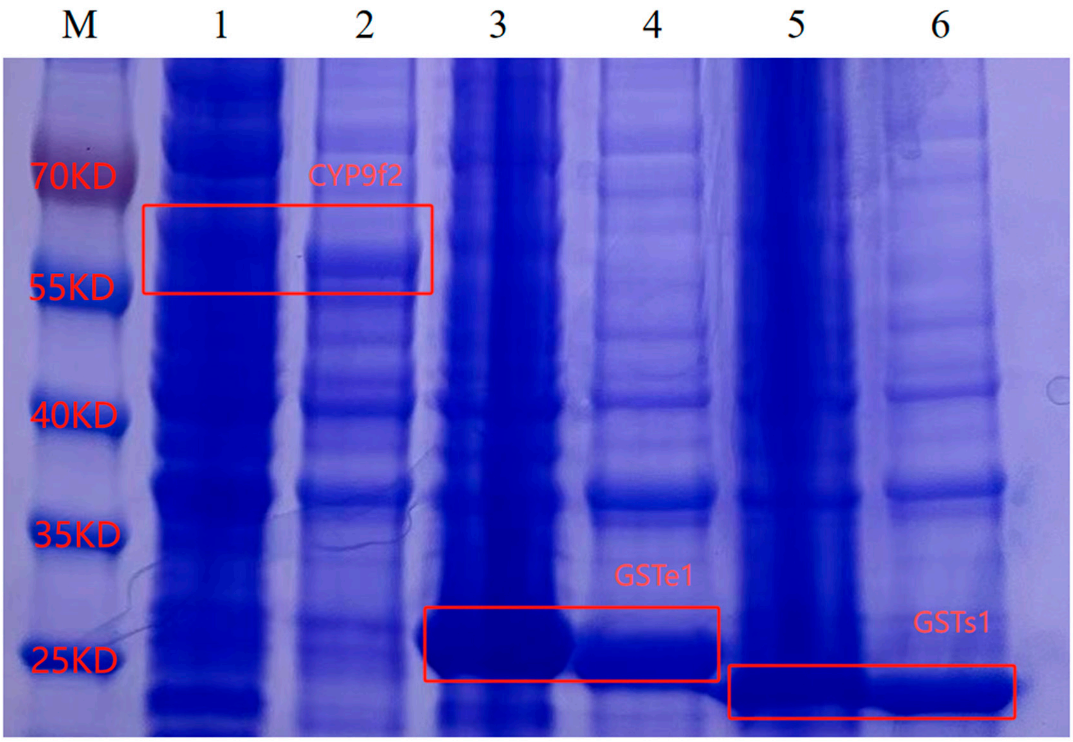

Figure S2 Original protein electrophoresis images for the PxylGSTE1, PxylGSTS1 and PxylCYP9F2 (M：Protein Marker, 1: supernatant of expression product, 2: purified protein, 3: precipitation of expression product, 4: purified protein, 5: precipitation of expression product, 6: purified protein)

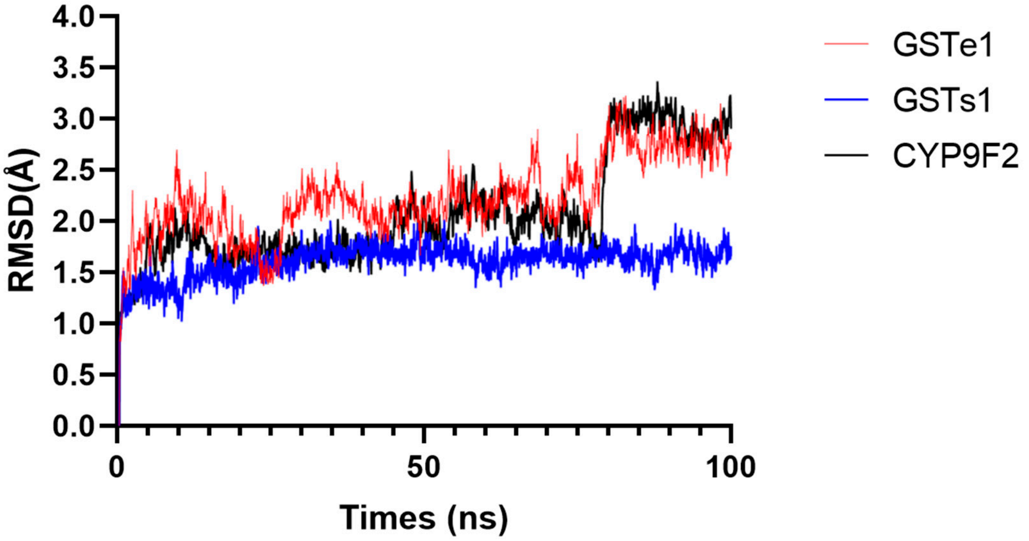

Figure S3　RMSD between PxylODE receptors and pyrazine ligand

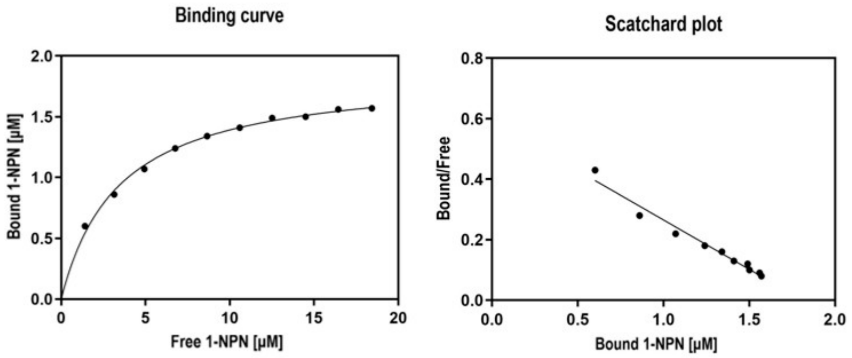

Figure S4 Binding curves and scatchard plots (insert) of the fluorescence probe 1-NPN to PxylCYP9F2, indicated the Kd value was 4.88 ± 0.80 μmol L^{-1}

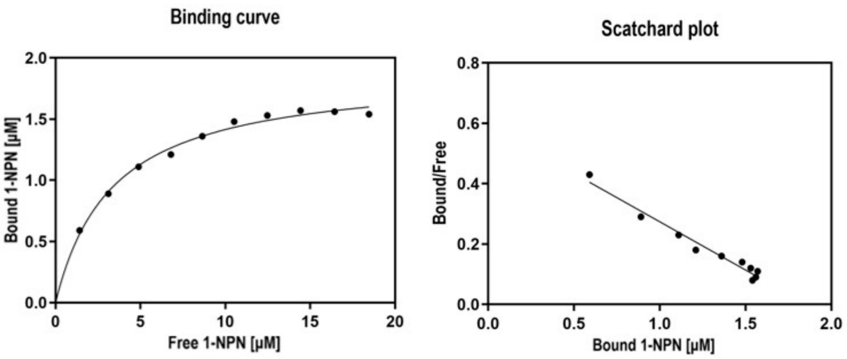

Figure S5 Binding curves and scatchard plots (insert) of the fluorescence probe 1-NPN to PxylGSTE1, indicated the Kd value was 4.73 ± 0.97 μmol L^{-1}

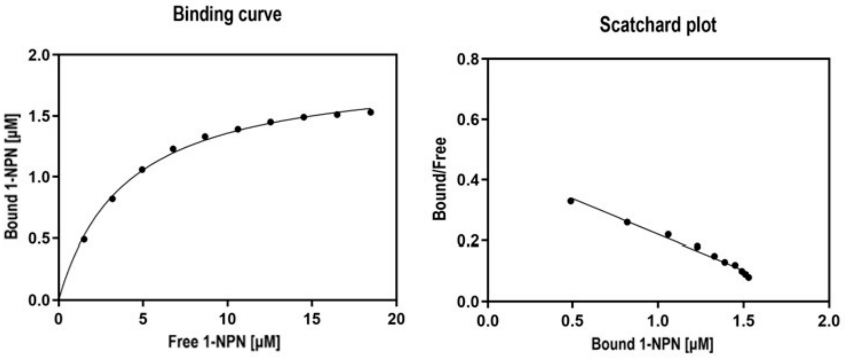

Figure S6 Binding curves and scatchard plots (insert) of the fluorescence probe 1-NPN to PxylGSTS1, indicated the Kd value was 5.43 ± 0.45 μmol L^{-1}

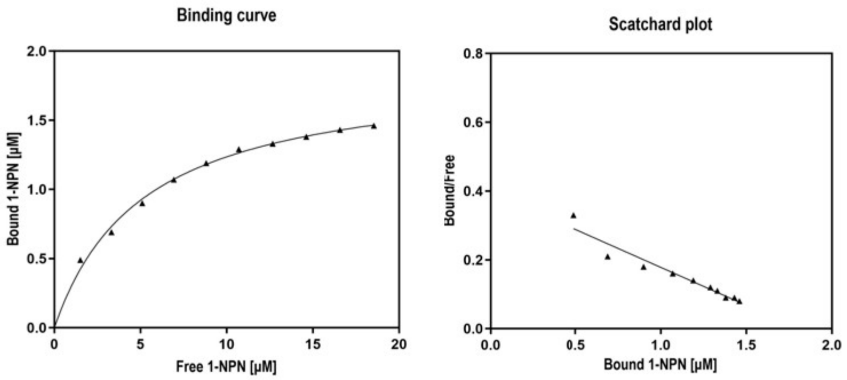

Figure S7 Binding curves and scatchard plots (insert) of the fluorescence probe 1-NPN to PxylGSTS1^{Y7A}, indicated the Kd value was 6.73 ± 1.68 μmol L^{-1}

Table S1 Reverse transcription reaction system

Reagents	Volume or weight
4×Hifair® advance fast super mix (No dye)	5 μL
Oligod $(T)_{18}$ primers（50 μmol L^{-1}) or Random primers (* for qPCR) (50 μmol L^{-1})	2 μL
Total RNA	1 μg
RNase-free water	To 20 μL

Table S2 RT-qPCR primers of ODEs gene in *P. xylostella*

Gene	Forward primer	Reverse primer
RPS3	TGGTTGCGAGGTGGTTGTCT	TAGTCGTTGCACGGGTCTCC
PxylCYP9f2	CGTATAAGAGAGGGCACTAT	CTTGGTCAATGTCGTATATCA
PxylGSTe1	CAATAGATGCGAACTTAGTCA	TCAGTCGGAGGTATCAATAG
PxylGSTs1	TCCATCCACTTCTTCTTCTC	GTTCTTCTCGATCTCGTAGT
XM_011563111.1	AACAAGACATACGAGCACTA	CTTCTCCTTCCAGGTTATGA
XM_011561822.1	TGTCTCTATAATTCCGAACCAT	AACGCTTCTTCAGTATCTCA
XM_011564179.1	GAGGAAGAAGCATTGAAGAT	GTAGACAGGATACCATCATTG
XM_011566241.1	GGTGAACACTTACGACTACG	GTTGATGGTGACTGTCTTGT
XM_011555600.1	CATCCAACTCATCAACTCAA	AGAGAACGAGAACATAGACA
XM_011552806.1	TTCTGTCGCATCGTCAATAG	CTTGTCCTGAAGGTCGTATT
XM_011562318.1	ATGGACATTGCTAAGACTGA	GGAGACCCTGTATCATATAGTTA
XM_011566243.1	CATTATCCAGGTTGCTGAAG	GTATTTGCTGTCCCTCTCTA
XM_011564113.1	ACACAAGAGACAGTAGATGA	GTTAGCATTAGGTTCAGACTT
XM_011555471.1	CTACAACTCGCCATTAGAAG	TCTATCGTAACACTGCTCTT
XM_011568645.1	ATATTCGTCTATCCGTCGTT	AATCCGTGTAGTCATAGTCTT
XM_011565532.1	GAATGAGAAGAATTATGGAGTGA	TGTCTATCATCATCTTGGTGA
XM_011550672.1	CGAATACAAGAGTGCTATCAT	TATTGAACTGTTTGCCGATT
XM_011560527.1	GAATACTGAACTGAGTGTGAT	CCATTATGCTCGTTATCTCTG
XM_011550033.1	CAACAAGAACGGAAGTAGAAT	TAGATTGAACCTCTGGATGG
XM_011563777.1	AATCCAGAATTGTATAATCCAGAA	TTACTTGAAGGCGAGCATAT
XM_011566807.1	GCTACGAGTCTATACACAATAATG	TTGAGAACTACTTCCAGATCC
XM_011567156.1	CACATCTACAACCACTTCAA	TTTAGCACATTTCTATCAACAAAG
XM_011551435.1	AACATTCACGAGATATTCCAA	ATTAGCAGAGACAGTTCCTT
XM_011552772.1	TTACAATTACTGCGTTCGTTA	CATTCGGAAAGTCTTCATAAATC
XM_011560826.1	AAGATGTGGTTGAGACTACT	CATATTCGGTGTTTCCTTCA
XM_011568662.1	ACTAGGAAGTGAATTAGTTGTG	GCCATCAGTGAAGGTTATAC
XM_011552299.1	CAAGTGAAAGTGCTGAAGAA	TTGCTTAATCTGGAGTTCGT
XM_011557052.1	GAAGAACTCTGAGAACAATCC	CTGTCTGGAAGAAGTTGAAG

XM_011550098.1	ACTCACGGAACAAGAGATTA	CTGCCAATTAGGTAGATGTG
XM_011563766.1	TTATCCAGATCCAGATGTGTT	CAGTGAAGTTGCGTAGAATG
XM_011551945.1	ACCGTTCTACAACAATACAG	GTCGTCCATATCTTTCATCAT
XM_011553682.1	CCTAAATGCGGTTTCCAAAT	TGTAGCGAATCCAACTGTAT
XM_011557062.1	ATGTTCTACCTCATCGTCAA	GTTGTCTTTCTCGTTTACCAT
XM_011560375.1	AAGAAGACACGGATGATGAA	GCCAGTAGCATTAGGAAGTA
XM_011557040.1	CGTTCTTCCACTCAGTATTG	GTTATGTAGTTGCTCTTCTTCA
XM_011568507.1	CTCATCTTCACCACCAACTA	CTCCAGCAACAGATCCATAA
XM_011553684.1	TGGATTGTGTTGCTAATGAG	CAGAACTGACCGAATTGTAG
XM_011552652.1	GTGATTCTGCTAGTGGCTAT	CTTCCGATATGTCCGTGATA
XM_011562457.1	CTCATCTTCAACTTCACCAA	AACTCTTCCTTCTTCTTCTTG
XM_011557669.1	CCAAGGACTACTACTACTTCA	TTGACGATGAGGTAGAACAT
XM_011559761.1	TGGAACAAGTCAGTCTCTAC	GATGGTGATCTCAACGATTAC
XM_011553444.1	ACTGTAACATTCTATCCAAGAGA	CGCTAGGTTGTAGACATACT
XM_011563164.1	GTCCACTACGAAGAAGATGA	TTGTTCCTCTGCACTATGTC
XM_011555305.1	GACAACGAAGATGTGAAGAG	TCATAGTCTCAACTGGATTATTC
XM_011552863.1	ACTAATACTGTATGTGAGTGATG	CTCGTAAGACTGGATTGATG
XM_011567066.1	CCTGTTCACACAATTCCTTTA	ATCCTTTGGGTATAGAGAGTC
XM_011554817.1	CTCGGAGATGAGAAGTATGC	GCAGTAGATTTGACCAACTC
XM_011553851.1	TTGCCATAATTCGGTACTTG	ACTGAACATAACAACTCTGAAG
XM_011567078.1	GACAAATACGGCAAGAGTGA	TCGGTTATGATGAGGAAGGT
XM_011570866.1	TGAAGATAGAAGGTGATGATGA	GGTCTACTACACTACTGAAGG
XM_011554818.1	CTCACATACCTCGTCAACAA	GTCTACCTTGGCTAACTTCT
XM_011551708.1	GACAACGGATTCATCTTATGG	GGCTGTAAAGGTTCAAATATCT
XM_011569182.1	CCTCAGTGAAAGTAATGCTAT	GTAGTACGATGCCAAGTTAA
XM_011559027.1	CACTGGAATCATTGGTTACAT	AGTTCAGCCTTATCCTCATC
XM_011568007.1	CTAAGTCCATCCATGAGTTC	TCGTTGAGTTGTTTGTAGTT
XM_011560668.1	AGCGATATTGTTGACTTGAA	TTGAACTCCTTGATGAATGA

Table S3 RT-qPCR reaction system

Reagents	Volume
2×ChamQ universal SYBR qPCR master mix	10 μL
Forward primer (10 μmol L^{-1})	0.4 μL
Reverse primer (10 μmol L^{-1})	0.4 μL
cDNA	1 μL
Nuclease-free water	8.2 μL

Table S4 Amplification primers of candidate ODE genes

Gene	Forward primer	Reverse primer
PxylCYP9f2	ATGATAACCGAAATTCTCGTATTTATATT	CTACTTTCTCGCGCGGCA
PxylGSTe1	ATGATGCGGAAGCTACTGTTTAGA	TCAGTGTGCTACATCTTTAATTTGATCT
PxylGSTs1	ATGGCCAAGAAACTACACTACTTCG	TTAAAGCGCGTAGACCTTCCTC

Table S5 Amplification of target fragment reaction system

Reagents	Volume
Prime STAR max premix	25 μL
Forward primer (10 μmol L^{-1})	2 μL
Reverse primer (10 μmol L^{-1})	2 μL
Template	1 μL
Nuclease-free water	20 μL

Table S6 Sequences information used to phylogenetic tree analysis to PxylGST

Gene name	Accession number
SlitGSTd1	KF482970
SlitGSTd2	KF482971
SlitGSTd3	KF482972
SlitGSTe1	AAS79891
SlitGSTe2	ACZ73898
SlitGSTe3	ACZ738989
SlitGSTo1	HQ667939
SlitGSTo2	KF482977
SlitGSTo3	KF482978
SlitGSTs1	HQ667936
SlitGSTs2	HQ667937
SlitGSTs3	HQ667938
SlitGSTt1	KF482976
SlitGSTz1	KF482974
SlitGSTz2	KF482975
SlitGSTu1	HQ667940
EsemGSTd1	ACB36909.1
EsemGSTd2	AFD34184.1
EsemGSTd3	AFD34184.1
EsemGSTd4	AIL23531.1
EsemGSTe1	AIL29312.1
EsemGSTe2	XP_022815498.1
EsemGSTe3	AIL29312.1
EsemGSTz1	ARM39005.1
EsemGSTz2	XP_022828689.1
EsemGSTt1	ARM39006.1
EsemGSTs1	XP_023950267.1
EsemGSTs2	AFF27579.1

EsemGSTo1	XP_011561287.1
EsemGSTo2	XP_023946805.1
EsemGSTo3	OWR42990.1
EsemGSTu1	XP_023943781.1
EsemGSTu2	AMY26654.1
BmorGSTe1	NP_001037197.1
BmorGSTe2	NP_001345908.1
BmorGSTe3	NP_001108466.1
BmorGSTe4	NP_001108460.1
BmorGSTd1	NP_001037183.1
BmorGSTd2	NP_001036974.1
BmorGSTd3	NP_001037546.1
BmorGSTo1	NP_001040131.1
BmorGSTo2	NP_001037406.1
BmorGSTo3	NP_001040435.1
BmorGSTo4	NP_001108461.1
BmorGSTs1	NP_001037077.1
BmorGSTs2	NP_001036994.1
BmorGSTt1	NP_001108463.1
BmorGSTu1	NP_001108462.1
BmorGSTz1	NP_001037418.1
BmorGSTz2	NP_001040453.1
SlittoGSTd1	MH177577
SlittoGSTd2	MH177578
SlittoGSTd3	MH177579
SlittoGSTe1	MH177580
SlittoGSTe2	MH177581
SlittoGSTe3	MH177582
SlittoGSTe4	MH177583
SlittoGSTe5	MH177584

SlittoGSTe6	MH177585
SlittoGSTe7	MH177586
SlittoGSTe8	MH177587
SlittoGSTe9	MH177588
SlittoGSTe10	MH177589
SlittoGSTe11	MH177590
SlittoGSTe12	MH177591
SlittoGSTe13	MH177592
SlittoGSTe14	MH177593
SlittoGSTe15	MH177594
SlittoGSTe17	MH177596
SlittoGSTo1	MH177600
SlittoGSTo2	MH177601
SlittoGSTo3	MH177602
SlittoGSTs1	MH177603
SlittoGSTs2	MH177604
SlittoGSTs3	MH177605
SlittoGSTs4	MH177606
SlittoGSTs5	MH177607
SlittoGSTs6	MH177608
SlittoGSTt1	MH177609
SlittoGSTu1	MH177610
SlittoGSTz1	MH177612
SlittoGSTz2	MH177613
CmedGSTd1	KM099178
CmedGSTd2	KM099179
CmedGSTd3	KM433686
CmedGSTe1	KM099180
CmedGSTe2	KM099181
CmedGSTe3	KM099182

CmedGSTe4	KM099183
CmedGSTo1	KM099186
CmedGSTo2	KM099187
CmedGSTo3	KM433691
CmedGSTs1	KM099188
CmedGSTs2	KM099189
CmedGSTz1	KM099191
CmedGSTz2	KM099192
CmedGSTu1	KM099193

Table S7 Sequences information used to phylogenetic tree analysis to PxylCYP

Gene name	Accession number
SlittoCYP6AB14	JX310081
SlittoCYP18A1	CAJ31114.1
SlittoCYP18B3	JQ979045
SlittoCYP304F4	JQ979046
SlittoCYP305B1	JQ979047
SlittoCYP306A1	ACM45975
SlittoCYP6AB31	JX310080
SlittoCYP6AE47	JX310077
SlittoCYP6AE48	JX310078
SlittoCYP6AE49	JX310082
SlittoCYP6AE50	JX310083
SlittoCYP6B42	JX310079
SlittoCYP6B48	JX310076
SlittoCYP9A51	JX310084
SlittoCYP9A52	JQ979048
SlittoCYP321A11	JX310085
SlittoCYP321A12	JQ979049
SlittoCYP324A6	JX310101
SlittoCYP324A1	JX310086
SlittoCYP338A2	JX310075
SlittoCYP354A9	JX310100
SlittoCYP4G75	JX310087
SlittoCYP4L12	JX310088
SlittoCYP4S8v1	JX310089
SlittoCYP340AA1	JX310093
SlittoCYP340AB1	JX310096
SlittoCYP340K4	JX310091
SlittoCYP341A13	JX310094

SlittoCYP341B3	JX310095
SlittoCYP366D1	JX310097
SlittoCYP367A6	JX310090
SlittoCYP367B6	JX310092
SlittoCYP333A6	JX310098
SlittoCYP333B3	JX310099
BmorCYP4M5	EF523434.1
BmorCYP4M9	EF017796
BmorCYP6AB5	NP_001104007.1
BmorCYP6AE9	NP_001104004.1
BmorCYP6AE22	NP_001104005.1
BmorCYP6AU1	EF547546.1
BmorCYP6AB4	EF017798.1
BmorCYP4G25	NP_001106223.1
BmorCYP305B1	AB044900.1
BmorCYP6B29	DQ252324.1
BmorCYP9A19	EF488994.2
BmorCYP9A22	EF535804.2
BmorCYP339A1	EU678355.1
BmorCYP307A1	NM_001111363
BmorCYP6AE21	FJ387169.1
BmorCYP9A20	NP_001077079.1
BmorCYP15C1	AB124839.1
BmorCYP18A1	EF421988.3
BmorCYP9A21	NP_001103404.1
BmorCYP49A1	AK343221.1
BmorCYP314A1	AK289331.1
BmorCYP315A1	AB167737.1
BmorCYP302A1	AB198340.1
BmorCYP333A2	AK289310.1

BmorCYP333B1	AK343202.1
BmorCYP306A1	AB162964.1
BmorCYP6AE2	EF415296.1
BmorCYP9G3	EU435134.1
BmorCYP337A2	AK289340.1
BmorCYP340A2	AK289341.1
HarmCYP6AE12	NP_001428538.1
HarmCYP6AE14	DQ986461.1
HarmCYP6B2	U18085.1
HarmCYP6B6	AY950636
HarmCYP6B7	AF031468
HarmCYP9A12	AY371318
HarmCYP9A14	AY487948
HarmCYP9A17	AY753201
HarmCYP9A18	DQ256408.1
HarmCYP9AJ3	AID54908.1
HarmCYP9G5	KM016757.1
HarmCYP9A3	PZC75620.1
HarmCYP6AB9	AID54886.1
HarmCYP337B1	EF591061
HarmCYP337B3	KM675664
HarmCYP4S1	EF591060.1
HarmCYP321A1v1	OM863791.1
HarmCYP321A1v2	OM863792.1
HarmCYP321A1v3	OM863793.1
HarmCYP321A1v4	OM863794.1
HarmCYP321A1v5	OM863795.1
SfruCYPCY321A8	KC789751
SfruCYP321A9	KC789752
SfruCYP321B1	AGO62009.1

SfruCYP6B50	KC789749
SfruCYP6AB12	KC789747.1
SfruCYP6AE43	KJ671575.1
SfruCYP6AE44	KJ671576
SfruCYP4G75	KC789746
SfruCYP4L9	FP340412.1
SfruCYP9F1	ULR85469.1
SfruCYP337B5	KJ671580
SfruCYP9A32	FP340410.1
SfruCYP6AN4	KC789748
SfruCYP9A58	KJ671577
SfruCYP9A59	KJ671578.1
SfruCYP9A60	KJ671579.1
SfruCYP321A7	KC789750
SfruCYP321A10	KC789753
SfruCYP340L1	AGO62010.1
SfruCYP305A1	MN480675.1
SfruCYP49A1	MN480673.1
SfruCYP4L4	MN480659.1
CsupCYP6SN3	ON693840
CsupCYP306A1	ON693841
CsupCYP4AU10	KF701166
CsupCYP4AU11	KF701167
CsupCYP4G90	KF701159
CsupCYP4G91	KF701160
CsupCYP4L27	KF701162
CsupCYP4M38	KF701163
CsupCYP4M39	KF701164
CsupCYP6AB45	KF701129
CsupCYP6AB48	KF701132

CsupCYP6AB46	KF701130
CsupCYP6AB47	KF701131
CsupCYP6AB49	KF701190
CsupCYP6AB50	KF701133
CsupCYP6AB51	KF701134
CsupCYP6AB52	KF701135
CsupCYP6AB53	KF701136
CsupCYP6AW1	KF701139
CsupCYP6CT1	KF701140
CsupCYP6CV4	KF701141
CsupCYP6CV5	KF701142
CsupCYP9A68	KF701145
CsupCYP9A69	KF701146
CsupCYP18A1	KF701122
CsupCYP301A1	KF701181
CsupCYP307A2	KF701128
CsupCYP321F1	KF701152
CsupCYP321F2	KF701153
CsupCYP321F3	KF701154
CsupCYP338A1	KF701157
CsupCYP341A15	KF701170
CsupCYP367A9	KF701176

Table S8 Link reaction system of recycled products

Reagents	Volume
Recycled product	1.5 μL
10 × Enhancer	1 μL
pESI-Blunt simple vector	1 μL
Nuclease-free water	6.5 μL

Table S9 Reaction system of positive clone validation to single colony

Reagents	Volume
MonAmp™ 2×Trio taq mix	12.5 μL
M13-F	1 μL
M13-R	1 μL
Bacteria colony	1 μL
Nuclease-free water	9.5 μL

Table S10 Prokaryotic expression primers of *P. xylostella* ODEs

Gene	Forward primer	Reverse primer
PxylCYP9f2	GACGACAAGGCCATGGATGATAACC<br>GAAATTCTCGTATTTATATTTACAAC<br>AATAGTTTTT	GGTGGTGGTGCTCGAGTCTTTCTC<br>GCGCGGCAC
PxylGSTe1	AAGGAGATATACATATGATGATGCG<br>GAAGCTACTGTTTAGAAATT	GGTGGTGGTGCTCGAGTGTGTGCT<br>ACATCTTTAATTTGATCTATTAAA<br>AATTGTAATCTATCCA
PxylGSTs1	AAGGAGATATACATATGATGGCCAA<br>GAAACTACACTACTTCGAC	GGTGGTGGTGCTCGAGTAAGCGC<br>GTAGACCTTCCT

Note: Restrictive enzyme cleavage sites were marked with underline

Table S11 Double enzyme digestion reaction system

Reagents	Volume or weight
10×Quick cut green buffer	2 μL
*Nde*I or *Nco*I	2 μL
*Xho*I	2 μL
Recombination plasmid	2 μg
Nuclease-free water	To 20 μL

Table S12 Homologous recombination reaction system

Reagents	Volume or weight
MonClone™ single assembly cloning mix	5 μL
Linearized vector	108 ng
Fragment	20 ng
Nuclease-free water	To 10 μL

Table S13 PCR reaction system of recombination product colony

Reagents	Volume
MonAmp™ 2×Trio taq mix	12.5 μL
T7	1 μL
T7t	1 μL
Template	1 μL
Nuclease-free water	9.5 μL

Table S14 Site directed mutagenesis primer design

Gene	Forward primer	Reverse primer
PxylGSTs1^{Y7A}	CACGCCTTCGACGTTAACGGCATTG GCGAG	TTAACGTCGAAGGCGTGTAGTTTC TTGGCCAT

Table S15 Site directed mutagenesis PCR amplification reaction system

Reagents	Volume or weight
2×Max buffer	25 μL
dNTP Mix (10 mmol L^{-1} each)	1 μL
Template DNA	1 ng
Forward primer	2 μL
Reverse primer	2 μL
Phanta Max Super-Fidelity DNA Polymerase	1 μL
Nuclease-free water	To 50 μL

Table S16 Digestion reaction system

Reagents	Volume
Dpn I	1 μL
PCR amplification product	40 μL

Table S17 Recombination reaction system

Reagents	Volume or weight
Dpn I digestion product	110 ng
5×CE II Buffer	4 μL
Exnase II	2 μL
Nuclease-free water	To 20 μL

Table S18 Binding energy of 2,3-dimethyl-6-(1-hydroxy)-pyrazine between four PxylGSTS1 mutation sites

Ligand name	Binding energy (kcal mol^{-1})	Ligand energy (kcal mol^{-1})	Protein energy (kcal mol^{-1})	Complex energy (kcal mol^{-1})	Entropic energy (kcal mol^{-1})
PxylGSTS1-Y7A	-5.6	38.4	-17454.6	-17421.8	17.5
PxylGSTS1-F8A	-20.9	37.5	-17903.5	-17886.8	17.5
PxylGSTS1-I35A	-22.2	37.2	-17818.6	-17803.7	17.5
PxylGSTS1-W100A	-4.3	39.6	-16650.9	-16615.5	17.5

File S1

Sequences used to phylogenetic tree analysis to PxylGSTs

>BmorGSTe1

MVLTLYKMDASPPVRAVYMVIEALSIPNVKYVDVDLLAEDHLKEEFLKLNPQHTIPMLTD

DKFVIWDSHAIATYLLNKYGKGSSYYPEDPEKRALIEMRLHFDSGILYPALRENDEPIFFW

GETTFKPEGLAKIKSAYDFTEKFLSDSPWIAGDDVTVADMSCVATIGSLDALLPINEKEYPK

ITSWLKRCSELDFYQRGNNVKGLLEFKALLKQYLSRGKE

>BmorGSTe2

MSIIIYQTSVSPPARSALMVVNILGIKAETREVTLPRRDHYSQEYLEKNPLHTVPILEEDDLV

IADSHAIITYLVSKYGEEKHESMYPKDLKIRAITDQMLYFDATILFPRLKTVIYSVVRGQGM

SRQQIADIQEAYDVLEIYLSKNIFVAGNEFTVADISCVATLSSLDCVLPVDKKHVNVNRWW

ATLSNEKWYKEVNVPGLELFRSFIKQFLK

>BmorGSTe3

MSLMLYKLNASPPARTAMMVCELFKVPVKMVDVNLSKGEHFSPEYLKRNPLHTVPTLED

GDLIITDSHAIAMYLADKYGKDDSLYPKDLKSRAIVNQRLFFDSTVLFSRMRSVTFPVIIEG

CKTVTEKQINDIIEAYGYVETYLSNTKFIATNNLTIADISAYAVVSSLLFIVPLDGAKFPKTQT

WLNEMEKKPFAQKYNVNGVAELGALLKEKLGS

>BmorGSTe4

MVFILYKKDTSPPCRSVQMVLHELGIYDVELIEVNLPERDHLKEEFLRMNPQHTVPTLIDG

DFIIWDSHAIVTYLVNRYAKNDTLYPKEPKQRAIVDQRLHFDTGVLFAILRATAEPVLYNNE

KSFKQENLEKMEAAYEFVEKFLTSDWLAGDQVTLADICCVSTISSMNVIVPIDKKKYPKIIS

WLQRCSEQEFYKKANEPGLKKFIEMFKNKIGN

>BmorGSTd1

MPVQPIKLYYLPPSPPCRAVMMTARVLELDLHLITTNIMNGEHMTPEYLKMNPQHTIPTM

DDNGFILWESRAIQTYLVNAYGKDDSLYPKNPRQRAIIDQRLNFDLGTLYLRYLNLYTPILF

RGEAYDQEKADKFDEALGWLNTFLDGRPFVAGENMTVADITIVVTITNIDAFGYDFSSHE

NIAKWFERTKKMLEPYGYDEIDVTGAKMLASFLKKE

>BmorGSTd2

MTIDLYYVPGSAPCRAVLLTAKALNLNLNLKLVDLHHGEQLKPEYLKLNPQHTVPTLVDD

GLSIWESRAIITYLVNKYAKGSSLYPEDPKARALVDQRLYFDIGTLYQRFSDYFYPQVFAGA

PADKAKNEKVQEALQLLDKFLEGQKYVAGPNLTVADLSLIASVSSLEASDIDFKKYANVK
RWYETVKSTAPGYQEANEKGLEAFKGLVNSMLKK

>BmorGSTd3

MAIDLYFTAGSAPCRVVLLVAAALDLQLNLKPLNLWEREQLQADFLKLNPQHTVPTIVDE
GFPLWESRAISRYLVNKYGGDSSSLYPKDLMARALVDQRLDFDIGTLYPRFAQYFYPQVFG
GAKPDAAALKKLEEALVFLNAFLEGQKYVTGDVLTIADLSLVATISTIDAAEISLKSYPNVE
KWFELMKTTAPDYQNANQKGIDEFKKLIAQMKAKTEL

>BmorGSTo1

MSEKHLQTGDVLPPYSGKLRVFAMRFCPYAERTVLTLNAKNIPYDLVFINLDQKPEWIFNF
SPKGTVPALEYEPGKALFDSNIINVYLDEKYPEIPLQASDPLRRAQDKILVESFAPAQSAYYT
AAFNAQALEPSMVETYHKGLEGLQKELETRSTKYLHGDEPGWVDYTLWPFLERFEALPLI
GKAEFAIDQTKYERLVTYIEAMKNVPAVKSYFLAAETHAKFIESRAQGDANYNMLDTSAV
CCMRPRKKKE

>BmorGSTo2

MSAIKDSRNINFNIKHLRKGDPLPPFNGKLRVYNMRYCPYAQRTILALNAKQIDYEVVNID
LIDKPEWLTTKSAFAKVPAIEIAEDVTIYESLVTVEYLDEVYPKRPLLPQDPLKKALDKIIVE
ASAPIQSLFIKILKFSDTVNEEHVAAYHKALDFIQEQLKNRGTVFLDGSEPGYADYMIWPW
FERLRAFAHDERVRLEPSKYSLLLEYIDNMLKDSAVSQYLIPLEILAKFHEAYTKKERPNYE
LLNECLKSF

>BmorGSTo3

MTYFHSVNAGVIPPPALTDKLRLYHVDMNPYGHRVLLVLEAKRIKYEVYRLDPLRLPEWF
RAKNPRLKIPVLEIPTDQGDRFLFESVVICDYLDEKYTRHTLHSHDPYVKAQDRLLIERFN
ELIKGSLECFDTNFAFGSEQIIQTLEIFEKELTNRGTNYFGGNRPGMLDYMVWPWVERLYL
LRCVNDRKFVEKKSLFPNFADWGDQMQLDDIVKKHAHSPQEYFDYYKNARAHSMGYY
L

>BmorGSTo4

MVSPKINFNTKHLGKGDPLPPWSGKLRVYNMRLCPFAQRTILTLNAKQIDYEVINIDLVNK
PEWLPTKSIFGKVPTIEVEDGVCICESLIIAEYLEEVYPEIPLISKDPIKKAYEKIIIEASEPIFV
MYFKVMRTPDTINDETLMSYHKALTFFEGQLRNRGTRFLGGEKPGFADYMIWPWFERIQS
MNDEKLKIKSAKFDLLVAYIENMYKDPAVSQYLLPKDVMDKLHAEYKTGKFEVQSIEDLL

>BmorGSTs1

MPKVVYHYFACKALGESGRMLLAYGGQDFEDHRVLSADWPDFKPKTPFGQTPVLVIDGK

QYAQSTAICRYLGRKYGLAGANDEEAFEIDQNVEFLHDIRAKAAAVYYEADEELKAKKH

EDFSKNVYPDMLKKLNSIVEANKGHIAAGKLTWGDFVFTSMFDYLKTMLQIPDLEVQYP

AFKKVLQSVLTQPKVKAFLDLGRPYEFEF

>BmorGSTs2

MPNVKFYYFPVKALGESQRLLLAYGGQEFEDNRISSENWPEFKPKTPFGQMPVLEIDGKQ

YAQSTAICRYLGRKYGLAGANDEEAFEIDQNVEFLNDIRASAASVHYEKDEAVKAKKKAE

LEETKYPFFFEKLNEILTKNNGHIALGKLTWGDFVYAGMYDYLKAMLQKPDLEQKYPAF

RKPIEAVLAIPKVKAYVDAAPRTEL

>BmorGSTt1

MVLKLYYDLMSQPSRVLYILLKTMKYDFEPKYVNLRKAEHYSEDFTKVNRMQRVPVIDH

NGFILTESIAILKYLSRENVIAESLYSKESKLQARIEEFLEWQHIGLRLHCAMYFRVVHMDPI

LTGRKSDEKTIQGYKRRMMMALDDFDTKWLGRGTAFIVGETPTVADLVAACELEQPRMA

GFEPKDHFPNIAAWWPKVRDHFAPHYEDAHVILNKIINKMDRAANSKL

>BmorGSTz1

MGKQPVLYSYWRSSCSWRVRIALNLKEIPYDIKAVSLIKGGGEQHCNEYREVNPMEQVPS

LCIDGHTLIESLNIMHYLEETRPQRPLMPQDCFKRAKVREICEMIASGIQPLQNLIVLIYVG

EEKKKEWSQHWITRGFRAIEKLLSTTAGKYCVGDEITLADCCLVPQVFNARRFHVDLRPF

PIILRIDRELENHPAFRAAHPSSQPDCPPEVAK

>BmorGSTz2

MVENRVILHAYWLSSCSWRVRAMLHAKSIPFEERPVDIVKTGKQLTEEYRAINPAQKVPA

LEIDGVTLVESTAIIQYIEDTRPEPKLMPDTALQRARMREICETIVSGIQPLQNFGLKKHLGT

EEKFLSFTKYWTERGLQTLNDLLAKTSGAYCIGDQITLADICLVPQIYNGVSRHKLDLKTY

PIVSKVYENLLKEELYQATHPKATKEKLKINL

>BmorGSTu1

MVLKLYAVSDGPPSLSVRQALVALEVPFELINVDFGAGEHMTSDYALMNPQKEIPVLDDE

GFYLSESNAILQYICDKYRPGSPLYPQDPKSRAIVNHRLCFNLSSYYANISAYTMAPIFFDY

ERTPLGLKKVHISLDVLETYLTRTNTSYAAANHLTIADFPLINSTMTLEAIDFDFSKYTKIHK

WYNDFKVKYPDLWKISESAMKEIQHFAANPPDLTHLNHPIHPIRKIKN

>SlitGSTd1

MALALYYTAGSAPCRLVLLVAAALNIQLDLNTVDLRAGEQFNPEFLKLNPQHTVPTLVDG

EFSLWESRAISRYLVSKYGDENNSLYPSDPQARAVVDQRLDFDLGTLYPRFGGYFYPQIFG

GAKADEALLKKLEEALVFLNTFLEGNTYAAGDKLTLADLSLVATVSTIDATGIVSLDKYPN

VQKWFELVKTSAPG

>SlitGSTd2

MAAKVIGVDLDLVLTNIMEGHHMTPEYLKMNPQHTIPTMDDSGFILWESRAILAYLANAY

SRDDTLYPKNPRQRAIVDQRLNFDLGTLYVRYSALYLPMLFRGEEYDEQKADQLDEALG

WLNTFLDGRAFVAGDNLTIADISIIVTITNLEAFGYDMSGHPNL

>SlitGSTd3

MSVKLYHFPISGPSRGALLAARAVGAPVEVEIINLFQKEQLKESFLKINPQHCIPTLDDTGFI

LWESRAIAGYLVDKYGKDDHLYPKDLKKRALVNQRLYFDSSSLYVKIRAICFPILFLGETEI

KKPLKDDLNVTLGFLDQFLKDSKWVAGDQPTIADTSIYASVSSILAVGWDISGFPSIVRWV

GQCSSLPGAAENDEGAKAFGDAVKKNLH

>SlitGSTe1

MPLKIYKLDASPPARAVMMLAELLKLKHEAVDVNLMTGDHLTPEYLKKNPQHTVPLLED

GDFYVADSHAINTYLASKYGGAQSAQLYPTDLQVRATIDSRLYFDISAIAGNSGAIVSALL

RGDITSPTKEQTDKLNSAYEILDKFLQKTKFVAADHLTVADISLAASVSSASLLVPIDEKYS

KLTAWFNTIQQEDWYKKANVPGLEGYKAFVQSKLK

>SlitGSTe2

MAPILYKIDGSPPANAVRILSDIIGLELEVRDVNFGVLEHKSPEHLKRNPMGTVPTLIDGDF

TISESHATMKYLLSVYGGDKSESLYPSDVRTRAIVDQCIFFNVGIFFIRLKVVVLPAIFGDLD

GPTEQHKADIDEAYGIVEAYLSKNKYIAADHLTIADLSVGATAISMQPLHKLDAAKFPLTA

AWMDKLKEHPSVQKYLVPGAKGLGEIVHAAWERNKKK

>SlitGSTe3

MLAASPPACAVRMVAHIIGLKLDYKEPDITKMEHKSPEYLKLNPLGTIPVLIDDDFILSDSH

AIMIYLLSKYGGEHGERLYPSDIRTRAVVNQVMFFDTGILFVRIKVIALPTIMEGMKAPTQK

HLNDLEEAYGMVEAYASRYKYIAADHLTIADLSLAMTMGAAQVLHKLNHKKFPNTLNW

LSLVEKEPFFIQIGLPGIAKFNTAMQYYTARNNQ

>SlitGSTo1

MSEKHLQTGDALPPYGGKLRLFAMRFCPYAERTVLVMNAKNLQYDTVFVNLDHKPEWL
LNYSPKGTVPALEYEEGKGIFDSNIINFYLDEKYPDVPLQSTDPLRRAEDKIIVELFAGVQS
AFYTAAYNSQALQPNMVDTYHKGLVALQDTLRARGTKFLHGETPGLVDFTLFPFLERFEA
LPLVGKTEFELVKSKYELLVNYIEAMKIVPAVKLYYLSPETHAKFIESRNKGDANYNMLDT
SGVCCMRPRKKKE

>SlitGSTo2

MLSSTISFAVHLYSSLQGVQTIGRALGSLLPYSTMVGSNKRNFDTPHLKTGDPLPPYTGKL
RLYNMRYCPFAQRTVLALNAKNIDYEIVNIDLMDKPEWLTTKSAFGKVPALEIAENVSIYE
SLVTVEYLDDVYPQRPLLPKDPVKKAVDKILVEALSAIHAVYFKLVRGPDTITEDNITAYYK
NLTFIQQELQNRGTKFLDGDQPGYADYMIWPWFERLHVINHDLARLDKQKYKLLVEYID
NMFQDPVVAEYKLPREALEHFQNQYKLGKKPVYDYLIQK

>SlitGSTo3

MSFYQQRGVGPLPPPALTDKLRLYHVDMNPYGHRVLLILEAKKAKYEVYKLDPLRLPEW
FRNKNPRLKIPVLEIPTEYGDKYLFESVVICDYLDEKYTRNQLHSRDPYVKAQDRLLIERF
NELIKGSLECFDTNFAFGSEQIIQTLDIFEKELTLRGTNYFGGHRPGMLDYMIWPWVERLY
LLRCVNERKFDEKRGLFPNFADWSDQMQLDDVVKKHANSPQEYFDYYKNARTHSMGY
YL

>SlitGSTs1

MPKYVFHYFPIKALGESVRLLLAYGGEGFEDHRIDLDDWPKFKPNTPFGQMPVIEFDGKQ
YAQSIAIARYLGNKYGLAGDTLEDNLEIDQNVYLINDLRIKAASAHYEKDDVIKEQKYKE
FSKGVFPDSLEMLNALFAKNNGHVALGKLTWADFMFAGLFDYLSTMMRMPDLGQKYPA
LQQVKDRVYSFPKVKAYVDAVPA

>SlitGSTs2

MPKYVFHYFDGKGLGEPVRLLLAFGDEGFEDHRVAFKDWPDFKPKTPFGQMPLLEFEGK
QYAQSLSIARYLGKKYGLAGESLEDALEIDQNVDLINDLRAKAAIASYEKDEAVKEKKYA
EFNKDVFPNMLEKLNEIITKNNGHIAIGKLSWGDFVFAGMFDYIKHLLVVPDLEKKYPAFQ
KVVDAVYSIPKVKTYADAVGPTEF

>SlitGSTs3

MPKYVVHYFNGKGLGEPIRLLLAYGGEEFEDHRVAGKDWPEYKKNTVFGQMPNVDIDG
KKYAQSIAICRYLGNKYGLAGDTPEEALEIDQLVDLLVDFRLKAATVTYEKDEKLKEQRY

EEYSKDVFPEHLDRFNDIIEKNNGHYALGKLTWADFVFAGFFDSMKFIVRIPDLEKKYPAF
QRVIDHVYSIPKVKAYADALGPTEF

>SlitGSTt1

MSLKLYCDLMSQPSRALYILLKSIKCNFEPKFVNLRQAEHYTEEYSAINRFQKVPVIDHNG
FILTESVAILKYLSRENVIPESLYPKESKAQARVEEFLEWQHAGLRLHCAMYFRVKYLNPV
MFGKPADPVSLAGYESRMEAALTEFNDKWLGRGTDFINGNTPTVADLLAACELEQPRMS
GYDPCANFKNIQVWWKKVREHFNPYYDEGHVIVNKIIKKNTPVASKI

>SlitGSTz1

MGDDVETKPVLYSYWRSSCSWRVRIALNLKEIPYDIKAVSLIKGGGEQHCNEYREVNPME
QVPSLCIDGHTLVESLNIMHYLEETRPNQRPLMPQDCFKRAKVREICEVISSGIQPLQNLIV
LIYVGEEKKKEWAQHWITRGFRAVEKLLSACAGKYCVGDEITLADCCLVPQVFNARRFH
VDLRPFPIILRIDRELENHPAFRAAHPSSQPDCPPEVAK

>SlitGSTz2

MAENRAILYAYWLSSCSWRVRAALHFKGIPFEERPIDIVKTNQQKTEQFRAINPAQKVPAL
VIDNVTLVESMAIVQYLEDTHPEPTLTPKTPVLRARMRELCEVVVSGIQPLQNIGLRSQFD
TTEQYTKFTKYWTDRGLMTLEDLLQKSAGKYCVGDQLTLADLCLVPQLYNAVTRHALDI
SKYPTVSKLYESLLKENVFKETHPESVKSKM

>SlitGSTu1

MVLKLYYLEDGPPSLSCRQTLEALGVPFEIVNVSFYHGEHMTEEFAKKNPQKELPVLEDD
GFFLSESVAMMQYICDKFKPNSSLYPTDSKARAIINHRLMFTMTTYYAELLNYFIMPVYFK
YERTPEALKRVHRALDLFETYLERENTSYSAANHLTIADFPLINATMAMEVTDFDFSKYKR
ITKWYNDFKKNQPKLWSITAEGMRLMRKCVENPPDLSHLNHPIHPARKAE

>CmedGSTd1

MKAIALVVAILISINNLGNAVARSKSKMPTQAIKLYYLPPSPPCRAVMMAAKVLGVELDLV
VTNIMEGEHLRPEFMKMNPQHTIPTMDDSGFILWESRAIMTYMANAYGKDDSLYPKNPR
QRAVVDQRLNFDLGTLFMRYINLYGPLIFKGGSMDEEKAAKLNEAIGWMNTMLEGKAFV
AGDNMTLADISIIVTYTCLEAFEYDFSQYENVMKWFERMKKELQPYGYEEIDRAGAQML
ATFLKSHAN

>CmedGSTd2

MGIDLYYVPGSPPCRAVLLTARALNLNLNLKLVDMNSGEHLKPEFLKLNPQHTVPTLVDD

GHSVWESRAIITYLANKYGKGKPLYPEDPKERSQVDQALYFNIGTLYHRWADYFFPQVFG
GAPADKAKLEKVEEALKMLDTMLEGHKFITGNNMTIADLDYLASISSFEASDVNFLKYPN
IKRWYETAKSSAPGYEEANAAGLVALKELCKTLMKK

>CmedGSTd3

MASCEVRSNKAKIDLYYRPGSPPCRAVRLVAAALGIDLNLKLVDLHGGEHLKPEYLKLNP
QHTIPTMVDEGFSLWESRAIMAYLVNKYGKNDSLYPKDPKARALVDQRLYFDFGTLSMSF
TDYFYPMLFAGAPEDKSKYDKIAVGFDLLEIFLEGQKYVAGSQLTIADLALIVTVSNFELIK
YDISKYKNVSRWATKIKEEATGYADCNGEGLKAFQELIDHLMKK

>CmedGSTe1

MVRPILYFGIWSPPSSAVRMTAKAIGLDLNLREIDLFKKDHLKDDYLKINPQHTIPVFNDH
GFVISDSHAIMTYMVDKYAENDNLYPKDLKKRAIVNQRLHYSGGVIFCRLLGALQPLLRG
TTKTVSPASQQFLEDSYKYINTILDGRKWLAGDSYTLADISTITAISSVTVFKSINDYPNLIN
WMKNCQELPGYPDIAAVGIKEFHNLVSQRLEK

>CmedGSTe2

MRGEKTVLYKNDQSPPARSVKMLMSLLGLNFIEQRDINPLFREQDTPEMRKKNPMRTIPFI
DDGDFSLGDSHAICLYLLEKYGKPEHEYLYPKDIKKRATVNQRLFFDCGVVFQRLRAVMA
PTYMGQMTEMPKEVIGGIRNAYGTLEAYLSESVYLADDVMTIADICTISTVSSLDGLHPVD
GKRYPKLRQWLTNMYAKDFVKEENQQGCEDHVTYLFAAMELNKQQKSKL

>CmedGSTe3

MVLTLYKLDGSPPARAVMMTIEALGIPDVEFIDVNLLTAEHMTEEFLKINPQHTIPMLKDD
DFIIWDSHAINAYLVNKYGENDALYPTDFKKRAQIDQRLHFDSGILFPALRGTLEPVVFGD
EKSFRPESLNKIRAAYDFTEKFLTSPYLLGDDVTLADICCVATISSMNLLVPIDDNTYPNVSA
WIQRCASKEFYKKANEPGLEQLTQLIKTKCE

>CmedGSTe4

MPAKLYKMDLSPPARAAMLACEIFNVPVELVDTNLMAGEHLKPEFLKMNPCHTIPVLVDG
DITLHDSHAIIMYLADTYGNNSALYPKDVKQRALVNQKLFFNSSVLFTRLRNVTIPAFKEG
VKGLDDKKRTDIEEAYKYLEAFLSGTKYLAGNTITIADIAALTTIGGLSYIVPLDGDKYPKL
KAWFEELENKPYSQKVNAGGAKALGEFIKSCIQG

>CmedGSTo1

MSEKHLQAGDVLPPYNGKLRLFAMRFCPYAERTVLVLNAKGLEYDLVFVNLDHKPDWLF

NFSPKGTVPALEYEQGKAIFDSNVINVYLDEKYPEKPLQAADPLRRAQDKLIVENFAAAQ
SAYYTAAFNSQALQPSNIENYHKGLEGLQKELEVRGTKFLHGDDAGLVDLTIWPFLERFL
ALPLLGKTEFAIDPSKYSLLNTYIEAMKNVPAVKSYCLAAETHAKFTESRAKGDPDYNML
DTSAVCCMRPRKKKE
>CmedGSTo2
MIGTTVSIAAHVLTTSFRLLSPVTEVLRYVLPSLSYFKTMSSKINFNTKHLGKGDPLPPWTG
KVRVYNMRYCPFAQRTILALNAKQIDYEVVNIDLMNKPDWLFAKSGLGKVPSVEIEENVC
IYESLITVEYIDEVVPGRPLLPKDPARKAMDKIIVEAMTPVTQLFYKCILSPEAITDDLTAAY
FKALDFLQEQLKSRGTKFLSGSEPAYVDYMIWPWFERILIMDEMDGKMTISPEKYQLLLE
YFAHMFKDPAVSQYLVPKKVLQKFFAAYMKREPINYDGIFED
>CmedGSTo3
MTYYEHRTAGIAPPPALTNQLRLYHVDMNPYGHRVLLILEAKRVKYEVYRLDPLRLPEWF
RAKNPRLKIPVLEIPTDHGDKYLFESVVICDYLDEKYPRNPLHATDPYVKAQDRLLIERFN
ELIKGSLECFDTNFAFGSEQIIQTLDIFEKELALRGTNYFGGNRPGMLDYMVWPWVERLYL
LRCLNERKFDEKRSLFPNFADWGDQMQLEDVVKKHSNSPAEYFEYYKNARAHTMGYYL
>CmedGSTs1
MPSYKLTYFPVKALAEPIRFILSYGGVEFEDDRFDRNDWPKIKPTMPFGQVPVLEIDGKKV
NQSTAICRYLAKQHGLAGKDDWENLEIDVAVDNIHDLRSKIGAYHYESHPEAKEAKREPL
MKELLPFALDRLDEQVKKNGGYFVGGKLTWADMVFVALLDYLNFMTKFDITEKHENLK
ALKEKVLALPKIKAWVEKRPQTEC
>CmedGSTs2
MPTYKLWYFNLTGLGEPIRLMLSYNNIPFEDKRIEFADWPKYKDQMPLGQMPVLEIDGKK
YNQSKAVMRCLAKKFGLYGSSDEEAWEIDATADAIDDLRIPLSTYHWEENPDFKAKLKEP
AFKKKEMLMAKFEEQVKKNGGFFVNKKLSWADLLYAGYTDYLSNVLGHDLNKDHPELK
KLVDKIRAHPGVKAYLDKRPKTSL
>CmedGSTz1
MPVLYSYWRSSCSWRVRIALNLKEIPYDIKAVSLIKGGGEQHCNEYREVNPMEQVPSLVID
GHTLVESLSIMHYLEETRPQRPLMPQDCMKRAKVREICEVISSGIQPLQNLIVLIYVGEEKK
KEWSQHWINRGFRAVEKLLSSSAGKYCVGDEITLADCCLVPQVFNARRFHVDLRPFPIILR
IDRELENHPAFRAAHPSAQPDCPPEVAK

>CmedGSTz2

MAEKAILYGYRFSSCSWRVRAALLAKRIPFEERAVDIVRGREQLTAQYRAINPAQKVPALVI

DGVTLVESIAIVQYLEDTRPLPALTPKSPLDKARMTEICQTIVSGIQPLQNIGLKNFLTSDEE

YTRFSLHWCERGLETLEELLGRTAGTHCVGDQLTVADLCLVPQLFNLTHRFKMDISKHPT

VTRLNTQLLAQDLFQRSHPNTLKKEKDAQT

>CmedGSTu1

MVLKLYGVSDGPPSLSVRQALTQLGVPFELIDVDFGAGDHMTADYAKMNPQKEIPVLDD

DGFFLSESNAILQYVCDKYKPSSPLYPQEPKARALVNHRLCFNLSTYYAHISAYTMAPIFFD

YERTALGLKKVHIALDVFETSLERLGPSHAAANHLTIADFPLINSTMTLEAIDFDFSKYTRI

YKWYNSFKQEYPVLWKISDGAMKEIKHFAANPPDLTHMDHPIHPIRKIIK

>EsemGSTd1

MTIDLYYVPGSAPCRAVLLTAKALNLSLNLKLVDLHHGEHLKPEFLKLNPQHTVPTLVDD

GFSIWESRAIITYLVNKYGKGSSLYPEEPKARALVDNRLYFDIGTLYQRFGDYFYPQVFAG

VPADKEKLTKIEDALQLLDKYLEGQKYAAGPNLTIADLSLVASISSFEASDIDFKKYANVKR

WYETVRSTAPGYQEANEKGLEAFKSLVNSMLKK

>EsemGSTd2

MKGLILTIVFVANLACNAAARSKTSKMPADRIKLYHLPPSPPCRAVRMLAMALNLELELV

MTNLMEGEHLKPEFLKMNPQHTLPTIDDNGFILWESRAIMAYLVNAYGRDDSLYPKNPRL

RAVVDQRLNFDVGTLYARYIAYYAPVLFFGQEKDEEKQKKLDEAIGWFNSMLEGRTFSAG

DNLTIADITIIVTFSTLEALEYDFSEYENVQKWYERTKKALEPYGYKEIDEAGAQMLGSFV

KKD

>EsemGSTd3

MKGLILTIVFVANLACNAAARSKTSKMPADRIKLYHLPPSPPCRAVRMLAMALNLELELV

MTNLMEGEHLKPEFLKMNPQHTLPTIDDNGFILWESRAIMAYLVNAYGRDDSLYPKNPRL

RAVVDQRLNFDVGTLYARYIAYYAPVLFFGQEKDEEKQKKLDEAIGWFNSMLEGRTFSAG

DNLTIADITIIVTFSTLEALEYDFSEYENVQKWYERTKKALEPYGYKEIDEAGAQMLGSFV

KKD

>EsemGSTd4

MPIDLYYLPGSAPCRAVLLAAKAVGVDLNLKLTDLMKGEHLTPEFIKINPQHTIPTLVDNGF

ALWESRAIMTYLADQYGKNDSLYPKDPKKRALVDQRLYFDIGTLYARFADYYYPVVFGG

ADYDPAKLEKIKDAFQFFDVFLKDQEFVSGNQLTLADLSLLATVTTFEAVNFDLSPYKNVT

SWLTRAKAAAPDYEEANGKGVIIFQQMVENLTKK

>EsemGSTe1

MVLTLYKLDGSPPARAVMMTIEALGIPDVEFIDVNLLTAEHMTEEFLKINPQHTIPMLKDD

DFIIWDSHAINAYLVNKYGENDALYPTDFKKRAQIDQRLHFDSGILFPALRGTLEPVVFGD

EKSFRPESLNKIRAAYDFTEKFLTSPYLLGDDVTLADICCVATISSMNLLVPIDDNTYPNVSA

WIQRCASKEFYKKANEPGLEQLTQLIKTKCE

>EsemGSTe2

MGMKVYKRDTSGPCRSVFMVLELLGIKDVEYIQMNLIQREHFSEEYLKMNPQHTIPTLKD

GDFVIWDSHAIVTYLVNQYAKDDSLYPKDPKKRAIVDQRLHFDNGVLFAALKTTMVPILY

NGETAFRQENLDKIKEGYAFIEKFFSGPWLAGESITLADICCVSNISSLNEIFPIDKALYPKLS

AWFERCSKQDFYIKKNLPGLQEFQELLKVKIVS

>EsemGSTe3

MVLTLYKLDGSPPARAVMMTIEALGIPDVEFIDVNLLTAEHMTEEFLKINPQHTIPMLKDD

DFIIWDSHAINAYLVNKYGENDALYPTDFKKRAQIDQRLHFDSGILFPALRGTLEPVVFGD

EKSFRPESLNKIRAAYDFTEKFLTSPYLLGDDVTLADICCVATISSMNLLVPIDDNTYPNVSA

WIQRCASKEFYKKANEPGLEQLTQLIKTKCE

>EsemGSTz1

MAKPVLYSYWRSSCSWRVRIALNLKEIPYDIKAVSLIKGGGEQHCNEYREVNPMEQVPSL

CIDGHTLVESLSIMHYLEETRPQRPLMPQDCFKRAKVREICEVISSGIQPLQNLIVLIYVGEE

KKKEWAGHWITRGFRAVEKLLSASAGKYCVGDEITLADCCLVPQVFNARRFHVDLRPFPI

ILRIDRELENHPAFRAAHPSSQPDCPPEVAK

>EsemGSTz2

MAENRAILYAYWLSSCSWRVRAALHFKGIPFEERPIDIVKTNQQKTEQFRAINPAQKVPAL

VIDNVTLVESMAIVQYLEDTHPEPTLTPKTPVLRARMRELCEVVVSGIQPLQNIGLRSQFD

TTEQYTKFTKYWTDRGLMTLEDLLQKSAGKYCVGDQLTLADLCLVPQLYNAVTRHALDI

SKYPTVSKLYESLLKENVFKETHPESVKSKM

>EsemGSTt1

MTLKLYFDLMSQPSRALYILLKNIKCNFTPVPVDLREAEHYSEEYTKINRFQRVPVIDHNG

FVLTESVAILKYLSREGIIPDSLYPKDSKQAARVEEFLEWQHAGLRLHCAMFFRVKALDPII

TGKTPDPKTLQGYERRMENALGTFNDLWLGQGKPFVAGDHISVADLLAACEVEQPRMAG
YDAIAKYPNIAEWMGRVRDHFNPHYDEGHVILNKIVRNKSKMAPKL
>EsemGSTs1
MPKTTVYYFPLKALGEGIRLLLAYGGEEFEDRRVAKEEWPEMKPKMPFGQMPVLEIDGK
KYAQSSAVVRYLGRKYGLGGKDIEEDFEIDQSVEFYTDIRTKAGAVFHEQNEEAKARMQA
ELEKNYYPFALKKLDEIIKKNNGHLALGKLTWADFLFAGMYDCFKKILQVSDLDEKYPSF
KKLQDTVYAIPKVKAFADKAPKAEY
>EsemGSTs2
MSKYVFYYFDGKGLGEPVRLLLAYGDEGFKDHRVAFKDWPAFKPNTPFGQMPLLEFDGK
KYAQSLSIARYLGKKYGLAGDSLEDALEIDQNVDLINDLRAKAAIASYEKDEAVKEKKYA
EFNKDVFPAMLENLNKIITKNNGHVALGKLTWGDFVFAGMFDYLKHLLVVPDLEKKYPA
FQKVVDNVYSIPKVKTYADAVGPTEF
>EsemGSTo1
MSTRGIKFNTKHLRKGDPLPPYNGKLRLYNMRYCPFAQRTVLALNAKNIDYEVININLFD
KPAWLTSKSAFGKVPSLEIKEGLSIYESLVTVEYIDEVYPQRPLLSKDPVQRALDKIIVEACT
PIQNLYIKLIRFPKTFTEDTVTAYNKALEFLQEQLKIRGTKFFGGAEPGFVDYMIWPWFEQ
VLSYQKIDSRAKIDAEKFKTLLDYLQHLKQDPVVKQYLIEEDVLFKFLEPYKTGGEPNYD
LMLDA
>EsemGSTo2
MITSTLSFAMHILVGSYRVLAPLGQLLEFVLPYRTMAKAVAGKIDFNTKHLKRGDPLPPYN
GKLRVYNMRLCPFAQRTILALNAKNIDYEVVNINLAEKPEWLTSKSAFAKVPSIEIKEGVCI
TESLITVEYLDDAYPEKPLLPKDPVRKALDKIIVEATGPIVTLFIKSVKAPDTITEETISSFYK
ALDYIQDKLKERGTQYLGGDAPGYVDYMIWPWFERLLVLNKDDARASIDPKKFELLMAY
LDKMKRDPAIVPYLVPDETMIAFHEAFKTGSYPNYDVLDLNTK
>EsemGSTo3
MAYYPHRTAGPTPPPPLTDKLRLYHVDMNPYGHRVLLILEAKRAKYEVYRLDPLNLPQWF
KTKNPRLKIPVLEIPTDQGDKYLFESVVICDYLDEKYPRNPLHSRDPYVKAQDRLLIERFN
ELIKGSLECFDTNFAFGGEQIFQTLDIFEKELASRGTNYFGGHSPGMLDYMIWPWVERLYL
LRCVNERKFDEKRTLFPNFADWGDQMQLDDVVKRHSNSPSEYFAYYRNARTHSMGYYL
>EsemGSTu1

MVLKLYGVSDGPPSISVRQALTVLEIPFELINVDYGAGEHMTAEYAKMNPQKEIPVLDDD
GFFLGESNAILQYICDKYKPESNLYPQEPKARAIVNHRLCFNLSTYYANISAYTMAPVFYD
YERTDLGLKKVHMALDVLETYFERLGTSYAAANHLTIADFPLINTTMTMEAIDFDFSKYK
KVSKWYSDFKNNHPELWKISEGAMKEVQYFVKNPPDLTHLNHPIHPARKIAK
>EsemGSTu2
MTPKLYHFPISGPSRGALLAARAVGIPIQIEIIDLFKKEQLKESFLKINPQHCVPTLEDDGFIL
WESRAIACYLADKFGKDDQLYPKDLKQRAIVNQRLYFDSSLLYIKIRAICFPILFLGETEIKQ
GVKDDLNITLGFLDEFLKGGKWVAGDHITIADTSIYASMSSILAVGWDISKFPNIQRWVKD
CAVLSGYEENQEGAEAFGAAVKKNLKQ
>SlittoGSTd1
MALALYYTAGSAPCRLVLLVAAALNIQLDLNTVDLRAGEQFNPDFLKLNPQHTVPTLVDG
DFSLWESRAISRYLISKYGDENNSLYPSDPQARAVVDQRLDFDLGTLYPRFGGYFYPQIFG
GAKPDEALLKKLEEALVFLNTFLEGNTYAAGDKLTLADLSLVATVSTIDATGIVSLDKYPN
VQKWFELVKTSAPGYDKANQAGIDEFKALLASFKAKTEL
>SlittoGSTd2
MKSLACLIAVCLVINLPSNAAARSKSKMPNQPIKVYYLPPSPPCRSIIMAAKVIGVDLDLVL
TNIMEGHHMTPEYLKMNPQHTIPTMDDSGFILWESRAILAYLANAYSRDDTLYPKNPRQR
AIVDQRLNFDLGTLYVRYSALYLPMLFRGEEYDEQKADQLDEALGWLNTFLDGRAFVAG
DNLTIADISIIVTITNLEAFGYDMSGHPNLTKWFERTKKALEPYGYEDVDVAGAKMLANFL
KKD
>SlittoGSTd3
MFSFLSTMPIDLYYVPGSAPCRAVLLTAKALNLNLNLKLVDLHHGEHLKPEYIKLNPQHTV
PTLVDDGFSIWESRAIITYLVNKYGKGSSLYPEEPKARALVDQRLYFDIGTLYQRFADYFYP
QVFGGAPADKDKLAKIEDALQLLNTFLEGQKFCAGPTLTLADLSLVASVSSFEASDVDFKK
YPNIKRWYETVKSSAPGYLEANEKGLDAFKGLVNSMMKK
>SlittoGSTe1
MPLKIYKLDASPPARAVMMLAELLKLKHEAVDVNLMTGDHLTPEYLKKNPQHTVPLLED
GDFYVADSHAINTYLASKYGGAQSAQLYPTDLQVRATIDSRLYFDISAIAGNSGAIVSALL
RGDITSPTKEQTDKLNSAYEILDKFLQKTKYVAADHLTVADISLAASLSSASLLVPIDEKYS
KLTAWFNTIQQEDWYKKANGPGLEAYKAFVQSKLK

>SlittoGSTe2

MAPILYKMDASPPANAVRILSDIIGLELEVKDVNFGVLEHKSPEHLKRNPMGTVPTLIDGD

FTISESHATMKYLLSVYGGDKSESLYPSDVRTRAIVDQCMFFNVGIFFIRLKVVVLPAIFGD

LDGPTEKHKADIDEAYGIVEAYLAKNKYIAADHLTIADLSVGATTISMQPLHKLDAAKFPL

TAAWMDKLKEHPSVQKYLVPGAKILGEIVNAAWERNKKK

>SlittoGSTe3

MAPILYKLAASPPACAVRMVAHIIGLKLDYKEPDITKMEHKSPEYLKLNPLGTIPVLIDDDF

ILSDSHAIMIYLLSKYGGEHGERLYPSDIRTRAVVNQVMFFDTGILFVRIKVIALPTIMEGM

KAPTEKHLNDLEEAYGMVEAYASRYKYIAADHLTIADLSLAMTMGAAQVLHKLNHKKFP

NTLNWLSLVEKEPFFIQIGLPGIAKFNTAMQYYTARNNQ

>SlittoGSTe4

MSVSRLLLHRAPGSPPARAVMMLSDMLGLQMEYNDVKFLQLEHKSPEFKKLNPMGTVP

VLQDGDFIVSESHAIMKYLLSKYGGDKRELLYPSDVRMRAVVDQCMFFNAGVFFAAFMS

VGRETFTGRIYKPTAQHIQEIETSYSVVDAYLQDRPYVATDRLTLADLAVGATASTAQVFIK

MDADKFPRCADWISRLHEEEVFKTVMLPGVAFFAKVINKIWEHNKSKLEKK

>SlittoGSTe5

MPPILYKADASPPARAVMMVIDILGINVEQRNLNPVLREQDAPEFVKKNPMRTIPWLDEG

DFWLADSHAIMIYLIQKYGKPEHSKLYPTDSRKRATVHQRLFFDCGILFQRLRAVMAPTY

MGRLTEMSKGMIRNIVDAYAMLEDYLSENLYMADEVITIADLSIASTMATLVGLVAIDEKR

FPKLTQWYNNMNKQDYCIRINIPGGKEHSDGLKALMANTKFHQKSKI

>SlittoGSTe6

MFSQYFDHIATVTMVIRIYKKDASPPARAVLMAAEIINLKCEIQDVDLSSGPHLSPEYLEKN

TLHSVPFLEDGDFYLADSHAIITYLVSKYGAEHRSKWYPCDLSVRATVDQRMYFDTSDLF

PHIKNIVSVIVTQGVGPDAEQIKAINAGYEVLDKYLQKTKFVAANHMTLADVSCVASVSS

LNVLCPVDSKYAYLLKWWDTLKEEKWYKKANEPGLAKLEAYVKSKWNK

>SlittoGSTe7

MAMFETNSVRNTSPSTPDVMTIIIHKTDVSPPARATLILADLLGLKIETREVNLPTRQQFNP

EYLEKNPLHTVPLLEDDGLVLQDSHAIMIYLVSKYGIEHESLYPKDLAARAIVNQRLFFDA

SILFPRLKTVIYSTVKYGKPMTEAQETDIKECYDVAEKYLTDQYIAGDSLTLADISCVTTISS

LDCIVPIHSKYGRLHDWWGRLKKEPWYQKINQPGLALFGRFIKGFL

>SlittoGSTe8

MGVKLYAADFSPPVRSSMMALDIFNVPFEKITLDLLKLEHHDPKYVEKNPIHTIPVLEDGD

LILHDSHAIMAYLADTYGKDDSWYPKDIKKRALVNQKLFFTTGVIFPRLRIITYYLLQKGR

KAIEQEWLDNIEEAIGFVEQFLSRTKFIALDHVTIADVAVLSHLSSVEPILPIDPEKYPKTVA

WLEIMKATPYCKKYNEEGVKSLTAIIMHAIGKK

>SlittoGSTe9

MGVKLYALDMSPPVRACMMALGVFNVPFEKILVNVQGGEHLTPEYLKKNPLHTIPVLED

GDITIHDSHAILTYLADTYGKDDSWYPKDVKKRTLVNQKLFFNAGVMFQKLRNITYNIVL

KGKKTIEPELLDGVTEGYEFMEAFLSRSKYIAGDHVTIADIAILSTVSTQDLILPIDAQKYP

KLKAWLEELKATPYCKKYNEEGANALGAYVKKSVSS

>SlittoGSTe10

MGVKLYTLDMSPPVRACMMACEIFNVPFEKIPVDLMAGDHLTPEYLQKNPLHTVPVMED

GDVTLHDSHAIMAYLADTYGKDESWYPKDVKKRAFVNQKLFFDTAIIFPRLRNITYFIVM

KGKTTIEPELLEAVAEAYDFMEAFLSRTKYIAADHVTIADIGILAVMTSLEHILPVEPNKYP

KTSAWLENLKTAPYCKKWNEEGANALGAFVKSKVTL

>SlittoGSTe11

MGLSASKKIATGLKHCTLWKADRSPGCRAVMMALDAMNLSITEVDINIDKGEHRTPEML

ALNPLQTLPVLKDRELVLCDSHAICAYLAGRYCDSGRLLPKDPGGRSITDQHMHYNSGVL

YPRFRAAAYPILYENCNFLMPQQIQDIECAYCDLECMLVGKSWFGGSWATLSDIIFASTIST

LNILVPVDKYKFPRLSGWLYRVSEELFYVTANRKGLCEFSRRIGELSQVQQS

>SlittoGSTe12

MVLTLYKIDASPPVRSVFMAIEALDLKDVQKVDVNLFEGEHLKEEYLKVNPQHTVPMLA

DDEFYIWDSHAIATYLVTKYGKNDDLYPADPQKRAIIDQRLHFDSGILFPSLRGTVEPVLF

WGEKSFRQENLDKIKKAYEFLENFLTSSSPWLAGDELTLADICCVSTVSSMDMVLPIDADT

YPNLASWKDLCSQQQFYVAGNMPGLEQFQQLVKFKLR

>SlittoGSTe13

MGMKVYKRDTSGPCRSVFMVFELLGIKDVEYIQMNLIQREHFSEEYLKMNPQHTIPTLKD

GDFVIWDSHAIVTYLVNQYAKDDSLYPKDPKKRAIVDQRLHFDNGVLFAALKTTMVPILY

NGETAFRQENLDKIKEGYAFIEKFFSGPWLAGESITLADICCVSNISSLNEIFPIDKALYPKLS

AWFERCSKQDFYIKKNLPGLQEFQELLKVKIVS

>SlittoGSTe14

MGVKLYVMDFSPPSRACMLTCEIFGIPFEKIPVDLFKGEHLAPNYLQKNPLHTIPVLEDGD

LILYDSHAIMAYLADTYGKDESWYPKDVKKRALVNQKLFFNTAVIYRRLRNITYYVFQRG

RKTLEQDWLDDIAEGYGFLEAFLSKTKYIAGDHITIADIAILSHLTNLEYILPIDAKKYPKTS

AWLEDFKATPYAKKHNEEGAKALNDLLKKFLTS

>SlittoGSTe15

MALTLYKMDASPPVRAVYMVIEALKLPNVEYVETDLLADDHLKDDFLKINPQHTIPHLTD

GNFQLSDSHAIITYLMNKYGKNSDLYPSDPQQRAMIDQKLHFDSGILYPALRENDEPIFFGT

ATSLKPEGVAKIRSAYDFTEKFLTGSQWLAGNKLTVADISCVATVSTLNELLHIDEAMYPNI

CAWINRCAEHEFYKNGNQPGLMEFRRLLKIYLAR

>SlittoGSTe17

MAPILYKMDASPPANAVRILSDIIGLELEVKDVNFGVLEHKSPEHLKRNPMGTVPTLIDGD

FTISESHATMKYLLSVYGGDKSESLYPSDVRTRAIVDQCMFFNVGIFFIRLKVVVLPAIFGD

LDGPTEKHKADIDEAYGIVEAYLAKNKYIAADHLTIADLSVGATTISMQPLHKLDAAKFPL

TAAWMDKLKEHPSVQKYLVPGAKILGEIVNAAWERNKRIDCIIKCKIK

>SlittoGSTo1

MSEKHLQTGDALPPYGGKLRLFAMRFCPYAERTVLVMNAKNLQYDTVFVNLDHKPEWL

LNYSPKGTVPALEYEEGKGIFDSNIINVYLDEKYPDVPLQSTDPLRRAEDKIIVELFAGVQS

AFYTAAYNSQALQPNMVETYHKGLVALQDTLRARGTKFLHGETPGLVDFTLFPFLERFEA

LPLVGKPEFELVKSKYELLVNYIEAMKIVPAVKLYYLSPETHAKFIESRNKGDANYNMLDT

SGVCCMRPRKKKE

>SlittoGSTo2

MLSSTISFAVHLYSSLQGVQTIGRALGSLLPYSTMVGTNKRNFDTPHLKTGDPLPPYTGKL

RLYNMRYCPFAQRTVLALNAKNIDYEIVNIDLMDKPEWLTTKSAFGKVPALEIAENVSIYE

SLVTVEYLDDVYPQRPLLPKDPVKKAVDKILVEALSAIHAVYFKLVRGPDTITEDNITAYYK

NLTFIQQELQNRGTKFLDGDQPGYADYMIWPWFERLHVINHDLARLDKQKYKLLVEYID

NMFQDPVVAEYKLPREALEHFQNQYKLGKKPVYDYLIQK

>SlittoGSTo3

MSFYQQRGVGPLPPPALTDKLRLYHVDMNPYGHRVLLILEAKKAKYEVYKLDPLRLPEW

FRNKNPRLKIPVLEIPTEYGDKYLFESVVICDYLDEKYTRNQLHSRDPYVKAQDRLLIERF

NELIKGSLECFDTNFAFGSEQIIQTLDIFEKELTLRGTNYFGGHRPGMLDYMIWPWVERLY

LLRCVNERKFDEKRGLFPNFADWSDQMQLDDVVKKHANSPQEYFDYYKNARTHSMGY

YL

>SlittoGSTs1

MPKYVFHYFPIKALGESVRLLLAYGGEGFEDHRIDLDDWPKFKPNTPFGQMPVIEFDGKQ

YAQSIAIARYLGNKYGLTGDTLEDNLEIDQNVYLINDLRIKAASAHYEKDEVIKEQKYKEL

SKGAFPDSLEMLNALFAKNNGHVALGKLTWADFMFAGLFDYLSAMMRMPDLGQKYPAL

QQVKDRVYSLPKVKAYADAVPA

>SlittoGSTs2

MPKYVFHYFDGKGLGEPVRLLLAFGDEGFEDHRVAFKDWPDFKPKTPFGQMPLLEFDGK

QYAQSLSIARYLGKKYGLAGESLEDALEIDQNVDLINDLRAKAAIASYEKDEAVKEKKYA

EFNKDVFPNMLEKLNEIITKNNGHIAIGKLSWGDFVFAGMFDYIKHLLVVPDLEKKYPAFQ

KVVDAVYSIPKVKTYADAVGPTEF

>SlittoGSTs3

MPKYVVHYFNGKGLGEPIRLLLAYGGEDFEDHRVSPKDWPEYKKNTVFGQMPNLDIDGK

KYAQSIAICRYLGNKYGLAGDTPEEALEIDQFVDLLVDFRLKAAIVTYEKDEKLKEQRYEE

YSKDVFPEQLDRFNDIIEKNNGHYALGKLTWADFVFAGFFDSIKFIVRIPDLEKKYPAFQRV

IDHVYSIPKVKAYADALGPTEF

>SlittoGSTs4

MPEVVFYYFPVKALGESVRLLLAYGGQEFDDRRIPQTEWPAFKPKTPFGQMPVLVIDGKE

YAQSLAISRYLGNKYGVAGDSFEDALEIDQNVDLINDLRAKAAVVHYEADEALKEAKYA

DFVKNVFPDLFEKLNAIFVKNNGHVALGKLTWGDFVFAGMFDYLKVMLRMPDLEKKYP

VFQQVIDNVYSIPKVKAYADAAPPTDF

>SlittoGSTs5

MAKKLHYFNFIAIAEPIRYILHYTKQEFEDVRHDHRFWPNAEFKEKLPFGQFPLYEEGDRM

LTQSLAIAKYVARGTDLIPSDPWTQAVLDAAVYTIYDYWSKVVTFIREKDPEKKKELKREL

LDETIDFFFSRLDKDLKENGGYFSGKLSWVEFVLCGLVEASNYFLDTELEKKYPRVEALIK

KIKSLPGVKEYVAARGPNVFKQ

>SlittoGSTs6

MPKKLSYFNLNGLAEPIRYILHYTNQEFEDVRYDVYSWPDPKIKASLPYGQFPVYEEDGR

VLTQSLAITKYVARGTDLVPTDPWDNAFIESVIFSIMDYWKNVVPTVREPDPAKYTELRNE

IIEKNIPYYFSRFNKHLEDNGGFLNGKLSWGDFVFCGVIEASDMFLGIELSKDYPAVGSLVQ

HIRNLPGVKEYVKSIGPYTIDLARFKYKK

>SlittoGSTt1

MSLKLYCDLMSQPSRALYILLKSIKCNFEPKFVNLRQAEHYTEEYSAINRFQKVPVIDHNG

FILTESVAILKYLSRENVIPESLYPKESKAQARVEEFLEWQHAGLRLHCAMYFRVKYLNPV

MFGKPADPVSLAGYESRMEAALTEFNDKWLGRGTDFINGNTPTVADLLAACELEQPRMS

GYDPSANFKNIQVWWKKVREHFNPYYDEGHVIVNKIIKKNTQVASKI

>SlittoGSTu1

MVLKLYYLEDGPPSLSCRQTLEALGVPFEIVNVSFYHGEHMTEEFAKKNPQKELPVLEDD

GFFLSESVAMMQYICDKFKPNSPLYPTDSKARAIINHRLMFAMTTYYAELLNYFIMPVYFK

YERTPEVLKRVHRALDLFETYLERENTSYAAANHLTIADFPLINATMAMEVTDFDFSKYKR

ITKWYNDFKKNQPKLWSITAEGMRLMRKCVENPPDLSHLNHPIHPARKAE

>SlittoGSTz1

MGDVVETKPVLYSYWRSSCSWRVRIALNLKEIPYDIKAVSLIKGGGEQHCNEYREVNPME

QVPSLCIDGHTLVESLNIMHYLEETRPNQRPLMPQDCFKRAKVREICEVISSGIQPLQNLIV

LIYVGEEKKKEWAQHWITRGFRAVEKLLSACAGKYCVGDETTLADCCLVPQVFNARRFH

VDLRPFPIILRIDRELENHPAFRAAHPSSQPDCPPEVAK

>SlittoGSTz2

MAENRAILYAYWLSSCSWRVRAALHFKGIPFEERSIDIVKTNQQKTEQFRAINPAQKVPAL

VIDNVTLVESMAIVQYLEDTHPEPALTPKTPVLRARMRELCEVVVSGIQPLQNIGLRSHFD

TTEQYTKFTKYWTDRGLKTLEDLLQKSAGKYCVGDQITLADLCLVPQLYNAVTRHALDIS

KYPTVSKLYESLLKENVFKETHPESVKSKM

>PxylGSTs1

MAKKLHYFDVNGIGESIRYILHYGGQKFEDVRYQISGWPDKKVKDALPFGQLPLYEEGDR

SLHQSLAIARYVASQSKLLPNDLWEQAVVDSYVMTIYDFWFTKVIPFVKETDAAKKATLK

KEILDESIHFFFSRFDKVLKEHNGFFIGKLTWAEFILVGIIEAGNLFLDYEIEKNYPHVKAAV

QKVLTLPGVKEYIAKRKVYAL

>PxylGSTe1

MMRKLLFRNSPTDLVRCKSITLYGDEISPPVRFVQMTADLLGIQYKFKKVDLFKQENKQD

FFKKINPLQKVPALKVGDTVITDSHAIAMFLCENSDGQTLYPDDPIIRPIVQQMMFFNSSTL

FNIDSTIYSNFFAGSESIDANLVKDWTLALDYLEYQLRKHEWLAYDKMSLCDLCCGATVS

TLQLLIPPTEKHKKVNRWIKRLEEIPCHSINKIGLDRLQFLIDQIKDVAH

>XM_011563164.1

MPVVKFYYFPIKALGEGPRLLLAYGGQEFQDIRVDKESWPEFKPKTKYGQMPILEIDGKQ

YAQSAAICRYLASRYGLTGADAEQNFEIDEAVDFFNDIRAKAAQVHYEEDEKVKEKRHET

YSQTVYPDLLGKLHDIVQRNNGHLAANKLTWADFYFAGVYDYMKVMLRRPDLDQQYP

GFAKVYETVYSLPKVKAFADAAPKTDF

>XM_011552863.1

MPLILYVSDASPPARAVLMLAEILQLEFDKQYINPVLREQDSPEMTQKNPMRTVPTLEDGE

FCLADSHAIILYLMEKYGQNHRNLYPEDLRVRSTIHQRLFFDCSILFPRLRAIMAPTFLGKL

SQPSGSMVTNIEDAYRTLEAYLSQSKYLAGPEMTIADISAVATVSGLDGLHPIDEKRYPNTR

EWLVNMSRKTFYRKCNAQGNEMLISLLKSSMENNKQNDKKAKL

>XM_011567066.1

MKLYKLDGSPPARSAMMAVEALGLAVDAVDCDLLKGENTSPEYMKKNPVHTIPLLEDGD

LTIHDSHVIVTYLVDKYAKDDSLYPKDIKKRAQVDQKLYFDATILFPRLRAVTYPMIAEGLT

KPTEKMLSQIEEAYSMLDAFLSGSKWLAGDSMTVADICAVGSVTGLNHIVRLDTAKYPNV

AAWLNTMKQQPFVKSKNEPGDTFLGQWIASKLTSS

>XM_011554817.1

MAIDLYLTPGSAPCRLVLLTAAALNIQLNLNHVDLRAGEQFSPDFLKLNPQHTVPTIVDQG

FALWESRAISRYLVNKXGHGSTLYPEDPQSRALVDQRLDFDLGTLYPKFADYFYPQVFGSA

PADPEKLKKLHEVLGFLDIFLGDEKYAAGSDLTIADLSLVATVSTIDAAGISLDDFPNIHRWF

ELVKSTAPDYENANGKGIKAFKELVAQLNAKTEL

>XM_011553851.1

MSLKLYYDLMSQPSRAVYILLKKSNINFEPKYVDLRKGVHYTDEYSNNINRFKKVPVIDH

NGFILTESVAIIRYLGRENLLPEALFPRADKVMNTRLDEFLEWQHLGLRAPLAMYFRVVMF

SPDSEKIPSYQKRMETALDEFSTLWLGRGNQYILGDTATVADLLAACEVEQPRMTGYDCT

ANYPAIREWMDRRRSYFNPHYAEASAIVEKIAAKRVPMKKPTAKL

>XM_011567078.1

MKLYKLDMSPPARATMMVAEALGVKVETVDVNLMKGDNRTPEYLKKNPIHTVPLLEDG

DLILHDSHAIVTYLVDKYGKSDALYPKDVKKRAQVDQKLYLDATILFPRLRAVTFLIITEGL

KKPSDKMLKDIEEAYSILNSFLSTSKYLAGDQLSLADISAVATVTSLVYVLPLDEAKYPKVA

AWLKTMKDLPFVKSKNEPGVTQVGQWITSSLTSK

>XM_011554818.1

MPDLYYVPGSAPCRAVLLTAKALNLNLNLKLVDLHHGEQMKPEYLKLNPQHTVPTLVDD

GLAIAESRAILTYLVNKYGKGSTLYPEDPKARAIVDQRLYFDIGTLYQRFGDYFYPQLFGG

APEDKEKLAKVDEALKFLDTFLEGQKYVAGNNLTVADLSLVASVSSFEAANIDFLKYGNV

KRWYETVKATAPGYEEANGKGLEAFKGLVNSIMKK

>XM_011553444.1

MWVYGYGSLIWKVDFPYETKLVGYIKGYLRRFYQHSIDHRGVPEKPGRVVTLIPSDKLDS

QVWGVAYKIREEDIKKVTEHLDFREKNGYSKKTVTFYPRDIQNQPFEITLYLATEENESYA

GPKPIEEIAAQVVSCAGPSGSNKEYVYNLADAMRQLVPEVKDDHLFELEAAVKKVDPDL

KRC

>XM_011555305.1

MCRLECLMAIVNTLPFRLQQRVTKLYFQVLTRNQHGAHSEKQFNEYNKMDVKILPALQD

NYMYLIVDKATNEAAVVDPVDPKSVLEAVEQHGVNLTTVLTTHHHWDHAGGNEELVKL

KPGLKVFGGDQRIPALTEKVAHDTRLNIGRLNVQCLFTPCHTTGHICYFVAPPEEGEVKAV

FTGDTLFLGGCGRFFEGNAQQMHNALITVLSNLPDATKVFCGHEYTLQNLKYAAHVEPD

NEDVKSKIAWAEEKRAQQLPTVPSSIGEEKSYNPFMRVNSPSVQQFAGKNNPVETMKAIR

DAKDHFKG

>XM_011563034.1

MAKKLHYFDVNGIGESIRYILHYGGQKFEDVRYQISGWPDKKVKDALPFGQLPLYEEGDR

SLHQSLAIARYVASQSKLLPNDLWEQAVVDSYVMTIYDFWFTKVIPFVKETDAAKKATLK

KEILDESIHFFFSRFDKVLKEHNGFFIGKLTWAEFILVGIIEAGNLFLDYEIEKNYPHVKAAV

QKVLTLPGVKEYIAKRKVYAL

>XM_011570866.1

MDQLQLVSSNKICGGFQKVYSHESSELKCKMNFSIYFPPQAEGGDSKLPVVFYLSGLTCTE

QNFISKSGFQRYAAEHGIIVVGPDTSPRGVKIEGDDESWDFGVSAGFYLDAAKEPWSKHY

RMGSYLNKELYDLVLQAFSSVVDPNRIGIMGHSMGGHGALVSTLRNPGQYKSVSAFAPIC

NPCACPWGEKAFTGYLGDDKSKWAEWDATELVKRYDGPPLTLLIDQGGADKFYTDKQLL

PENLVAVCRSAGVPVILNLREGYDHSYYYIATYIGEHFDFHAKILKA

>XM_011551708.1

MKSSMFTVVFVVSVLCNAAARSKSSKMPVEPIKLYYFPPSPPCRAVMMAAKAMGIELEM

VLTNIIEGEHMTPEFLKMNPQHTIPTIDDNGFILWESRAIIQYLANAYGRDDSLYPKNPRLR

AMGDQRLNFDLGTLVSRYLNLYSPVLHGEPFSDDMDAKLKEALGWFNTMLEGRAFSAG

DNLTVADISIVVVFSNLEAFGYDFTAYDNVSKWFERTKKALEPHGYKEIDQAGAQMLASFI

KKD

>XM_011569182.1

MVLKLYAVSDGPPSLSVRQTPAAVGAAGLAALQLPHELVSVNYGAGEHLTEGYAQMNPQ

KEIPVLDDDGFFLSESNAIMQYLCDKYKRDSPLYPTEAKARAIINQRMCFNLASYYANISA

YTLAPIFFDYERSSLGLKKVHLVLEGFDTGGCRRTRGGWARATPPPTTSPSPTSRSSTPP

>XM_011568007.1

MTIAFRTINKLLVPVAGNVFCLFRAQLSTVKMADNNPDYKAAKSIHEFTVKNIKGEEVKL

DAYKGHVCIIVNVASQCGLTANNYKQLNELYEKYAESNGLRILAFPCNQFAGQEPGNPEEI

VCFASERKVKFDLFEKIDVNGDSAHPLWKFLKNKQGGTLGNFIKWNFTKFIVDKEGVPVE

RHGPNTDPLDLVKSLEKYW

>XM_011560668.1

MPDIELYEMQGSAPCRAVRLTARALGKPLTAHHLDLMAGEHLKPEFVKINPQHTIPTIVDD

GFALWESRTIMRYLVNKYSKGSSLYPEEPKARALVDQRLDFDLGTLYAKYAVYFYPQVFGT

APENAEDLKKLNEALAHLNTFLGESKYAAGSNLTIADFSLVATISTIDVSDIVDLKQYPNIV

KWYEHLQSSVEGYGEENLAGLENFRSFIKEFKAKKAAAK

>PxylGSTs1

MAKKLHYFDVNGIGESIRYILHYGGQKFEDVRYQISGWPDKKVKDALPFGQLPLYEEGDR

SLHQSLAIARYVASQSKLLPNDLWEQAVVDSYVMTIYDFWFTKVIPFVKETDAAKKATLK

KEILDESIHFFFSRFDKVLKEHNGFFIGKLTWAEFILVGIIEAGNLFLDYEIEKNYPHVKAAV

QKVLTLPGVKEYIAKRKVYAL

>BmorGSTs1

MPKVVYHYFACKALGESGRMLLAYGGQDFEDHRVLSADWPDFKPKTPFGQTPVLVIDGK

QYAQSTAICRYLGRKYGLAGANDEEAFEIDQNVEFLHDIRAKAAAVYYEADEELKAKKH

EDFSKNVYPDMLKKLNSIVEANKGHIAAGKLTWGDFVFTSMFDYLKTMLQIPDLEVQYP

AFKKVLQSVLTQPKVKAFLDLGRPYEFEF

>CmedGSTs1

MPSYKLTYFPVKALAEPIRFILSYGGVEFEDDRFDRNDWPKIKPTMPFGQVPVLEIDGKKV

NQSTAICRYLAKQHGLAGKDDWENLEIDVAVDNIHDLRSKIGAYHYESHPEAKEAKREPL

MKELLPFALDRLDEQVKKNGGYFVGGKLTWADMVFVALLDYLNFMTKFDITEKHENLK

ALKEKVLALPKIKAWVEKRPQTEC

>SlitGSTs1

MPKYVFHYFPIKALGESVRLLLAYGGEGFEDHRIDLDDWPKFKPNTPFGQMPVIEFDGKQ

YAQSIAIARYLGNKYGLAGDTLEDNLEIDQNVYLINDLRIKAASAHYEKDDVIKEQKYKE

FSKGVFPDSLEMLNALFAKNNGHVALGKLTWADFMFAGLFDYLSTMMRMPDLGQKYPA

LQQVKDRVYSFPKVKAYVDAVPA

>EsemGSTs1

MPKTTVYYFPLKALGEGIRLLLAYGGEEFEDRRVAKEEWPEMKPKMPFGQMPVLEIDGK

KYAQSSAVVRYLGRKYGLGGKDIEEDFEIDQSVEFYTDIRTKAGAVFHEQNEEAKARMQA

ELEKNYYPFALKKLDEIIKKNNGHLALGKLTWADFLFAGMYDCFKKILQVSDLDEKYPSF

KKLQDTVYAIPKVKAFADKAPKAEY

>SlittoGSTs1

MPKYVFHYFPIKALGESVRLLLAYGGEGFEDHRIDLDDWPKFKPNTPFGQMPVIEFDGKQ

YAQSIAIARYLGNKYGLTGDTLEDNLEIDQNVYLINDLRIKAASAHYEKDEVIKEQKYKEL

SKGAFPDSLEMLNALFAKNNGHVALGKLTWADFMFAGLFDYLSAMMRMPDLGQKYPAL

QQVKDRVYSLPKVKAYADAVPA

File S2

Sequences used to phylogenetic tree analysis to PxylCYPs

>SlittoCYP6AB14

MITLAIALVALVALYLYGTRNFSYWQKKGVKHDKPIAFFGNNVASYLMKKSITQMAVDAY
WKYPKEKAVGFYRSTSPELVIRDPEIVKRILITDFQYFYPRGLNVHKTAIEPLMRNLFFADG
DLWRMLRQRITPAFTSGKLKAMFPLIVERAEKLQARALSAATANREMDARDLMARYTTD
FIGACGFGLDADSLNEEDSAFRKLGVSIFDFKPKDVFVAMLKEMFPEACKNLKYLERLEK
PMFELVTAILKQRNYEPVGRNDFIDLLLECKKKGKMVVESVEKVNPDGTAETVSMELTDE
LIAAQVFVFFAAGFETSSSATSYTLHQLAYHPEVQKKVQDEIDRVLAKHNNKLSYDAIKE
MTYLECAFKEGMRMFPSLGFLMRQSAHKYTIPELDLTIDPGVNVVIPLQALHNDPQYFEE
PHVFRPERFLPDQVDEKTKFVYLPFGDGPRACIGLRLGLMQSLAGLAALLSRFTVAPGPST
LRYPVVEPKSGIVQSIKHGLPLSFTVRNPESVATTL

>SlittoCYP18A1

MITVIYNSKLLWGLWWVSNTVVSRISLPLLLVAAMALLVLRRVSLLTEMRKLPPGPWGPP
VVGYLPFLGVRHKTFLELARNYGALFSAALGNQLTIVLSDYKLIREAFRREEFTGRPNTPL
MHTLDGLGIINSEGRLWKSQRRFLHEKLREFGMTYMGNGKKIMEARIKSEVYELIANLHR
AEGAPIDANPLLALGVSNVICGITMSVRFSNGDARFERLNNLIEEGMRLFGEVHYGEYVPL
YNYLPGKAQAQERVAKNREEMFAFYQTLIDEHRETLDVDNVRDLIDVYLIEIEKAKTEGR
AGELFEGRDHELQIKQILGDLFSAGMETIKSSLLWMIVFMLRNPDVKRRVQEELDAVIGRE
RLPAIEDMANLPYTETTILETLRMSNIVPLATTHSPTKDVHLNGYRIPAGSQVIPLINCVHM
DPNLWDEPNKFNPSRFMDETGKIRRPEFFMPFGVGRRMCLGDVLARMEMFMFFACMMH
QFDVQMETGDAPPSLEGTVGATIAPQAFRVKFSPRAIPAAAAPDHPHLRHVGAH

>SlittoCYP18B3

MYFYYFLVFLAINFLSSAILRIFMYSTLIFWIIFKNVKYYKSLPPGPWGLPLLGILPFLNRRPP
HLVYLNMAKKYGDVFSVQMGSNLTVCLGSTKLMKEFFSRNDSTGRPHTPLNNLLGGLGV
ITSEGSLWKRQRVFLHEKFRALGVKLWPNTRFERDINVEIDELMMELNRSEGKSVNPAHV
LGRHIHNVICQLMMSFRFTADDAEFKLFNEKVSRGMRLFGLVLIGEHVKAYLKLPGKMAV
INEIKKNLADVSNFHKEHIVARIQQREQSSEPRQPADLLDFYLDHLEAEKQLMTKNEKHNI
FPDVEPVQQIVQIMNDLFSAGMETSLTSLLWTCIMMMKHPEVAEKVRADLREVVAPGERV
TMAHRLQLPYIEAVLIETMRMVSIVPLGTTHVNTAEWKIGNYTIPAGTHIVPLIHRMNMDP

DIYMEPEQFRPERFIRDGKFVITDTFMQFGIGQRMCIGNLLARMELFLFFSNIMNNFEFRMP

EGEDIPGFDGILGATHAPLPFKLVFKKLEA

>SlittoCYP304F4

MIVLTILAALFLPYLIIYLYKNAYNKPKNFPPGPPSLPVYGAFWIVLAHGFTDLATAFKKLG

EKYKTKIIGLYMGPVPTIVLNDPELIKEMLNREEFDGRMDIIIFRLRSFWKKLGIFFTDGYF

WHQQRRFSLRYLRDYGFGRRCEDLESVVATEVKEMIDLRIGGPKYPAEMDIVKGDLVYM

PYFFAIPFLNGMLQVFSRSTLPRSEYKALWALTRGTVLFQRSSTDMGGALSLTPWLKDILP

NYSGYNDLVKGNQYLLNFFKKLINEAMETHDETYDRHFLDMYITKMKEEMREKDKTTY

SVDQLILTCTDYTFPAASAVQFVLTILVERLLLQPEIQDKIHEEIDRVVGRDRLPNLDDRRN

MPYLEACIRETMRYDTTVPLSVPHRAMKDTTLAGYDVPEGTMVSANLTMLHMDKDIWG

DPENFRPERFLKNGELDLAATVIAIWSRQKIMCW

>SlittoCYP305B1

MIPTFLLLFVVTLILYGFISTIKPRKYPPGPVWFPFIGSSGILQKMTKKYGSEWRACLELSRQ

YSTNVLGMRLSTELLVVVFGEKNVRQVFNEKEFDDRPDNFFARLRCLGYKNKGITFANGE

VWKEHRQFAVKNLKYVGYGKTLMEKEIQNELSSLLEQIKENNDKPINIVNLLGESVINVL

WKFVAGERIKEEKLKHLLDLFRQRGKAFSVAGGMLNQMPWCRFIIPELSGYSLIKKLNGEI

SKVIEEAIEKHKKKEVEGHDFIYKFLNEIDANKNATFTEEQLKIVCLDFFIAGSQTSSNYLG

FAFLKVLKSQDIQEKIFNEIDTIIGDRPPCWNDNERLVYTSAFIQEVHRYYPVVCMAGPRSL

QADTSIDGYRIPKGATVLMSLADIYFDPEIWDDPQVFRPERFIDESGMLKNSEHLYPFGSGR

RRCPGDSLAKSFIFIMFVGILQKYRIHVSNGTVPSEIPVIGILSSPRPYTAEFTLRK

>SlittoCYP306A1

MDFFFLWLVTFVAGFWIFKKIKEWQNLPPGPWGLPIVGYLPFLDRHQPHLTLTKLSKQYGP

IYGIGMGSVYAVVLSDHKLIRDAFAKDNFSGRAPLYLTHGIMHGNGIICAEGSLWKDQRKL

ITMWLKSFGMSKHSVSREKLEKRIASGVYELLENVEKSEGSPMDLSHMLTNSLGNVVNEI

IFGSKFPPDDKTWNWFRQIQEEGCHEMGVAGVVNFLPFIRFISASTRKTIEVLTRGQAQTH

RLYASIIDRRRKVLGLAPVKEAAYPLHENLFNEHPDGHIKCIKYSKHASEHEEHYFDPNILI

PTDGECILDNFLLEQKRRYESGDEGAKYMTDEQMLYLLADMFGAGLDTTSVTLSWFLLY

MALYPEEQEIVRKEILSVYPEDGEVDGSRLPHLMAAICETQRIRSIVPVGIPHGCLQDTFIG

NYRIPKGAMVIPLQWALHMDPDVWEDPEVFRPQRFLAEDGSLLKPQEFIPFQTGKRMCPG

DELSRMLACGLVARLFRRRRVRLATDPPSTKDMQGTVGVTLSPPTVSYYCDPL

>SlittoCYP6AB31

MIVIAVVVVLLITLYFYGTRNFKYWEDRGVKHDKPLPLFGNNLQGYLFRSSMTQVADEIY

WRYPNEKVVGFYRSSRPELLIRDPEIAKRILITDFAHFFERGFTPHKTVFEPLMQNLFFAEG

DLWKLLRQRMTPAFTTGKLKAMFPLIVERAEKLKTRALTAAAEGKSLDARDLMARYTTD

FIGACGFGLDADSLNDEDSPFRKLGIKIFKMRPQEIITAIAKELFPELGRNLKIWTRVENDIN

ELVVEILKKRDYKPCGRGDFLDLMLECKQKGVMVGESIQNKKPDGTAETVTLEFTDSLIP

AQVFVFFAAGFETSSSATSYTLHQLAYHQDVQKKAQEEIDRVLAKHDGKLSYDAVREMH

YLENVFKEGLRMFPSLGFLLRQCTKSFTFPDLNLTIDEGVRALIPLQSLHNDPKYFPEPHVF

RPERFEPDEFDANNKYVYLPFGGGPRACIGERLGLMQSLAGLAAVLSCFTVEPAKETVRH

PVVDPLSSVVQSIKAVCHSCSGRERNMLELT

>SlittoCYP6AE47

MFLYLFVTLLILFYYVITRKYDYWKVRNVAHSKPTFLLGNYGDMILQKKSFGENIQDCCN

KFPEAKYIGVFFGTEPVLIPKDPEIIKLIVTKDFYYFNGRELSKYNHLEPAALNVFATYGDN

WRVLRQNMTPLFSSAKMKNMFHLIEKCSYVFEEMLDREIKISNELEVRSMTSRYTMECIG

ACAFGVDTNTMAEGVEENPFKKIAAFVEPPTYVVYVRIIRSIWPSIFYLFGGRLLKKQLEFF

KNLITSVMSKREYKPSGRHDFVDLVMSWKQSHQIVGDSIKNMKTGEHKKVTLEANDDLL

VAQCFVFFAAGFETSSTTLSYTLYELAKHEEIQTKVLDEVDAYLARHNNKLNYDCVTELP

YLEAVIDEALRFYPVLGMIPRELMEDYTMPDGTKLTKGLRVHLPVFYLHHNPENYPEPEV

FRPERFLGEEKRNINPYVYLPFGEGPRTCIGMRFAKMQMLAGLITMLKKYRPEFLADGMP

TKLKFKAQSFITHPVGGIKIKFIEREGWESRVFAKSS

>SlittoCYP6AE48

MITLYLLLAIPLALYGVYLVSKRKFRYWEQKKVPHLPPKPILGNFSEYILQQKYYGHVEQD

ICDKFPEEPYVGSYFGTEPALIVQDPEFIKTVMTKDYYFFSGREASAYSKNEALTQNLFFTY

GDRWKVLRQNLTPLFSSAKMKNMFHLIEKCARIFENMIDQDVQKCKDIEVRTLTAKFTMD

AIGNCAFGVETWTMVKTENNPFTPFTKIGDIIFDTARLRALKVVMRSIWPAIFYGCGGKSL

PADVDNFFYNLMTGIFKGRNYKPTPRNDFVDLLLKFYNNKTVTTGDSMKNMKGDSEEK

VTLKVDEEFLIGQCFLFFAAGYETSATTLSYTLYELAKNPKAQELAIQDVDNYLRRNDNEL

KYECATELPYVEACVDEALRLYPVLGVLTREVIEDYTFSTGLKLEKGLRVHLPVYHMQHN

PKYFPEPEQYRPERFLGEEKNNITPYTYFPFGEGPRLCIGMRFAKMQITAGIITILKKYRVEL

APGMSEKLQFEPRSIITAPIGGIRLKFIEREGWQQRVFKAPSEA

>SlittoCYP6AE49

MIQKLCQKFKSEPYFGAYYGTEPVLIVQDPEIIKHVLTKDFYYANGRESSNYSNGELITQNL
FFSAGDNWKVVRQNLTPLFSSLKMKNMFHLIEKCTIGFEDMLDDEIKMSNVIEVRRIAAR
YTMDCICSCAFGVESKTMSQKENNPFQLMAEEIFESSRYRGFKLIFRAVWPSIFYRIGLKIFP
STINRFFFKLLTGVFESRGYKPSPRNDFVDLLLNLKKEDYITGDSISNVKTGDDKKVHLKV
DDELLVSQCILFFSAGFETSATTMGFTLYELAKHPEVQKKAAEEVDAFMCRHKNKLVYDC
VTELPYLEACMYETLRMYPVLGNLTREVMDDYVLPTGLKLDVGVRLHIPVYHMHYNSQ
YFPEPYKYKPERFLPEHKNEVNPNTFFPFGSGPRLCIGMRFAKMQVLSGLITFLKKYKVEL
ADGMATELDFDPKTILTQPLDNCIGLKLTEREGWEQRRFVRT

>SlittoCYP6AE50

MIFLYLILVFIFLFYYVISRKYKYWKIRNVPYKEPTFLLGNFTDMFLDKKFNGEIMDDICKQ
FPDDKYVGAFNGIEPVLIPKDPEIIKLIMTKDFYYFHGRALSKYNDKEPGGLNLFGTFGDN
WRVIRQNMTPLFTSAKMRNMFHLIEKCSHEYEAMIDREIKISKEHDVMPMTTRFTMDCIG
ACAFGIDTKTMTEGEDDNPFRRIIRSSKPRKSMIYQGMLRSLYPEIYYALGFKMPSTQLDFL
PKLVTSVMNQRDNKPSERNDFVDLIMSWKQKHQIVGDSIRNMITGEHEKVVLKASDDLLI
AQCYVFFAAGFKTSTTALSYTLYEIAKHEDVQNKVFNEIDSYLARHNNKINYDCVSELPYL
DAVIDETFRVYPVLSVIPRELMEDYTMPDGTKLDKGVTVQLPVYHLHHNPKFYPEPEVFR
PERFMGEEKKKINPYVYLPFGEGPRVCIGMRFAKMQMLAGLITMLKKYRLELADGMPTK
LKFKAQSFITHPVGGIKIKFIEREGWESRVFAKSS

>SlittoCYP6B42

MLLLYLPIVLTFFIYILYLYFTRTFKYWKSRKVRGPEPTVFFGNIKESALRQKNIGVIMQEIY
NNYPNEKVVGVYRMTSPCLLIRDLEIVKHIMIKDFEVFGDRGVEFSKKGLGQNLFHADGD
TWTALRNRFSPIFTTGKLKNMFYLMNEGANNFVEHISTECQKKQEFEIHSLLQTYTVSTIS
ACAFGVGYDSLDDKLETLKLVDQIISAPTYANELDMMYPGLLTSLNLSIIPVPIQKFFKNLV
DNIIAQRNGKPTGRNDFMDLILELREMREVNYTKYGNFVKPLEITPEVMAAQAFVFYVAG
YETSATTMAYLMYQLALNQDIQNRLIAEIDEVIQRNTGEVTYDMIKEMKYLNKVFDETLR
MYSIVEPLQRKATRDYQVPGIDLVIEKDTIVLISPRGIHYDEKYYDNPDVFNPDRFDPEEVG
KRHPCAYLPFGLGQRNCVGMRFGRLQSQLCIVKVLSKFRVEPSKNTDRNLKVTPHRAIIGP
KGGIHLNIIPRKIKA

>SlittoCYP6B48

MSALYFLAAVPVLAYAIYYYFTRTFNYWKSRNVAGPEPTAFFGNIKESVVRTKNIGIVMHD
IYNAFPNEKVVGMFRMTSPCLLIRDLDIVKHVMIKDFEAFSDRGVEFSKEGLGQNLFHAD
GETWTALRNRFTPIFTTGKLKNMFYLINEGGDSFIEYINTECQRKQEFDIQPLLQTYTLSTIS
ACAFGITYDSLDDKFETLKLVDKIFSAPSYANELDMMYPGLLKALNLSLFPSSIQKFFENLV
KTVIEQRNGKPSGRNDFMDLILALREIGEVTNAKYDSAKPVEITPEVMAAQAFVFYAGGY
ETSATTMTFMLYQLAMNPDIQNKLTAEIDEVLQANNGQVTYDSIKEMKYLNKVFDETLR
MYSIVEPLQRKAVRDYQVPGTNLTLEKNTIVLISPRGIHYDEKYYDNPEQFNPDRFDPEEV
GKRHPCAYLPFGFGQRNCIGMRFARLQSQLCIVKLLSKFQVEPSKNTARKLEIEPCRFIIGP
KGGVRLNIVPRKLKA
>SlittoCYP9A51
MIVLLIWVAVLIAVVVLYLRQLYSTFSRHGIKHFKPVPLLGNMSKVLFRRQHLANNIVELY
NAFPEEKFVGRFEFLNEAVLLRDLELVKKVCVKDFEYFVDHRSFGIDSFFARTLFLLRGQE
WKDMRSTLSPAFTSSKIRLMVPFMVEVGDQMIDSLKKRIENSKGGYIDIDCKDLTTRYAN
DVIASCAFGLKVDSQTEQNNQFYEMGKTVTNFNSFQLIKFFLMMNIPKIAKNIKLSHDDV
QSNDDATGFATVEESSVGLRKVNREWSDNDLVAQAFLFFVAGFETISSSVCFLLHELAVNP
DVQERLAQEISEYDAKNGGKFDFNSIQSMKYMDNVVSELLRRWPVAAVTDRICEKDYNM
GKPNATAEKDFIVRKGAGIMVSAFTFHHDPQYFPEPEKFDPDRFAEENKHKINPNAYMPFG
VGPRNCIGSRFALCEIKVITYQILRHMELSPCEKTCIPAKLATDNMNLRLHGGHWLRFRLR
K
>SlittoCYP9A52
MIILLIWVAVLVAAVVLYFRQLYSSFDRHGIKHIKPVPIFGNMGGILLRIDHICDNIVKLYNDF
PEERFVGRYEFVSELILVRDIELIKKIAVKDFEHFLDHRSLFSNSESYFSRNLFSLRGQEWKD
MRSTLSPAFTSSKMRLMVPFMVEVGEQMMQSLHQSIKQSKDGSIDIECKDLTTRYANDVI
ASCAFGLKVDSHNDKNNTFYALGKETSSFNFRQMMTFFLLINAPALAKLLKLDFLSESSKE
AFQKLVLGTMENPEFKKILRPDLIHLLMEAKKGQTAHDDTTPKVYRLDLQL
>SlittoCYP321A11
MVLLPITLVLITLVLAWYFVGTYNENYWKKRGVVFYSKNKVLGPMWDFLVSPRPLFQVF
GDLYKQYRHEPAIGMGQLLTPAMFVINQKNVQQVLSGDFQSFNHRGIESIEGDQLSDNITF
MNGPRWKLMRSNMTPLFTANKLKNMYYIMDKSAQDFVQYLKNNPKTIEGNLFETLMMF
CNAAVCGAIFGIGSESIFDTPFLKVAKGLSQSNLSVRLKFVGFLLSPKVCSFLGLKMFKEHE

DFFVKSIGEVLKRRNQENVKRHDFADIALAIQKNGTMKDRTTGYEIEPTTGILSAQALFFLT

AGVEPCANAIYATLYFLSSHPEILEKVHQEIDEHFEKHSNNITYDVICDLEYTEKVLSEAMR

IFPPIGFLTRQCVQNTVLPIGNIPVEKGTKIFTPIYDIHHDPELYPDPEVFDPERFSKDRRPND

DIYMPFGMGNRTCIGARYAKLQVQAGLVHVLRNFTVQLSKTSGKLKFAHDPLNVRLRNIG

FELVPRGIK

>SlittoCYP321A12

MVFLPLTLVLVTLVLAWYFIGKYNENYWKKRGVAFYTKNKVVGPMWDFMTSQKALFEIF

GKLYKEYRKEPAIGIGQFLTPSLFVIDPKNVQQVLSGDFQSFNHRGIESVEGDTLSDNITFM

NGPRWKLMRSNMTPLFTANKLKNMYYIMDKSAQDFVQYLKNNSKLQRSDLFETLMMFC

NAAVCGAIFGIGSESVFDTPFLKLAKNLSQSNILVRMKFIFYTLSPKIYTALGFKLFKEHEEF

FVKSIGEVLKRRDQENVKRHDFADIALAIQKNGTMKDRTTGYEIEPTTGILSAQALFFFTA

GVEPCANAIYATLYLLSLYPEILEKVHQEIDEHFEKHSNNITYDVICDLEYTEKVLSEAMRIF

PPIGFLTRQCVQNTVLPIGNIPVEKGTKIFTPIYDIHHDPELYPDPEVFDPERFSKDRRPNDDI

YMPFGMGNRTCIGARYAKLQVQAGLVHVLRNFTVQPIAKNQWKKLKFAHDPLNVRLRNI

GFELVPRGIK

>SlittoCYP324A6

MRNIMYYIFVLPLCIISLWLYSKWLSMKNYWKKLGVPHEPIHPLFGSMSFLQKKNPGTWM

MELYQKYRSSPYVGIWIFWRRGLVVNDPDLSRKILVKDADIFRNRMLASGKSDPMGALNL

FTVDDPLWTSLRRRLTSVFTGSKLRGWQELYQSKVEDLIYRINSDNEKGIINELRPLFADYA

TDIIGESSFGIQCQSTRNSTGPLRLMTKEFEKYSQWRGMAWSSIFFLPEMVDILRFSFWPKN

TVSYFKKVFKAMVEERGGFDKDIDGKRDLLDALLKLKIDAKKENQDMDLDILISNAMIFL

QGGYDTSSTALTYTVYELAYHPKYQKILYDELVEAEKALNGKPFDAESLAKLPYLEAVIKE

SLRKYPIMGWLDRKALRDYQVDENLTIPANTVVYVNAIGLHYDPKYFPNPEEFRPDRFLP

ENANNIVPYSYIPFGEGPRMCIGKRIGMNTVRYALAAVVLNFEILPLDQFPLPNDIPIEKKGL

FYHPAIPLSVELKEGNKNYNP

>SlittoCYP324A1

MFAILLFMVVLFFSLWLLNKWRKVKSYWLEKGVPHYPPHPILGSLTFLQRENPSIWMRRV

YNDFKAPYVGMWLFWRPALVVNSPEIARRILVKDADVFRNRFVSSGKSDPTGSLNVFTVN

DPLWSKLRRRLTLVFTAAKLRSLNGITIAKTKDFIRRLEIESKKPDLIDLRKICTDFTTDVIGE

SAFGITSNSLTDGDSIMRRVTREFQAFNLHRGLSWSSIFFFPELVDIFRFSFFPKDTMKLLRK

IFRTMVEQRGGYDKDIKDARDLLDALLKIKKEADMENEEMTEDILLAQAAVFLLGGFDTS
GAALTWTMYELAWNPLYQEKLYEDILNMKRKIGDRGFDSTVLAEVGYLDCVIKETLRIYP
PMGWLDRIATKDYQIDEKLTIPAGTAVYVNGVSMQMDPEYFPNPEVFNPDRFLPENERSIK
PYTFMPFGEGPRNCIGMRFAYQTLRQALAEILLKYEIKVIPGTLKPSEIEIEKNGMFYMPSG
KMSVQFVQRDNEFVNEL

>SlittoCYP338A2

MFFESFLLNLSVIVVLIIALIIDYVTKFFSYWYVRHVPYKSSIPFFGGDYHRVLGIRSSTEDA
NILYAKHPKDKYVGRVKCRIPDLIVKDPDTVKRMLSTDFANFHSRGLSLDKSQDVCLRDN
LFYADGEKWTLLREGYDSLLSNMNCELESLSDCLPGTNGDANVQEILSKVLDKVFEDLLL
GSGGGSVVKEVRSAIQRRTLIERFKSYLKDIFPSLYIMFGLTTIFGEPSSKTKSVIEKSKVLSQ
IKNLGNVYQLGIKEKNRKRHSDVEFAYSIISSFITEGYIPCLNALTALIYELALNPEVQEKSR
NSVAKHNRDEYLDVAIKEALRLHPPYSIISRQCTKTYHPEGGILIDRKITINVPVMALHRDE
ENYINANSFNPDRFLDDEGASKHCYAYLPFGAGPRKCIGEQLALKLIKSISKAVLNKYEIEP
CAKTPSKLTMVDHNFGRVVDRDIWLRFKVRAXRK

>SlittoCYP354A9

MDIIMDQLKSGLKTLLLMMMEDWKMYLCLTILISLYLYYTSTFNFFEKRGIPYRKPVIFLG
NLGPRLRGKQSFHEFQLETYNYFKGNRFGGIFEGRRPLLTILDPDLIKAITIRDFDHFTDRM
AMNAKEPKFWSRSLLNLKGSEWKAVRSTLTPVFSSSRLRHMLPLIEMCSKQMVEFLNQY
DKKDVEMKQTIGHFTLEVIGACAFGVKCDALSDENAHFFKVAEKFDYMPMHKRIILYFILI
FVPQLIKYLNFSFLNLDSSKELVEIFEIAKEERQ

>SlittoCYP4G75

MSYTTAESVVPTSTWAATNLFYVLVVPALILWYAYWRMSRRHFYELAAKLHGPPGLPLLG
NALEFTGGSHDIFRNVIEKSIPFDGESVVKIWIGPRLLVFLYDPRDVELILSSHTHIDKADEY
RFFKPWLGDGLLISTGQKWRSHRKLIAPTFHLNVLKSFIDLFNANSRAVVNKLKKESGEFD
CHDYMSECTVEILLETAMGVSKTTQDQSGFEYAMAVMKMCDILHLRHTKIWLRPDLLFK
FTDYAKTQTKLLDVIHGLTKKVIRRKKEEFNSGKRPTILNDYSSTAEETTKTTSVEGLSFGQ
AAGLKDDLDVDDSDVGQKKRLAFLDLLLESSQSGVVISDEEIKEQVDTIMFEGHDTTAAG
SSFFLSMMGIHQDIQDKVIEELDKIFGDSDRPATFQDTLEMKYLERCLMETLRMFPPVPIIA
RHLKQDITLPSCGKQVPAGTTVVVATYKLHRRPDVYENPTKFDPDNFLPEKSANRHYYAF
VPFSAGPRSCVGRKYAMLKLKIILSTILRNFRVYSDLKESDFKLQADIILKRAEGFKVRLQP

RKMAKAC

>SlittoCYP4L12

MILLLVSVVLVFALLVSWISLVRQSRRFNVDGPSPLPLVGNAHLFVVKSSEFLNLVQRLSEK

YGKVFRVHFFSTPYVVICHAKQAEELVSSIEHITKGRSYSFLTCWLGQGLLTSTGQRWKTH

RKFLTPAFHFNILQNFLPVFCKNQRVLTEKLRGLADGRPIDMFPIIALAALDNVTESIMGVC

METQKDSQSEYVKSIEELSAIVTMRMQIPFFGEDAIFNLLPYKTRQDKALKVLHGQTNKVI

EARRQELKKAKITALPDNNDIGLKNSMRSWTCCCSLKLMGKKIFFEKCLGNWYQRGRQN

PFLGGRRPYFIRLPFRGRQDKPWKGFPGQDKQRDGSKRTGTEEGKITASPDNMDIGIKNK

HAFLDLLWLAEMMGKKIDDESVREEVDTFMFEGHDTTTSGIVYTLHCLSKRRDVQEKVY

EELKTIYGNEMHRDPTYHELAQMKYLELVLKESMRLFPPVPLIERRIMKDCEVGGLKLLK

GTSVVMNIYQIQRQPDLYDDPLEFRPERFEAPLKNPFSWLAFSAGPRNCIGQKFAMMELKI

TISEIIKHFYILPAAQEPELSADLVLRSKNGVQVKFMPRK

>SlittoCYP4S8v1

MIFELLLAALVAAVVWLIFFRDDDNPVDKLPGPPRKPWIGNAWELLQMPSDKLLDILVDY

TNTYGDRYVFKVFSLRVLHISGPPDVETVLSHSKNIKKSAPYDFLKGWLGTGLLLSTGLH

WHKRRKILTPTFHFNILKNFAHVIEEKTQDLVKMLKDKNGADVSLMPTISDFTLFTICETA

MGTKLDDDKTSATVDYKNAILNIGMQMLARITRSWLHNDFIFNNSSRGKEFTKTLEVARA

FADNVIMDRKAQRIQNKGEDISIADASDGIGTKKRLAMLDLLLEAEEKGEIDMDGIRDEV

NTFMFEGHDTTALALTFGLMLLADHEDVQERIYEECQTILGDSEHVTMSNLSDMKYLEAV

IKEILRLYPSVPFIGREITEDFKLGDITVKKGTTVDVHIYELHRRADMFPEPEKFMPERFLGT

EMKHPYAYVPFSAGPRNCIGQRFAMQEMKTTLSELVRHFKIVPKVKGARPRIMADLVLRP

VDPIYVKFIPR

>SlittoCYP340AA1

MIFAVLTVLFCALLALWNKLKPRGPSPPVYPGALPILGHAHQLYGDPIYFWKIQKKMYQFS

LDNGGVSEIRFGPHSVYVVTDPDDSLVVANTCLNKPYYYEFAKEMFNRGLVTASLTTWKP

HRRLLYPAFNMQVLATFVSEYNTQARNLVEALTSEVGKEPFNVRHYLVNQLLKTVCQTSL

GLETKDKSIDEDYANATEDLFMLFVERLKKLYLHLPWIFAWSSLKARQDQLLKIMNDAMF

RVIDRRKAELKGNVDKTKTDIRDVKFKPLLDQLLELSDTQDALSDAEIREHLDTVVAAAY

DTTSIAVTYALILLGTYPEVQNRAYKEVQTVLGDEDKDFSKDDLQRLVYLEAVLKESMRM

YPSVPCVARSIEADVKLKNYTLPAGASCMISIYGLNHHAIWGPDVDQFKPERWLDPSTLPD

NPNVFCSFSIGKRNCLGRLYAMLVMKTMLAHVLRKFLIISDINSITTQYDILIKPADDSQIRL
ELRSG

>SlittoCYP340AB1

MSVIASILIFCIVFILWFWLRPRSPNAPPCYPGALPIIGHGHQIFGNRKHLWKYLEKIFQYSL
EKGGVLEVWLATHSVYVITDPDEILTVANTCLNKAYFYEFGKEYFKNGLITSEASIWRNHR
KLLDVAFTQKVLDTFVDEMNAQTRVLVTNLNVLVDKGPFDVRQYLINYTLKIVSRTSLGL
EAKDQTMIDEAYADALEDLIKVYCERAQKVWLHLSCIYDRSSLRKKQDYLLKIMNNIIDA
VIVKRRSDLKLNNNESTQCDKAKPGKFKPALDQILQLVDKHHVFNDEEIPETLDTFVAAAL
EEHHVFNDGEIREHLDTFVAAAYDTTSETLIYVLLAIGSHPEVQEKILKEIQEVQPNKNEDI
SKYDLLRLVYVEAVIKEVLRLYSPLPGVARKIEKDVKLKNYTLRAGSTCILSLYGVAHHSM
WGPDVNEFKPERWFDVANFPTNPNAFGAFGIGRRNCIGKQFALTLMKLTVVYLVRNYHIS
SDISKLESEYDIVLKAVSGHLISITYRE

>SlittoCYP340K4

MQGRFHYYETDDCVRTMLSLLLFVCVLCIASVWSKYARGRPKYPLPPAMPGALPFVGVL
HQVLKNYSRRWDFLKSKAEECAKLGGVTHAHFGSELYYVITDPQDALTASNQCLKKHYA
FDLAKIWMGDGLVLSSGDIWKRHRKLLSPAFTLPIIHSFLDVFNSQAKKLASSMEPHVGKG
LFDPFLKLKLNALETFCVGTLGIEALDDVNFTEKYMESVDDIVTLWISRVVKVWLLSDVV
WKLTGLKKKEQELVDTLHTMTNKVLQRKKAALKNKGVEKVIESQTSGIKYRPFLDLLLE
LSTNGAFTDDEIREETDTIIAAGYDTTSNQITFALLLLGAHPDEQEKVYEELLAVLGPDRDV
EKDDINKLVYTNAVIMESLRLFPSIPSLFRTVETDVKLKNYTMPAGSYCVIFPIAASSVDPS
WGPEPDKFRPERWLHGDFRNNKEYSAFGLGKRACIGRTYAMISMKVTLAHFLREYRVKA
DLSNLKLNFDFLMKPISGHDISIESRRI

>SlittoCYP341A13

MFVWLLLLVFVLWMFTAWARRRHMVKLAAALPGPRDELPGIGLAHKFIGDTEAIMHQLQ
LFSYESMENDGVLRSWLGNTLFFIVVNPGDLEMVLKTCLEKDDLHRFIRTIIGNGGIFAPVS
IWRRRRKILVPAFSPKVVENFVHVFAEQSDKLVKQLSRCSGKGKFQAWPFLSTYTLDSVCE
TAIGVKINAQENPDSPFLRAMCDILNMICKRIFHLWLQPDWLFKILPMYKKHQECIHTMH
NFTDRVIESKREELKREKESKPEVDHEFDLGAYQKQTFLDLLIHLSGGERGYSNVELRKLL
TLPSPVPLTIAGTDTLAVAIGFTLQLLGKYPEIQDKVYLELCEVFEDDRMLVKEDLMKLKY
LERVVKESLRLFPPVPLIIRKVLEDITLPSGRVLPAGSGVACSIWGVHRDPRYWGPDAERFD

PDRFLPERFNLEHACSYMPFSNGPRNCIGYQYALMSIKTALSTILRKYRVIMDTEESPYPYI
RVKIDIMMKAVDGYELRLERRERDHHI

>SlittoCYP341B3

MLWLVLLVVTLGFTFRWFKNRRILQVASQIKCDIKAYPLLGHAYLFIGGGESRMRTFERLG
REAIKNGGLTNMWLGDNYFTVVVDPVDLEVILKSSLEKDDVMRFARNLIGNGTIFAPVSI
WRPRRKVLAPTFSPKNLNNFVKIFSRQSSVLAKQLQTEAGRGPFSLWKYLTSYTMDSVCE
TALGVNVNAQKKEDEPFLKAFEKCSILIAERMVQPWLYADAVYKLLPYHDAFEKSKTVIC
DFIDKIIKSKRIAIKENKDDANKNSNDSKTFLELLIEGSGGERGYSDQELQEETLVLAVAGT
DTSAVGASFTVSMLARYPAVQEQVYKELVEVFGDSDRLVTAEDLLQLKYLDAVVRETLRL
YPPVPVVVRKIEKDLELPSGITLVKGCGVLVHIWGTQRNPRYWGDQRKQFRPERFPDTPLP
PSRCFLGFNSGI

>SlittoCYP366D1

MVLSVYIAGTLFLCICVLLWSLERRERNKYRGVPTLPLLPFIGNLHQAIGDTQHIFHVVEHI
TEICEEKQSLFVFWLGPCPILAAQHPDDVKMITNSFVEKPYFYDFGKAWLGDGLITAPGYI
WKQSIKKLSGTFTSSVVDGYQDVFNAQAQKLVINLKTKVGQEPFDVMHELAFATLESICQ
TALGVSNISESIVTEEYYNAFNRCLELMIYRGVNILLHPDAIYRLTPAHKEMMKCVAVLHK
VSDNVMKKLRAERNDVKTSEMRGNQNPGTNKGPKFKAFLDILLELRELDPNYTDDQIKS
EVDTIIVAGQETVATAFFYTLLMIGCKPGVQEKMYAELHNIFGDSKRPVCKEDLARMRYCE
AIINETLRLYPPAPGVMRYADRNLKLKSFTIPKGTICAINFWGAGRSLQAWGPDAKVYRPE
RWLEDQPPGNPAAFLPFSYGRRACIGKKYAMAILKTMLAHCVRELDFKSQAANLKLKINL
ALPPVSGHPHTN

>SlittoCYP367A6

MLWLTITLIITAGFLGWQYWRWRHRRMLELAARLPGPPALPILGNALSFMFNPGEFLNKV
DVFMEKYGEVFRFWLGPELNIVVKNPTDIRALLSSNKVNQKGPLYEFLVPFIGYGILSGGPI
WRAHRKIVTPSYNKKCVDNFSPIFNREAEQLARVICKKDPKRSFDIYKDVVRCTTQSVNQ
TLMGLSKEDSQNLYRMDEMLWATQKMYGLIFEKMTKWWLHIPIIYWLLGKKKQQDYYV
KLIDELMADIVRRRRKALEVSVPGEEYMGIVDRYILSGELTEKEIQSETMTLFTTSQEAAA
KMASGVIMFLAHLPDWQDKVYKEMMEVVGADGPVTNEQLKQLEHLDMVYKETLRYFS
IAALIQRTVVEEITIQDGSITLPVGTSLVIPIHNLHRDPRFWEDPHRVMPERFLPENVKKRDP
NAFVPFSLGPMDCLGRVYATALIKTIVVWVLRYARLEPAGTLDNIKLNVAISVSCADGYNI

KARPRNGRINELS

>SlittoCYP367B6

MYWPLYLIGGALVALWLLWRYKNRRMIQMGNKMPGPPTIPLLGNALMFMCQPEAFIKVL

KDLMAEYGNVTRFWLGPDLNIVVSNPDDIKLLLSNTKTSIKGPQYKYMADVLGGGILSGS

GPTWRRHRKIANPNYGKRAIEGYSEVFNQEVDLLLKKFRSMPREQFNVYDHVVRTTSYA

VCRTLMGLTKDQTMKLPHIEELIDTSPCLYDIVFRKMNKWYLPIDPVSGFVKMMTDFCEV

ILQHRTESLKTLEDSKRDLMNSEDDSSVNTELSVIDRFLLSQELDKDELVQETFTIFTSSQEA

TAKISSYILLMMAYHPKCQEKLFAEIKNIIGDEDRYVTDDDLKRMPYLEMVFKEVLRLYPI

GAMLQRTVNEDIAISNYTLPAGCSFVAPIFHLHRDPRFWTEPDRFDPERFNPENVKTRNPN

AYIPFSLGQMDCLGRFFGTKLVKTLCVRVLREFELSSTETYDDLRVIISISVASLNGFPIILNP

RKKLT

>SlittoCYP333A6

MIMRNRALSECYTLFYIRSHSSFQNVRRSYSSAPPAQVKPYASIPGLSSLPILGPIHHFLPVIG

QFGPGVNIFDLMSTLHEKYGPIVRLQGVFSRADFVILYEPELIDQVYRSQEANPLRPGFQTL

EYYREVIKKGSLDGVYGLTTAQGEKWRDFRTKVNPALLKLKLVKVYAPPLGEIAQDLVQR

IRRYKDDTQYLAKNLDPEITKWSLESIAYVALGSRLGCLGDEVTKDHPGFTLLQCSKDIIE

YVFQLEFLPSVWKYFATPAYKKIISTYDKQWEISTKYIEEARKRINARGHDIPEEEKSIVERL

LAIDEKVAILMANEMLFAGIDTVAFTAISVIYNLATNPEKQQRLREEIRSPDPHKRYLRACL

KEALRMRHVIPANLRRTDRDHFVAGYHIPAGVDVIAPNEYLSGLEKYYPRPKEFLPERWL

ADKSDPIYYGNAPAIVTLPFGFGIRSCIGRRIAELEIETLMKKLFDEFEVTWEGPPIKVVTKV

SNMFLEPYNFRFKSVK

>SlittoCYP333B3

MQTTKLRPLFQLISINKEVIRTVAVASTAVKTDALKPWNEIPGPPSLPIIGQIHHFLPGGTFGD

MKDPDTIMRLLDTYGPAVRLDSMLGKPPLLFLSDPDSAELVLRSENWLPYRPGFQSLEFYR

NDYKKDKGRVTGLISDHGEVWKEFRSTVNPVMLQPKTIKLYAKVLDEVAQDMIVRMKA

NRDEKNMITKDFDKEMNLWALESIGTVALGCRLNSFDPNLPADSPEWQLIQCVHDLFSTA

NELDFKPSIWRYYATPTFKQAMKLYEHHENKQAMKLYEHHENLTKYFINKGKEQLKTKP

DNEKGVLEKLLEINEEVAHIMASDMLFAGVDTAANTVTATLYLLAKNPDKQAKLREEVTS

NVEKRSYLRACIKESLRLMPVVSGNARRTTKEYNIMGYQVPKGVDVIFTHREMSVMEKY

YPKAKEYIPERWLTNKDDPLHYGNAHPFAYGPFGFGARSCIGRRIAELEMDTFVARLIENF

QVEWFGPPPTVVQEALNYIKGPYNFVFKDIK

>BmorCYP4M5

MFVYLIFIASFFLLIHLAFNYNSKAVMMNKVPGPKLSFILGNAPEIMMLSSVELMKLARKF

ASRWDGIYRIWAFPLSIINIYNPDDVEVIVSTTKHNEKSSVYKFLKPWLGDGLLISKGEKW

QQRRKILTPAFHFSILRQFSVIIEENSQRLVESLEKCIGKPIDIVPVVSEYTLNSICETSMGTQL

SDKTEDAWKAYKDAIYELGPYFFQRFTRVYLYFDIIFYLTSLWRKMKKPLKSLHGFTSTVI

KERKIYVEQNGVKFGEDVNDDDLYIYKKRRKTAMLDLLIAAQKDGEIDDHGIQEEVDTF

MFEGHDTTASGLTFCFMLLANHRAVQDKIVEEINDIMGDSTRRANLEDLSKMKYLECCIK

ESLRLYPPVHFISRNLNEPVVLSNYEIPAGSFCHIHIFDLHRRADIYEDPLVYDPDRFSPENS

KGRHPYAYIPFSAGPRNCIGQKFAMIEMKSAVAEVLRKYELVPVTRPSEIELIADIILRNSGP

VEITFNKRTK

>BmorCYP4M9

MWMYFILILLLLLTIHFLLNYNYRARLLRRIPGPRGYFIVGNALDVILSPAELFASTRKNAA

QWPNLNRFWSFGIGALNIYGPDEIEAIISSTQHITKSPVYNFLSDRLRDGLLLSTGTKWQKR

RKILTPAFHFNILKQFCVILEENSQRFTENLKGTEGKSINVVPAISEYTLHSICETAMRTQLGS

ETSEAGRSYKNAICELGNQFVHRLARLPLHNNFIYNLYTLGKQNKHLNIVHSFTKKVIKDR

RQYIRGNGGNNFDDEKDTQADEHSIYFNKKKTAMLDLLLKAERDGLIDEIGVQEEIDTFM

FEGHDTTATGLTYCIMLIANHKSIQDKIIEELDEIFGESTRAADIEDLSKMRYLERCIKESLRL

YPPVPSMGRILSEEINLNGYTVPAGTYCHIQIFDLHRREDLFKDPLVFDPDRFLPHNTEGRH

PYAYIPFSAGPRNCIGQKFAILEMKSLLSAVLRRYNLYPITKPEDLKFVLDLVLRTTEPVHVR

FVKRNKV

>BmorCYP6AB5

MFFYLLIVILVALYYYGIRTFDYWKKKGVNHDPPLPFFGNNIRQFMQKASMAMMATETY

KKYPEEKVVGFYRGTSPELVVRDPELIKRILVTDFSSFYARGFNPHKKVIEPLLKNLFFADG

DLWRLIRQRFTPAFSTAKLKAMFHIITERAEKLQMIAENEAYENFCDVRELMARYTTDFIG

ACGFGLNIDSLSDENSQFRKLGKRIFKRDLSDAVRAALKLMFPELCKHLTFLTPELEKSMT

YLVQNVIREKNYKPSGRNDFIDLMLELKQKGKLLGESIEAKNANGTPKQVELEFDDLLMT

AQVFVFFGAGFETSSTASSYTLHQLAFNPKCQEKTQKEIDKVLSKHNNKITYDAIKEMTYL

EMAFNEAMRLYPSVGYLVRMCTVPEYTFPEINLTINEDVKLMIPIQAIHKDEKYFKDPERF

HPERFSSGAKANLKPYTFLPFGEGPRACVGERLGQMLSMAGLVAVLQKYTVEPVEISLRD

PIPDPTTTVSEGFVHGLPLKLRRRERRI

>BmorCYP6AE9

MFLLINICVILFVIYYLVTKKYSYWRNRNVSHEKPVLLLGNYGDLILQKKNFGEMARAIC

QKFPGEPVVGAFFGTEPVLIPQDPEVIKTILTKDFYYFNGREISEHVHKELLSYNLFATYRD

EWKILRQNLTPIFSTAKLKSMFTLIEKCSKSFQNLLEDETKISKELEVRTLMQRFTIECIGSCI

FGVDTDTLGNDKMNPFKAAGSQLSDFSRLVFVKGIVRAIWPTLFYALGFKTFTTELDIFKK

LVNAVFAQRKHKPTTRNDFVDLILTWKNNNTITGDSIGSFKNSDKTKFSIDVNDDLLLAQC

LVFFAAGFETSAMTSSYTLHELAKNQRALKKACDEVDAYLLRHGNKINYDCVTELPYLE

ACIEETLRLYPVLGIITREVMEDYVLLDKIHLKKGDRIHVPVFHLHHNPEHFPNPEEYRPER

FYGEEKRKVKPYTYLPFGEGPRICIGMRFAKMQSIAGLITILKKFRLELPEGAPTKIEFKPEA

FVTTPKDLIKIKFLEREGWQQRVFV

>BmorCYP6AE22

MYVLTFLLHFILFLVLVVYYITKRNHDYWKNKKVPFEKPYPILGNYGDYILLKIFFGNVTK

RLCEKFPESPYVGTFYGTEPALVVQDPDLIRLILVKDFFYFHGREVCIYVDREITTQSIFFTY

GDDWRVLRHSLTPLFTTSKMKNMFHLIKNCCRVFENVLEEKATVKSFEINSIIKYFIMDCV

GACLFGVEINAMEKHSQNPIVKISKEIFPKSTTRGIINVLRGIWPSLFYALRLKLFPEVITMF

FTDLLECAFKDRQRNLSQRQDFIDLFMKLRQNKYLVGDSIPDIKNGRVQKVNLEVTDELLI

AQCVSFFGAAFETSSTTLSLTFYELAKNPKYQKRAIEEVDDYFAKHNNEIEFDIVSETPYLN

ACIDETLRLYPSLANLTREVMEDYTFPTGLKVEKGTRIHIPVYHLQRNPKYFPEPNKFDPR

RFLPEAKQTIYPFTYMPFGEGHRICIAMRFAKMQMLAGFATLLKKYEVAVDDTTPQELTID

PRIIVTTPIENIQLKLIARQHAL

>BmorCYP6AU1

MLTAYLLYVFASIVVLIYFYLNNKYKYWKNKNIAGPEPEFLFGNLKESFHRRKHIATVFKEI

YDQYPDEKVVGMFRMTSPTLIVRDLDIVKQIMIKDYAKFNERGIKFSKEGLGDNLFHADA

DAWKAVRSHLTPMFSSGKLKNMVRVLSQTGDRFVDHVTNGVRLRPEQELVSLFLKYNIAT

IMACSFSMETDINDMQVYEMLNVAVFENSYISELDMVFPGILRKTNRSIFGPTVNNFCYEI

VEFIKNERNGKPANRNDMMDMLLGVNCDKLKMKNGSEDFVEITEHVMAGQVFVFFAAG

YVNNTITLTFSLYHLAKDQSIQERLMREIETVLENHNNVLTLEAINEMSYLEMIFLETLRMH

PTTNTLQRSALEDYTIPGTDIEIEKGTLVLIPPLAFHHDEKIYPEPEKFDPERFSVENHKSRH

ACAFLSFGIGPRTCIGIRLAKLQYKVCMVKFLTKYRVAPSTRTKDAYKIDPLRTALEPEGGV

YVDILPRS

>BmorCYP6AB4

MLTAAIFVIIVALVYLYSTRTFRYWEKRGIKHDKPVPFFGTDSEGYLLRKSMTQTAVDAYL

KYPNEKVIGFFRSSRPELIIRDPDIIKRILTTDFAYFYPRGLNPHKKVIEPLMRNLFFADGDL

WRLLRQRMTPAFTSGKLRAMFPLVVERAERLQSRTLEIASQPLDARELMARYTTDFIGAC

GFGLDADSLNDEDSAFRKLGAAIFNITVRQAIVAALKEIFPGIFKHFKYSSKYETDFMSLVS

SILKQRNYKPSGRNDFIDLLLECRMKGEIVVESIEKMKPDGTSEVVRMELTEQLLAAQVFI

FFAAGFETSSSATSFTLHQLAFHPEIQEKVQKELDQVLAKYNNKLCYDAIKEMRYLESAFK

EAMRMFPSLGFLIRECARQYTFPELNLTIDEGVGIIIPLQALHNDPEYFDSPNEFRPERFMPS

EYNHNKTKFVYLPFGDGPRGCIGARLGLMQSLAGLAAVLSKFTVKPAPNTKRHPVVEPKS

SIVQSFKDDLPLLFIERTKP

>BmorCYP4G25

MSYTNAENVVPTSTFSAINLFYVLLVPAVILWYAYWRMSRRRLYELADKLNGPPGLPLLG

NALEFVGGSADIFRNIVQKSADYDHESVVKIWIGPRLLVFLYDPRDVEVILSSHVYIDKAEE

YRFFKPWLGNGLLISTGQKWRSHRKLIAPTFHLNVLKSFIDLFNANSRAVVDKLKKEASN

FDCHDYMSECTVEILLETAMGVSKSTQDQSGFEYAMAVMKMCDILHLRHTKIWLRPDLL

FKFTDYAKNQTKLLDIIHGLTKKVIKRKKEEFASGKKPSNLNETATTSEPSTGKLTSVEGLS

FGQSSGLKDDLDVDDDVGQKKRLAFLDLLLESSQSGVAISDEEIKEQVDTIMFEGHDTTA

AGSSFFLSMMGIHQDIQDKVIEELDQIFGDSDRPVTFQDTLEMKYLERCLMETLRLYPPVPI

IARQVNQEITLPSNGKKIPAGTTLVIATYKLHRRPDVYPNPNKFDPDNFLPERSANRHYYAF

VPFSAGPRSCVGRKYAMLKLKVILSTILRNFRVISVLKESDFKLQADIILKRAEGFQVRLQP

RKRMAKA

>BmorCYP305B1

MLPVLVCIIVIVLICCNVIRSVIKPEKFPPGPIWYPFFGSSSIVQQMTSKHGSQWKALLELSK

QWSTQVLGLKLGRELVVVVYGEKNVRQVFSESEFDGRPNSFFYKLRCLGKRLGVTFVDG

PLWREHRQFTVKHLKNVGFGKTSMELEIQNELKLLREYINDNKHKPIKVDSMFSSAVMN

VLWKYVAGERIREDKLERLLELFYLRSKAFTLTGGLLSQIPWCRFIIPGLSGYKLIVDLNQQ

ISEIIEEAIKKHLNKEVQQNDFIYSFLDEMNEENKASFTYDQLKTVCLDLIIAGSQTTGNAV

KFALLSVLRNKNIQEKIFNEIENTIGDSMPCWADSSKLVYTSAFLLEVMRIHTIAPLAGPRR

VLQDTVIDGYVIPKETTVLISLADIHLDPNLWPDPHEIKPERFIDEKGLSKSNEHIYPFGSGR

RRCPGDSLARSFVFIIFVGILQKYRIDCVNGVLPSNEADIGLLAAPKPFVANFVSRE

>BmorCYP6B29

MAIIYILLASVVLPLLLYLYFTRHFNYWKKRNVPGPKPVPLFGNLMESALRKKNIGIVFKEL

YENFPNEKVVGIYRMTTPCLLIRDQDVIKNIMIKDFDVFVDRGVELSKSGLGANLFHADG

DTWRVLRNRFTPLFTSGKLKNMLHLMIERANKYIEHVEMLCDHQPEQDIHTLVQKYTMA

TIAACAFGLDIDTTDPNKDQLKTSEEIDRLSLTANFAFELDMMYPGVLKKLNSTLFPGFVS

RFFKDVVKTIIEQRNGKPTDWNDFMDLILALRQLGDIQATKRNSEDKEYSIELTDELIEAQ

AFVFYIAGYETSATTMTFMLYQLALNPDIQDKVIAEIDQGLKESKGEVTYEMLQNLTYFEK

ASNETLRMYSIVEPLQRNAKIDCKIPDTDIVIEKGTTVLFSPLGIHHDEKYYPNPSKFDPERF

SPANISARHPCAHIPFGTGPRNCIGMRFAKIQSRVCMVKMFSKFRFELAKNTPRNLDIDPTR

LLLGPKGGIPLKIVRR

>BmorCYP9A19

MIIIIWTLVIGLALLLYLKQTYSYFSKHEIKSVTPLPILGNMGKIVFKFNHLVDDISQLYNNFP

EERFVGRYEFVNPVIYIRDIEIVKRITIKDFEHFLDHRTIVNEDSDPMFGRNLFSLKGQEWK

DMRSTLSPAFTSSKMKLMMPLIVEVGEQMINAIKENIKNSGVGYVDIDTKDLTTRYANDVI

ASCAFGLKVDSLTEENNRFYAMGKAATNFSFKQILMLLGFISFPKLMKMTKFRLFSEETSG

FFKELIMGTMKDREMRKIIRPDMIHLLMEAKKGKLVHDNKSSKDTDAGFATVEESAVGKK

QIDRVWTDDDIIAQAVLFFIAGFETVSSAMTFLLHELALNPEVQEKLVEEIKENKERNNGK

FDYNSIQNMAYLDMVVSELLRLWPPAVSMDRICVQDYNLGKPNDKAKRDFILRKGTGVAI

PVWAFHRNPEFFPDPQKFDPERFSEENKHNIKPFTYLPFGVGPRNCIGSRFALCEVKVMAY

QLLQHMEISPCEKTCIPSKLSKEIFNLRLEGGHWVRLKIRD

>BmorCYP9A22

MITLIWLGVLLVTLTLHLRKVYSRFKDYGVNHFTPIPVLGNAGPITVRLRHVAEDFDMVY

KAFPEDRFTGRFDLLRPTVIIKDLDLIKQITIKDFEHFLDHRALVDDTADPFFGRNLFSLRGQ

EWKDMRSTLSPAFTSSKMRGMVPFMVEVNNQMIDMIKKKIVANADGYLDCEGKDLTTRY

ANDVIASCAFGVKVDSHTNEENQFYLMGRDMADFGFRKIMVFLGYSSFPKLMKKFNAK

LLSDETGHFFTDLVLRTMEDREVKEIVRPDMIHLLMEAKQGKLSYDEKSTKEADTGFATV

EESDVGKKTINRIWSNTDLIAQATLFFVAGFETISSAMSFALHELALNPEIQDRLVQEIKENY

AKTGGKFDFNCIQDLTYMDMFVSEVLRLWTPVVGMDRLCVKDYNLGRANKNATKDFIL

RKGEGLSIPTWSIHHNPEYYPEPYKFDPERFSEENKRNIKPFTYLPFGTGPRNCIGSRFALCE

VKVMLYQLLQQIEVLPSDKTKVRAKLAKDTFNVKIEGGHWIRLKLRD

>BmorCYP339A1

MLMCQVRHLQLHGRLHLLSRFLSRSPNSSQSAIPQQLSKDDLKQEHSPIGALPVENKNTN

EIFPTARVIPMMLTNKESVVLPFDDVPGPKSLKYLSTVRQYLSDIGTQLTAGILTFGVNIGTF

INSRKPLKKFSALFDEYGPVVRFISPVGNDIVLINHPDHIQKVYTIEGDCPVRSTLDSLEKY

RIEHRNHIYGGLYTLNGEEWSRQRSIIYTPLHNAVTYHIQGIDDICEYFSQKIYNMRNHQDE

IPKDLYKDLHKWAFDCLGLILFSKKFSMLDTDLVYSQCDMSWMYHSLEKATEAVIKCESG

LQWWKILSTPAWYSLVKYCDSLDSLIGKYVLEAEQAISYKAKEIEEQYPNSNILINAMLLG

QEKMNPEDIATVIMDMLLIGVNTITSSTSFLLYYLAKYQKAQKILYKEIQENFPEQKIMDLT

KIREQTPYLQACIKETLRLTPPIPVLTRILPKNITLDKYNIPRGTLIIMSTQDASLKESNYDDA

NTFCPERWLKSDSNEYHLFASIPFGYGARKCLGQNIAETMMSLLTVKLLQKYKLEYHYGD

LEPTRSFIARPKRALKLRFIDRI

>BmorCYP307A1

MSSLIIVFFVFALAVYKLLRRKTERWVKTNKYGGVETAILRTAPGPVCWPIIGSLHLLGGHE

SPFQAFTELSKKYGDIFSVKLGSADCVVVNNLSLIREVLNQNGNVVAGRPDFLRFHKLFA

GDRNNSLALCDWSNLQLRRRNLARRHCGPKQHTDSYARIGTVGTFESVELIQTLKGLTSR

SDASIDLKPILMKSAMNMFSNYMCSVRFDDEDLEFQKIVDHFDEIFWEINQGYAVDFLPW

LAPFYKKHMEKLSNWSQDIRSFILSRIVEQREISLDTEAPEKDFLDGLLRVLHEDPTMDRN

TIIFMLEDFLGGHSSVGNLVMLCLTAVARDPEVGRKIRQEIDAVTRGKRPVGLTDRSHLPYT

EATILECLRYASSPIVPHVATENANISGYGIEKGTVVFINNYVLNNSEQYGSEPEKFDPSRFL

EKTRVRTRRNSQCDSGLESDSERAPVGKPDVEREMLSVKKNIPHFIPFSIGKRTCIGQTMVT

SMSFTMFANIMQSFEVGVENINDLRQKPACVALPKNTYKMHLIPRK

>BmorCYP6AE21

MSVSALVFAAFVLLVTYIYYWSTRKFEYWKRKNVPYAKPVPFFGNYMRYITLQSYIGDV

MQKLCQQFPDRPYFGSFYGTEPALVLQNPEIIKQVFTKDFYYFNSRDNSDYNHKEVVTQN

LFFANGDKWKVVRQNLTPLFSSSKMKNMFYLVEKCNHSLEDMLDRETKDLQIIEIRAAMI

RYTLDSICSSAFGIETNTLSEGGEYSPFANMASIIFSPSIARGLKWISRSMWPGIFYKLGLQC

FPAEIDDFFERLLTEVFENRGCKPTNRNDFVDLILSLKQNDYLTGDGLVPKNVDAKKVTVK

VDDALLIAQCVVFFGAGFETSATTLSAALYELAKNPEAQRRAQEEVDELLLKHNNKLNY

DCLAELPYLEACMNEAMRLYPVFPNITRETVADYTFPDGLRIDKGMRVHVPVYAIHRNPD

NFPDPEEFRPERYLGDAKNDIKQFTFFPFGEGPRICIGMRFGKMQTIAGLITCLKKFNFELA

DGMPRTLAFRSTTLLTQLSTGLFLKATPRDGWEQRIFAR

>BmorCYP9A20

MILLIWAVVLIAAFVLFYKQAYSLFSKHGVKGFTPLPFFGNMGRIVIKMDHFSDHIQSLYDS

FPEERFVGRYEFLNPMVIIRDIELLKKITVKDFEHFLDHRTIINKDTDPFFGRSLFFLRDQDW

KDMRSTLSPAFTSSKMKLMMPFIVEVGEQMNKALKQRIQEAGVGYVDIDSKDLTTRYAN

DVIASCAFGLKVDSITEENNQFYAMGKAASTFNFRQLLIFFGLASVPKLVKILRITLFQKEIK

TFFRELILGTMKNREAQNIIRPDMIHLLMEAKKGKLRHDEKSTKDSDAGFATVEESSVGK

KDINRVWTDDDLVAQAVLFFVAGFETVSSAMTFLLHELALNPEVQEKLVEEIRENEKNNN

GKFDYNSIQNMVYLDMVVSEVLRLWPPVIALDRMCVKDYNLGKPNDKSKEDFIIRKDVA

VGIPVWGLHRDPEFFPNPLKFDPERFSEENKHNIKPFSYMPFGLGPRNCIGSRFALCEVKV

MTYQLLQHMEISPCEKTCIPSKLSKETFNLRLEGGHWIRLKIRN

>BmorCYP15C1

MLALIVLCFILFFYIISRRHRGLCYPPGPTPLPIVGNLLSVLWESRKFKCHHLIWQSWSQKY

GNLLGLRLGSINVVVVTGIELIREVSNREVFEGRPDGFFYTMRSFGKKLGLVFSDGPTWHR

TRRFVLKYLKNFGYNSRFMNVYIGEECEALVQLRLADAGEPILVNQMFHITIVNILWRLVA

GKRYDLEDQRLKKLCSLVMRLFKLVDMSGGILNFLPFLRHFVPRLIGFTELQEIHNALHQY

LREIIKEHQENLQLGAPKDVIDAFLIDMLESQDDKLTLDDLQVVCLDLLEAGMETVTNTA

VFMLLHVVRNEDVQRKLHQEIDDIIGRDRNHLLDDRIRMVYTEAVILETLRISTVASMGIP

HMALNDAKLGNYIIPKGTFILLSLYELHHGPHWKDPETFRPERFLTKEGNILQDEWLIPFGI

GKRRCIGEGLARSELFMFLTHILQKFHLRIPKNEPLPSTEPIDGLSLSAKQFRIIFEPRKTFKSI

>BmorCYP18A1

MITMLTNSKILWALWQVMNYCVSRTSVMLIIVTCTALLLTQFLKLVRDIRKLPPGPWGPPV

VGYLPFLGVRHKTFLQLARNYGALFSARLGNQLTIVMSDYKIIREAFRREEFTGRPSTPLM

HTLDGLGIINSEGRLWKNQRRFLHEKLREFGMTYMGNGKKLMEDRIKNEIHELIVSLHRA

QGAPIDVNPLLALCVSNVICGITMSVRFSNGDVRFERLNHLIEEGMRLFGEVHYGEYIPLY

NYLPGKALAQEKVAKNRDEMFAFYQTLIDEHRETLDINNARDLIDVYLIEIEKAKSEGRAG

ELFEGRDHELQLKQILGDLFSAGMETIKSSLLWMIVFMLRNPDVKRRVQEELDAVIGRERL

PSIDDISSLPYTETTILETLRLSSIVPLATTHSPTRDVQINGYKIPAGSQVIPLINCVHMDPNL

WDEPNKFNPSRFIDATGKIRRPEYFMPFGVGRRMCLGDVLARKEMFMFFSCMMHQFDLE

MAEGDALPSLEGIVGATIAPKAFRVKFLARSPVPLVPTTLSADSSHLRHVGSH

>BmorCYP9A21

MIIIIWTLAIGLAFLLYLKQIYCYFSKHEIESITPLPILGNMGKIVFKINHFVDDISQLYNKFPE

ERFVGRYEFVNPVIYIRDIEIVKRITIKDFEHFLDHRTIVNEETDPIFGRNLFSLKGQEWKDM

RSTLSPAFTSSKMKLMMPLIVEVGEQMINALKKNIKNSGVGYVDIDTKDLTTRYANDVIAS

CAFGLKVDSLTEENNQFYAMGRAASNFSFKQILLLLGFISFPKMMKMTKFTLFSEETSGFF

KELIMGTMKDREMRKIIRPDMIHLLMEAKKGKLVHDDKSSKDTDAGYATVEESAVGKKQ

IDRVWTDDDIIAQAVLFFVAGFETVSSAMTFLLHELALNPEVQDKLVEEIMENKERNNGKF

DYNSIQNMVYLDMVVSEPLRLWPPGVSMDRICVQDYNLGKPNDKAKRDFILRKGTGVAI

PVWAFHRNPEFFPDPLKFDPERFSEENKHNIKPFAYLPFGVGPRNCIGSRFALCEVKVMAY

QLLQHMEISPCEKTCIPSKLSKETFNLRLEGGHWVRLKIRD

>BmorCYP49A1

MRGTAVMWPLRTVNRSICTTASRQQVSEGSKEMGAKNSMLSTCPVHIQRARSTHVVDAT

AFEVSSPVKPWEDVPGPKPLPLLGNTWRFTPYIGSYSVEHIDRICVSLRAKYGKCVKMAG

LLGRPDMLFVFDANEVERVFRGEDAAPHRPSMPSLNYYKHTLRKDFFSAEENCAGVIAVH

GDSWSAFRTKVSRVALSAGAAAQYTAPVAEVADCFVERIRKIRDENMETPEDFLNEIHKW

SLESLGLIALDTRLGCFEACEGSESQRLIDAVKTFFLCVGELELRAPWWRLYPTAMFRRYV

AALDTILSVTLRHVDKALEEIKLNGSSKSLLQDLVTAAGARVAAVAALDMFLVGIDTTSTA

VASILYQLSSRPHVQEKIYEEVTKALQGRPMSPGDLNQMPYLKATVKEVLRMYPVVIGNG

RQLTKDTVICGYNIPKGTQVIFQHYVMGNSEEYFKDASQFRPERWLKRTAQRHHAFASLP

FGYGKRMCLGRRFAELEIHTVICKMIQAYKIEYRREPLEYHIHPMYTPNGPIRLRLVER

>BmorCYP314A1

MSLPGVFLFSHYVESFWSTSPPLLDWSCVPTLVLAVIAVVVAVTALLTRTSDAKHSCRLPGP

QPLPFLGTRWLFWSRYKMNKLHEAYADMFKRYGPVFMETTPGGVAVVSIAERTALEAVLR

SPAKKPYRPPTEIVQMYRRSRPDRYASTGLVNEQGEKWYHLRRNLTTDLTSPHTMQNFLP

QLNTISDDFLELLNTSRQSDGTVYAFEQLTNRMGLESVCGLMLGSRLGFLERWMSGRAM

PLAAAVKNHFRAQRDSYYGAPLWKFAPTALYKTFVKSEETIHAXANSLVFLLYLLSGRPD

WQRQINSELPPYTMLCSEDLAGAPSVRAAINEAFRLLPTAPFLARLLDSPMTIGGHKIPPGT

FVLAHTAAACRREENFWRAEEYLPERWIKVQEPHAYSLVAPFGRGRRMCPGKRFVELELH

LLLAKIMQKWRVEFDGELDIQFDFLLSAKSPVTLRLVEW

>BmorCYP315A1

MHRFPSMSSIRSAVRSRNSNRCSMSTKPHKSLRTIDEMPHKKSLPIIGTKFDLFSAGGGKNL

HKYIDMRHKQLGPIFYERLTGKTKLVFISDPTHMKSLFLNLEGKYPAHILPEPWVLYEKLY

GSKRGLFFMDGEDWLINRRIMNKHLLREDSDVWLRAPIRTAVFHFICNWKLRAQSGNFSP

NLESEFYRFSTDVILAVLQGNSALLKPTPEYEMLLLLFSEAVKKIFSTTTKLYALPVEFCQR

WNLKVWRNFKQSVDDSISIAQKIVYEMLHTKDAGDGLVKRLKDENMSDELITRIVADFVI

AAGDTTAYTSLWILFLLSNNTEILTEMNDNDQYVKNVVKEAMRLYPVAPFLTRILPKQCVL

GPYLLEEGTPVIASIYTSGRDEQNFSKADQFLPYRWDRNDQRKKDLVNHVPSATLPFAFG

ARSCIGKKMAMLQMTELISQIVKNFDLKSMNNSDVDAVTSQVLVPNKDIKVLILPRSISK

>BmorCYP302A1

MFVRLTVKNNIPYRARKCVYRRASENFVGSEHASKVNEQGDNLMNFEDIPGPRSYPIIGTL

HKYLPLIGDYDAEALDKNAILNWRRYGSLVREKPIVNLVHVYDPDDIEAVFRQDHRYPAR

RSHTAMNYYRTNKPNVYNTGGLLATNGPDWWRLRSIFQKNFTSPQSVKTHVSDTDNIAK

EFVEWIKRDKVSSKNDFLTFLNRLNLEIIGVVAFNERFNSFALSEQDPESRSSKTIAAAFGSN

SGVMKLDKGFLWKMFSTPLYKKLVNSQIYLEKISTDILIRKINLFESDDSKNDKSLLKTFLQ

QPQLDHKDIMGMMVDILMAAIDTTAYTTSFVLYHIARNKRCQDEMFEELHTLLPKKDDEI

TADVLSKASYVRSSIKESLRLNPVSIGIGRWLQKDIVLKGYSIPKGTVIVTQNMTSSRLPQFI

RDPLTFKPERWMRGSPQYETIHPFLSLPFGHGPRSCIARRLAEQNICIILMRLIREFEIQWAG

EELGVKTLLINKPNKPVSLNFIPRSS

>BmorCYP333A2

MFDVLGILEGKFGPLVKVDGVFARGTMLFLFEPDHFEQVYRAEEANPLRPGFQVLDYYRT

QLRKSRYGGLHGLINAQGPEWREFRTKVNPALLLPKLVKLYAPGIDEIAQDFVQRLSQITH

DDAYIKDNFELELTKFSLEATALVALGSRLGCLKDSLDSDHPARRLMKSTRDIFELTYKLEI

RPSPWRYIATPAYKMVIEAYDTQWEISMMYINHARKKLEQRGYDIPEEEKSVLEKLIAIDE

KVAVMMASEMLLAGIDTVSFVTTSLLFNLAMNQKVQDKLREEIRTSESNYKRYFRACLKE

ALRLRHVVPANLRRTHRDHIVGGYYIPKGIDVIAPNEYLSRSEKFYPQPEEFIPERWLVEKS

DPLYYGNAHPLVTLPFGFGVRSCIGRRIAELEIELLIKRLIEEFKVTWNGPPIKIVNKLTNTF

VKPYNFTFTSVK

>BmorCYP333B1

MIIALHYSKLRPVLNFSCLQQCVRTVTVSAATEKLQQTELKSFREIPGPSSLPIMGPFLHFM

PGGSLHNINSTELTHKLYDIYGPIVRIDSMFSKDAIVLLYDAESAGIILRNENNMPIRISFKSL
SYYRQKYKKSENDRTDRPTGLVSDHGELWKSFRSAVNPVLLQPKTIRLYSSALEEVATDM
VERLRSLRDENNRIRGQFDQEMNLWSLESIGVVALGNRLNCFDSNLQDDSPVKRLIECVH
QMFVLSNELDLKPSIWTYVSTPLFRKTMKMYEEQENLTKYFIKKALDDIKMNKSKSDDE
KPVLEKLLDINEEYAYIMASDMLVAGVDTTSNTMSATLYLMAINQDKQQKLREEVMSKN
GKRSYLRACIKEAMRILPVVSGNMRRTTKEYNILGYHIPENVDIAFAHQHLSMMEKYYPR
PTEFIPERWLTNKSDPLYYGNAHPFANSPFGFGVRSCIGRRIAELEVETFLSKIVENFQVEW
SGSSPRVEQTSINYFKGPFNFIFKDL
>BmorCYP306A1
MDLYFIWLVTFVAGFWIFKKIKEWQNLPPGPWGLPIVGYLPFIDRYHPHITLTNLSKTYGAI
YGLKMGSIYAVVLSDHKLVGDTFSKDSFSGRAPLYLTHGLMNGNGIICAEGGLWRDQRKL
ITSWLKSFGMSKHSVSREKLEKRIASGVYEILENIEKTSDAALDLPHMLTNSLGNVVNEIIF
GFKFPPEDKTWQWFRQIQEEGCHEMGVAGVVNFLPFIRHVSPSTRKTIEVLVRGQAQTHT
LYASMIDRRRKMLGLEKPKGAEYAPHENLLKLYPNGHIKCIKYSKVSPNTEHFFDPNTLIPT
EGDCILDNFLLEQKKRFESGDPTALYMRDEQLHFLLADMFGAGLDTTSVTLAWFLLYMA
LFPEEQEEIRKEILSVYPYDDDVDSSRLPLLMAAICETQRIRSIVPVGIPHGCIEDAYLGNYR
IPKNAMVIPLQWAIHMDPNVWEEPEKFKPRRFLAQDGSLLKPQEFIPFQTGKRMCPGDEL
SRMLSCGLVSRLFRKQRIRLASKIPTAEEMRGTVGVTLAPPPVKYYCEPI
>BmorCYP6AE2
MSVSALVFAAFVLLVTYIYYWSTRKFDYWKRKNVPYAKPVPFFGNYMRYITLQSYIGDVT
QKLCQQFPDRPYFGSFYGTEPALVLQNPEIIKQVFTKDFYYFNSRDNSDYNHKEVVTQNLF
FANGDKWKVVRQNLTPLFSSLKMKNVFYLVEKCNHSLEDMLDKEIKDLQIIEIRSAMIRYT
LDSICSSAFGIESNTLREGGENSPFANMGSIVFSSSITRGLKWISRSMWPGIFYKLGLQCFPA
EIDGFFERVLIEVFENRGYKPTNRNDFVDLILSLKQNDYLTGDGLVPKNVDAKKVTVKVD
DALLIAQCVVFFGAGFETSASTLSAALYELAKNPEAQRRAQEEVDELLLKHNNKLNYDCL
AELPYLEACMNEAMRLYPVLPNITREAVTDYTFPDGLRIDKGMRVHVPVYAIHRNPDNFP
DPEEFRPERHLGDAKNDIKQFTYFPFGEGPRICIGMRFGKMQTIAGMVTCLKKYNFELAD
GMSKTVPFRSTTVLTQPSTGLFLKATPRDGWKQRIFAR
>BmorCYP9G3
MLVEVIVFLITTLVAYYLYVYKKIHYFYDARGVKYQPGIPVLGNILKSSLGTGHFWEDIDKI

YKAFPGERYIGYIEGTTPILMIKDPEIIKNITVRDFDHFVNHKEFFPVEIDALFGGSLFMMKD
DKWRDMRTTLSPAFTGSKMRLMLPFMIDISKNIVEYLKGHQLEDVDVDDLMRRYTNDVI
ASAGFGLQVNSLVDKDNEFYECGQAMFSTSWPQRFKMILAAQFPTLAKKIGIKVFPQKVT
RFFREIVTSTMDYRLKNNVERPDMIQLLMDAYKGTLKNESNESDEKNVGFAMTEEMLKP
KGNVRKWTQDELTAQVFIFFLAGFESSANGLTLCIHELALNPEAQEKLYEAIIKFKEEKGPL
TYDNIGELKYLDCVLNETSRKWSAAIIVDRVCSKPYELPPPREGGKPYKLQPGDIVYNSVN
SIQMDPEHHPDPEKFDPDRFLDENKHKIKPFTFLPFGAGPRNCIGSRFALLELKVLIYYIVL
NFKIIKTEKTLSPIKLQPGEFNIKVLGGTWTQFEARE
>BmorCYP337A2
MLPVLVIVSLVAILILYWQSSNYWKKRNVREVNGTVLKFTFGNCSLPEYYKQIYDKYNEN
QIGFHLGASPALVLRDLQDVQAVLASNFQSFYRRGFAVNDADVLGGNMLFLDDLPRWKIL
RQKLSPAFSSLRLKAMYEAIEKTARDFTDYIATDERAIKEPFDAVLKYTTASIGVAVFGLDE
KGESLIDLPLSHVAGNALKPSLKANIVFFIGSTFPRLFKWLNMTFFGEYEDVFIGAVKKVLK
NRRKLDKRLDIVDVYLDMQSSGRLRDVVTGFEMEPTDEVIAAQSFFFYVAGADTTANAIH
FILLELSANPHVLNKLHAEIDTVLPKGTEVLTFEDIDRLTYMDMVISEAMRKYPPIGFIQRL
CTKDAILPSNNLRISKDTVTVVPILAIHRDERFYPNPDVFDPERFTPEKIKERNKFSYLAFGE
GNRICIGARFSRLQVKACITWLLRKYTLKPQEYKPERFERSAFSLRDTKSKYEFIRRTN
>BmorCYP340A2
MFILIVLVVVCVVLLLYSIKKSNSNVPLCDNYLPVIGHTHMLIGDGKKLLRSVKYICGETN
KKGGVARCKLGLDYYYVITDPEDNLTVANGALQKHFFYQFASNWLGDGLVTSSGETWKR
HRKLLSPAFSQQMLNIYTVVFNRESRNLISYIEIQMKSGPVLITAAFKEMALKALLSTAFGI
EVEECDFNQKYMHAVDEVMAVLTRRIQNILLHNSFVYKLSALKKKEDELIETITTMSNKAI
NSKRRALKNKQESHENGCASEKKTQSLSDLLLKGLNDEAFTDKEIRNEVDTFIAAGSHTS
SQLMTVVVMVLGSYPEIQDKVYQEIKSVCGDSDADVDKLQHPRLVYTEAVLKETLRLYPI
APVVFRKTENEIKLKNYTIPANSNCMLGIYGLNHHPVWGPDAHTFRPERWLEPGGVPDDP
NAFAGFSVGKRNCIGKNYALISMKIMLVHLVRRYKVTADISKIEFKMNVLMVPSDNCYVK
FESRK
>HarmCYP6AE12
MITLLLSLVIPLVFFAIYITSKRKYEHWEKKKIPHLKPVPILGNFSEYILQKTYLGQVAQKIC
KKFPNEPFIGAYYGTEPALIVQDPELIKIVMTKDFYFFSGREVSNYSHKEMFTQNLFFTYGD

RWKVLRQNLTPLFSSAKMKNMFHLIEKCSRVFEKVLDKETQKCDEIEVRALMAGFTMDCI

GSCAFGVDTNVMEKTENNIFRKIADLIFDIQGFRAFKNVARAIWPSVFYGLGFRAFPREID

NFFNNLMSGIFKGREYKPTNRNDFVDLVLKLSNNKTMTGDSLLNMKSDADKKVVLEVD

EEMLVAQCVLFFAAGFETSSTTLSYALYELAKNPEAQEKALAEVDEYLRRHDNTLKYECV

AGTPYLEACVDEALRLYPVLGLLTREVAEDYTFPSGLEVEKGLRVHLPVYHLHHHPEHFP

DPEQFRPERFLAENKQNIKPYTYMPFGEGPRICIGMRFARMQMAAGLLTLLKKYRVELAP

GMKRELVFEPGAIVTQPIGGIKLKFVERENWKERLLKTP

>HarmCYP6AE14

MITSLLLTAVFVIIFTIYLVSKKKYQYWEKRKVPHLPPVPLLGNFGNFILQRQFLGYTLQQIC

GKFPNVPYVGAYFGTEPALIVQDPEHIKLVMTKDFYFFSSREISEYADRERFTQNLFSTSGN

KWKVLRQNLTPVFTSAKMKNMFHLIEKCSHVFEDFLDKEAKSNEVEMRALVARYTMDCI

GTCAFGVETKTMNVTENNPFTAVGNSIFMLSRVQGFKFVLRGIYPSLFYLLGFRTLPPEVN

AFFSNLMTGVFKGRNYTPTSRNDFVDFVLKWKQNKTMTGDSLTNMKYDSQKKVTLEVD

DDLLVAQCFIFFAAGYETSATTLSFTLYELAKHPEAQKRAIAEVDDYLRRHNNELKYECLS

EMPFVEACFDETLRKYPVLSLLTREVVEDYTFPSGLKVEKGLRIFLPLYHLHHNPEFFPDPE

EYRPERFLPENKDKIKPYTYMPFGEGPRLCIGMRFAKMQMTAGIITLLKKYRLELAPGMP

QNIEFEPNSFVSQVAGGINLKMIKRESWEGRLLKNLEKAY

>HarmCYP6B2

MWIFYFPAVISVLIVTLYFYFTRTFNYWKKRNVRGPEPVVFFGNLKDSALRKKNMGVVME

ELYNMFPEEKVIGIYRMTSPCLLVRDLEVIKHIMIKDFEVFSDRGLEFSKEGLGQNLFHAD

GDTWRTLRNRFTPIFTSGKLKNMFYLMNEGADNFIDHVSKECEKHQEFEIHTLLQTYTMS

TISSCAFGVSYDTISDKLDTLAIVDKIISEPSYAIELDMMYPGLLPKLNLSIFPSVVHKFFKNL

VNTIVTQRNGKPSGRNDFMDLILELRQMGEITSNKYGNNMSTLEITESVMCAQAFVFYIA

GYETSATTMAYLTYQLALNPDIQNKLIAEIDEAIKANGGKVTYDTVKDMKYLNKVFDETL

RMYSIVEPLQRKAIRDYKLPGTDVVIEKDTVVLISPRGIHYDPKYYDNPKQFNPERFFAEE

VGKRHPCAYLPFGLGQRNCIGMRFGRLQSLLCITKLLSKFRLEPSKNTDRNLQVEPYRFIIG

PKGGIRLNIVPRKDVS

>HarmCYP6B6

MWIFYFPAVISVLIVSLYFYFTRTFNYWKKRNVRGPEPTVFFGNLKDSALPRKNMGVVME

ELYNMFPEEKVIGIYRMTSPCLLVRDLEVIKHIMIKDFEVFSDRGVEFSKEGLGSNLFHADG

ETWRALRNRFTPIFTSGKPKNMFYLMHEGADNFIDHVSAECEKNQEFEVHSLLQTYTMST
IAACAFGISYDSIGDKVKALDIVDKIISEPSYAIELDMMYPGLLSKLNLSIFPTAVKNFFKSLV
DNIVAQRNGKPSGRNDFMDLILELRQLGEVTSNKYGSSASSLEITDEVICAQAFVFYIAGY
ETSATTMAYMIYQLALSPDIQNKLIAEVDEVLKANDGKVTYDTVKEMKYMNKAFDETLR
MYSIVEPLQRKATRDYKIPGTDVVIEKDTIVLISPRGIHYDPKYYDNPKQFNPDRFDAEEV
GKRHPCAYLPFGLGQRNCIGMRFGRLQSLLCITKILSKFRIEPSKNTDRNLQVEPHRGLIGP
KGGIRVNVVPRKLVS
>HarmCYP6B7
MWVLYLPAVLSVLIVTLYLYFTRTFNYWKKRNVRGPEPTVFFGNLKDSTLRKKNIGIVME
EIYNQFPYEKVVGMYRMTTPCLLVRDFHVIKHIMIKDFEAFRDRGVEFSKEGLGQNLFHA
DGETWRALRNRFTPIFTSGKLKNMFYLMHEGADNFIDHVSKECEKKQEFEVHSLLQTYT
MSTISSCAFGVSYNSISDKVQTLEIVDKIISEPSYAIELDYMYPKLLAKLNLSIIPTPVQHFFK
SLVDNIISQRNGKPAGRNDFMDLILELRQMGEVTSNKYLDGVTSLEITDEVICAQAFVFYV
AGYETSATTMSYLIYQLSLNQDVQNKLIAEVDEAIKASDGKVTYDTVKEMKYLNKVFDE
TLRMYSIVEPLQRKATRDYQIPGTDVVIEKDTMVLISPRGIHYDPKYYDNPKQFNPDRFDA
EEVGKRHPCAYLPFGLGQRNCIGMRFGRLQSLLCITKILSKFRIEPSKNTDRNLQVEPRRVT
IGPKGGIRVNIVPRKIVS
>HarmCYP9A12
MILVLVWVAVLITVAVLYLRQIYSRFSRYGVKHFRPVPLLGNMTRMVLKQDHLIEDILRYY
NSFPGERFVGRFEFISEMVVIRDLELVKKIAVKDFEHFLDHRSIFSSSDSFFSRNLFSLKGQE
WKDMRSTLSPAFTSSKMRMMVPFMVEVGDQMMDTIKKKIKESGNGYIDLECKDLTTRYA
NDVIASCAFGLKVDSHNETDNEFYTMGKLSSTFRFREMIMFFVIANAPTVAKILKLDFLSE
AAKKFFRNFVLDTMKNRELNHIIRPDMIHLLMEAKKGKLTHEEIKSNDVTAGFATVEESAV
GQKEITRVWTEEDLIAQAVLFFIAGFETVSSGMSFLLYGLAVNPDVQERLAQEIKENDAKN
GGKFDFNSIQNLQYMDMVVSELLRLWPPGAALDRICTKDYSLGKPNDKAKHDFIVRKGT
GISIPAFAFHRDPQFFPNPEKFDPERFSEENKHNIQSFAYMPFGIGPRNCIGSRFALCEMKVM
AYQILQHMEVSPCERTCIPAKLDTETFNMRLKGGHWLRFRPRQ
>HarmCYP9A14
MIALLWLAVLVAALTLYLRQVYSRFSRFGVKHFEPVPLVGNLSTVLMRKAHASEDFNNLY
QAFPGERFVGRYEFLRNIVMIRDLELVKSITVKDFEHFIDHRMLADADVEPLFGRNLFSLR

GHEWKEMRSTLSPAFTSSKMKAMVPFMMEVSEQLINFLKMQIKESGGKHADIECKDLMT

RYANDVIASCAFGLKVDSHNDRENEFYSIGTETANFDFKKMLVIFGYACFPAIMKKFNVK

MFSELIVNFFKNIVIGTMRNRQKNNILRPDMIHLLMVAKKGKLTHEEKVAEANTGFATVEE

SDIGKVTVKKEWTEDDLTAQAVLFFVAGYETISSAMAFLIYELAVHPEVQEKLAKEIREHD

AKNGGKFDFNSIQNMPYLDMVISEVLRLWPPAVGLDRECSKDYNLGKPNDKAEKDYILR

KGEALVIPVWSIHHDPEYFPDPYKFDPERFSEENKHKIQPFSYMPFGLGPRNCIGSRFALCE

VKVMAYQLIQQMELSPCEKTSIPAVLAKDTFNLKVEGGHYIRVKLRQ

>HarmCYP9A17

MILALVWVAVLIAVAVLYLRQVYSRFSRYGVKQFRPVPILGNMTRILLKQDHFVDDTMRY

YNSFPEERFVGKFEFIKELVVIRDIELAKKIAVKDFEHFLDHRSMFSSSDSFFSRNLFSLKGQ

EWKDMRSTLSPAFTSSKMRMMVPFMVEVGDQMMAAIKNKIKESGNGYIDIECKDLTTRY

ANDVIASCAFGLKVDSHNETDNEFYTMGKLSSTFNFRQMLVFFFIANAPTVAKILKLDFLS

EAAKKFFRNLVLDTMKNRELNHIIRPDMIHLLMEAKKGKLTHEEIKSNDVTAGFATVEESA

VGQKEITRVWTDEDLIAQAVLFFIAGFETVSSGMSFLLYELAVNPDVQERLAQEIKENDAK

NGGKFDFNSIQNLQYMDMVVSELLRLWPPGAALDRICTKDYNLGKPNDKAKHDFIVRKG

TGISIPAFAFHRDPQFFPNPEKFDPERFSEENKHNIQSFAYMPFGIGPRNCIGSRFALCEMKV

MAYQILQHMEVSPCERTCIPAKLDTETFNMRLKGGHWLRFRLRM

>HarmCYP9A18

MTILLLIWALVLAVIVLRDLRRLYSTFSKDGIKHFKPVPLLGNMSSVIFRRNHYADNILKFY

KSFPDEKFVGRFEFVNQSIVILDLELVKKITIKDFEHFPDHRNFGAESFFSRTLFLLAGQEW

KDMRSTLTPAFTSSKIRLMVPFMVEVGDQMILSLKKKLEESKDDYIEVDCKDLTTRYAND

VIASCAFGLKVDSHMDRDNRFYAMGGSLFEFGFRTMIMFFVLLNAPKVAKFFKWDIVPAS

VRKFFTNLVLDTMKDREMRHIIRPEMIHLLMEANKGKLTHEDIMPNDNAVGFATVEESAI

GLNSSKRVWTDEDIVAQAFVFFAAGFETVSATMAFLLYELAVHPEVQERLAQEIREYDDK

NDGKFDFNSIQDMKYMDMVVSEVLRKWPAPLVLDRICTKDYNLGKPNNAAEKDFIIRKG

SGVQIPVYAFHNDPRYFPNPEKFDPERFSPDNKHKFNANAYMPFGVGPRNCIGSRFALCEV

KVITYQILRHMEVSPCKSTCIPAKLATNNLNLRLKGGHSLRFKLRK

>HarmCYP9AJ3

MWWLLVLLVTCLFYYSRNRLKYFSSRGVCTLPPVPFLGNLTAVTFGRENFVEAIAAGYDA

FKDQRYFGLYQYLVPTLIPRDPELIRQIMVRDFNSFADRGVHIDADCDPLFGRNLIMLTGSK

WRSMRASLSPAFSGARCRSMAPLMAESACAVIAYLREKISGEEAMDINKITMSYVNDVIA
SCAFGFAVDSLKDPDNCIFRLGKKAVVQDTNQVMKFFGYENMKSIMKFLQVKIIPTQDAE
QFSQLFKSALKARRENLVKPRPDFIQILVDAAQGKPKADVEGDANNNEYLKSTEKFTDDD
LVAQAVLFYVAGYDTTANLIYYFLYEMAINPRVQEKLHEELDGLPADEDIEDLYEAVQGLE
YLDMCVNEVLRLWPLVGSADRRSVATYDFGPTYPGSKDRLIAPAAIHVWLPIYSIHRDETY
WPKPHDCIPERFSKDRKADIVPYTYMPFGNGPRHCIGSRFAVLAAKVFLVKFLRSYKTRAD
RASPHLSPRAFILRPRDGYLLYVTPR
>HarmCYP9G5
MIFEIVIFLLTFLLGYYFYSYKQVHNYFKQQDVKFLPGIPIFGNILGTTLTTKHFLDDMTAV
YDAFPDEKYVGFVELTLRVLIIRDPELIKSITIKDFDYFTDHREFFTAEADPLFAGSLLLMKG
DRWREMRTTLSPAFTSSKMKYMLPLILENSQNIIEYLNDHQSQTIEVDDIIRRYTNDVIASA
GFGLKINSLQDRNNEFYRIGTTIFDFGLKQKMIIILNTCFPNLARRLGFHLFPDRTYNFFRNI
VSSTIEYRKREKVERPDMIQLLMEAPREWTADELTSQVFIFFAAGFDTSATALVMTIHEIAL
HPDVQEKLYQECRRHKEEKQLTFDSLSELKYMECVINETLRKWSPAIFMDRTCVKTYELP
PPREGGRPYILKPGDIVYNMVNCLHMDPKYFPEPDKFIPERFSEENKHNIKPCTYAPFGGG
PRICIGMRFAMMEIKVLLYNIILNFNIVKTEKTMDPIKLKPHNFNIKAANGTWVKFQKRE
>HarmCYP9A3
MILLLTWVVVIVSALLLYFRSVYSQLSKQGVNHLPTVPIFGNILWPFLRKEHFIDTLMRAVN
TFPDDKIVGHYDMVTPVFIICDVDAIKRITVKDFEYFVDRRSFTSSFDPFFGRGLLMLRGDE
WKAMRSTMSPAFTSSKMRLMVPFMEEIALEMIRVLQEKMRASGKPYIDVEAKDMMTRY
ANDVIASCAFGLKVNSQESDHEFFVHSQTITKFTFLRILKIIFFRCLPNLVQKLKMSLVPRES
SDYFSSVVLTTMQDREKNNIVRNDLINILMEVKRGQLTHEKDDADTDAGFATVEESHIGK
KQHNYEWTDSDLVAQAALFLFAGFETASTSMSFLLYELAVNPDVQDRLLQEIREHDEKNR
GKIDYNVIQSMTYLDMVVSEGLRLWPPAAIVDRMCVKDYNFGRPHPHATKDLIIREGHGV
GVAPWVFHRNPKYFPEPTKFDPERFSPENRHKILPFTYFPFGLGPRNCIGSRFALCEIKVML
YVLIREMEVYPFEKTCYPPQLSKDGFNMQLQGGTWLRLRVRPDKSSL
>HarmCYP6AB9
MITLAIIVILLISLYLYGTKNFKYWEKRGIKHDKPLPLFGNNIKGYFFYSSMTQVADELYWK
YPNERVVGFFRASEPELLIRDPEIAKRLLSNEFSHFFQRGFTPNKTVFEPMMQNLFFAEGDL
WKLLRQRMTPAFTSGKLKAMFPLIVERAERLKTRALAAAADGKSLDARDLMARYTTDFI

GACGFGLDADTLNDENSPFRQLGINIFKIKPFELVKLVVKEMFPDLCQNVKIWTRVEKDIN
ELVGEIMAKRNNKPSGRGDFIDLMLECKMKGTMVGESIESRKPDGSPETASLEFNDGIIAA
QVFVFFAAGFETSSSATSFTLHQLAYHPEVQKKAQEEVDRILAKHDGKLSYDSIKEMNYL
EMAFKEGLRMFPSLGFLLRQCTRPYTFPEFNMTIDETCKILIPLQSLHNDPKYFPNPEVFRP
ERFSPEEFDSNNKFVYLPFGLGPRACIGERLGLMQSLAGLAAVLSEFTVEPAPETLRYPVV
DPQSSIVQSVQGGLPLMFRPRKKLA
>HarmCYP337B1
MLLSILFTIFIILCSIVLYFKVTNSNYWKKWNVLQISDVSLEFLFSKESLPIIVKRMYDEHDV
PHIGTTSATIPTLIVKNVDDIQAVLAGDFQSFNSRGIISNANDLLADNLLFMNDFHRWKLIR
QKLSPVFTSAKLKNMFYIIERCARDFVELVEHNAHLRKVPFNLVSRYTTASISAAVFGIDTQ
VKSTMESPFVELAFRALRPSFIQNVIVVIANTFPRLYKLLQLKLFGEHEEFFVGAVKTVLES
RRHDTTKRYDFIEICLELQKNGIMQDFSTGYKLEPTDELMAAQAFFFFVAGADTSANTMH
YSLLELSNNPKILAKLHEEIDKVFEGGAGELTYNDIDKLQYLDQVVNEAMRKYPPVGVM
QRLCTKDTVLPSGIPIAKGNTIMIPVFGLHRDEKYFDDPHVFDPDRFSPENVSKIKNYAYLP
FGEGNRICIGVRFARLQVKAGLAWLLRRFTLVEQDNCIPEFEKSPFGLRSPDARYELKLRDI
>HarmCYP337B3
MLFVILLTILIVLCSIVLHFKFTNSKYWKKKNIIQIKDLTFTFLFNKQSMPELIKSIYDKHDEP
YIGTTSATIPTLIIKNLDDIQAVLAGDFQSFYSRGIITSPNDVLADNLLLMDDFKKWKLIRQK
LTPIFTSAKLKNMFYIIEKSARDFVELVEDNVHLRKKPFKLMTRYTTASISAAVFGIDTQVK
STMESPFVELAFRALRPSFIQNVIVVIANTFPRLYKLLQLKLFGEHEEFFVGAVKTVLESRR
HDTTKRYDFIEICLELQKNGIMQDFSTGYKLEPTDELMAAQAFFFFVAGADTSANTMHYS
LLELSNNPKILAKLHEEIDKVFEGGAGELTYNDIDKLQYLDQVINEAMRKYPPVGVMQRL
CTKDTVLPSGIPIAKGNTIMIPVFGLHRDEKYFDDPHVFDPDRFSPENVSKIKNYAYLPFGE
GNRICIGVRFARLQVKAGLAWLLRRFTLVEQDNCIPEFEKSPFGLRSPDARYELKLRDI
>HarmCYP4S1
MLLSLALVGFITFVLWRLLAEEENPLDKLPGPKKLPIVGSAFEFMRMNPRDLFIKVRLFAEI
YGDRYLIKILGRRILHVHNVKDVEVVLAHSRNIKKSKPYTFLESWLGTGLLLSNGAKWHK
RRKILTPTFHFNILKSFSIVMKERSKGLVEKIKTLENTDVNLLPLISDYTLFIICETAMGTQLD
SDKSAKTQEYKSAILSIGNLLFARLTRVWLHNEYFFKMHSLGRRFQKCLEKVHSFADDVI
MERKKNWKPGQSEFTEEDSVGGKKRLAMLDLLLEAESKGEIDLEGIREEVNTFMFEGHD

TTAMAIVLGLMLIADHEEVQDRIFEECQKIFPDAESTPSMSDLAEMKYLEAVIKETLRLYPS

VPFIARAITEDFMLDDLLVKKGSEVSIHIYDLHRRADLFPEPEAFKPERFLSGEAMHPYAFV

PFSAGPRNCIGQRFAMLEMKCVLSGICRNFKLQPRVKGARPALLADMLIRPAEPIYVKFIRR

>HarmCYP321A1v1

MLQLPLILFGLVLLLTWYLIGRHNENYWKARGVKFYSKNKVIGPSWDYLFTKGAMFEKF

GELYKAYRNEPVVAIGQILTPSLFVIDAKNVQHVLSSDFQSFNHRGLDSVEGDQLTESITLL

NGPKWKLMRKTMTPLFTSSKLKNMYYIMDKCAQDFVTYLKRDPKLWKGNFFDSAMLY

CNAAVCAAIFGIGEQSTFDSPFLKFAKDVSISSFKNNMKFTFFTLAPKLFTMLGLKVFKEHE

DFFVGAIGDVIKQREEENVKRHDFADLCVQLQKNGTLKDQATGYELEPTTGLLSAQAFFF

FTAGVEPAADGIFATFALLSQHPDILRKVHQEIDEYFEKYDGKITYDVVSEMEYVDKVLSE

SLRMFPPIGFLTRQCIHDTVLPVGNVKVAKGTKLFTPIYEIHHDPKYYPDPEVFDPERFSKE

RRPNDDLYMPFGMGNRTCIGARYSKLQLLAAIVHVLRSFTLKPIPESQQKKVTFLRHAIGV

RIGNVKVELIPRDTK

>HarmCYP321A1v2

MLQLPLILFGLVLLLTWYLIGRHNENYWKARGVKFYSKNKVIGPSWDYLFTKGAMFEKF

GELYKAYRNEPVVAIGQILTPSLFVIDAKNVQHVLSSDFQSFNHRGLDSVEGDQLTESITLL

NGPKWKLMRKTTTPLFTSSKLKNMYYIMDKCAQDFVTYLKRDPKLWKGNFFDSAMLYC

NAAVCAAIFGIGEQSTFDSPFLKFAKDVSISSFKNNMKFTFFTLAPKLFTMLGLKVFKEHED

FFVGAIGDVIKQREEENVKRHDFADLCVQLQKNGTLKDQATGYELEPTTGLLSAQAFFFF

TAGVEPAADGIFATFALLSQHPEILQKVHQEIDEYFEKYDGKITYDVVCDMEYVDKVLSES

LRMFPPIGFLTRQCIHDTVLPVGNVKVAKGTKLFTPIYEIHHDPKYYPDPEVFDPERFSKER

RSNDDLYMPFGMGNRTCIGARYSKLQLLAAIVHVLRSFTLKPIPESQQKKVTFLRHAIGVR

IGNVKVELIPRDTK

>HarmCYP321A1v3

MLQLPLILFGLVLLLTWYLIGRHNENYWKARGVKFYSKNKVIGPSWDYLFTKGAMFEKF

GELYKAYRNEPVVAIGQILTPSLFVIDAKNVQHVLSSDFQSFNHRGLDSVEGDQLTESITLL

NGPKWKLMRKTMTPLFTSSKLKNMYYIMDKCAQDFVTYLKRDPKLWKGNFFDSAMLY

CNAAVCAAIFGIGEQSTFDSPFLKFAKDVSISSFKNNMKFTFFTLAPKLFTMLGLKVFKEHE

DFFVGAIGDVIKQREEENVKRHDFADLCVQLQKNGTLKDQATGYELEPTTGLLSAQAFFF

FTAGVEPAADGIFATFALLSQHPEILQKVHQEIDEYFEKYDGKITYDVVSEMEYVDKVLSE

SLRMFPPIGFLTRQCIHDTVLPVGNVKVAKGTKLFTPIYEIHHDPKYYPDPEVFDPERFSKE
RRPNDDLYMPFGMGNRTCIGARYSKLQLLAAIVHVLRSFTLKPIPESQQKKVTFLRHAIGV
RIGNVKVELIPRDTK

>HarmCYP321A1v4

MLQLPLILFGLVLLLTWYLIGRHNENYWKARGVKFYSKNKVIGPSWDYLFTKGAMFEKF
GELYKAYRNEPVVAIGQILTPSLFVIDAKNVQHVLSSDFQSFNHRGLDSVEGDQLTESITLL
NGPKWKLMRKTMTPLFTSSKLKNMYYIMDKCAQDFVTYLKRDPKLWKGNFFDSAMLY
CNAAVCAAIFGIGEQSTFDSPFLKFAKDVSISSFKNNMKFTFFTLAPKLFTMLRLKVFKEHE
DFFVGAIGDVIKQREEENVKRHDFADLCVQLQKNGTLKDQATGYELEPTTGLLSAQAFFF
FTAGVEPAADGIFATFALLSQHPEILQKVHQEIDEYFEKYDGKITYDVVSEMEYVDKVLSE
SLRMFPPIGFLTRQCIHDTVLPVGNVKVAKGTKLFTPIYEIHHDPKYYPDPEVFDPERFSKE
RRPNDDLYMPFGMGNRTCIGARYSKLQLLAAIVHVLRSFTLKPIPENQQKKVTFLRHAIGV
RIGNVKVELIPRDTK

>HarmCYP321A1v5

MLQLPLILFGLVLLLTWYLIGRHNENYWKARGVKFYSKNKVIGPSWDYLFTKGAMFEKF
GELYKAYGNEPVVAIGQILTPSLFVIDAKNVQHVLSSDFQSFNHRGLDSVEGDQLTESITLL
NGPKWKLMRKTMTPLFTSSKLKNMYYIMDKCAQDFVTYLKRDPKLWKGNFFDSAMLY
CNAAVCAAIFGIGEQSTFDSPFLKFAKDVSISSFKNNMKFTFFTLAPKLFTMLGLKVFKEHE
DFFVGAIGDVIKQREEENVKRHDFADLCVQLQKNGTLKDQATGYELEPTTGLLSAQAFFF
FTAGVEPAADGIFATFALLSQHPEILQKVHQEIDEYFEKYDGKITYDVVSEMEYVDKVLSE
SLRMFPPIGFLTRQCIHDTVLPVGNVKVAKGTKLFTPIYEIHHDPKYYPDPEVFDPERFSKE
RRPNDDLYMPFGMGNRTCIGARYSKLQLLAAIVHVLRSFTLKPIPENQQKKVTFLRHAIGV
RIGNVKVELIPRDTK

>SfruCYPCY321A8

MLFLPLSLIALGLLLAWYLIGKRNESYWKKRGVKFYSKNKVIGPYWDYFITQRALFEVLT
DLYKKYRHEPAIGIGQVLTPALFVIDPKNVQQVLSSDFQSFNHRGIESIEGDQLTDNILMMN
GPRWKLMRQNMTPLFTANKLKNMYYIMDKSAQDFANYLKNNPKTRDGNLFETLMMFC
NAAVCGAIFGIGSESIFDSPFLKLAKNISQSNFKMRMKFVIYSLSPKLYKLLRLQAFNEIEDF
FIGSISQVMKSREQENVKRHDFAEIAVAIQKNGIMKDRTTGCEIEPTTGILSAQAFFFFSAGV
EPCANAIFSTLFLLSSHPEILERVHQEIDEHFEKHSNNINYDVICEMKYTDKVLSEAMRMLP

PIGHLTRQCVQNTVLPVGNIPVEKGTKMFTPIYAIHHDPELYPDPEVFDPERFANDRKPND
NIYMPFGMGNRACIGERYAKLQVQAGLVHVLRNFTVKPQKHVKVTFAQDPINVRPTNCD
VQLIPRNIK

>SfruCYP321A9

MVFLPLALVLLTLVLAWYFIGKYNENYWKKRGVAFYEKNKVVGPMWDFMTSQKALFEIF
GKLYKEYRKEPAIGIGQILTPSLFVIDPKNVQQVLSGDFQSFNHRGIESVEGDTLSDNITFM
NGPRWKLMRSNMTPLFTANKLKNMYYIMDKSAQDFVQYLKNNPKTRDGDLFETLMLFC
NAAVCGAIFGIGSESIFDTPFLKLAKNLSQSNILVRMKFIFYSLSPKIYTALGFKLFKEHEEFF
VKSIGEVLKRRDQENVKRHDFADIALAIQKNGTMRDRTTGYEIEPTTGILSAQALFFFTAG
VEPCANAIYATLYLLSSHPEILEKVHQEIDEHFEKHSNNITYDVICDLEYTEKVLSEALRIFP
PIGFLTRQCVQNTVLPVGNIPVEKGTKIFTPIYDIHHDPELYPDPEVFDPERFSKDRRPNDDI
YMPFGMGNRTCIGARYAKLQVQAGLVHVLRNYTVKLSKTSGKLRFAHDPLNVRLRNVGF
ELIPRAIK

>SfruCYP321B1

MLATIILAVAIVVLILTYLRGRYNENYWRKRGTVFYKRNKTFGPLLEFMTGERPLFQVFNE
LYWRYENEPVVGMGSLDSPVLYVKDPTNIQFITQTNFQNFSHRGITVLEEDYLANNVLFL
HGPKWKVIRQKLTPIFTTTKLKNMFYIIDKSAQDFVEHLKQHPEKLKGDTYEHLSHFCTAA
LAAAVFGITTKSTFDSPFREMARQALTPTLFSNIRFAINSVSDKLFAALRLKFFKDQEGFFIG
AIKEVLKQRRTDSTVRHDFIDLCIDLQKSGPMVDNESGYSVEPTDEVMAAQAFFFFLAGID
PTTAGMFGPLYELGRHPEIQKRVQDEVDAIFEKYGGKLTYDAVLELKFTECVLNEGLRLHP
PIGFNTRQCVEDSVLPVGNVPVEKGTKIFMPFYELHHDPKYFPEPDRFDPDRFARGEVSD
MTYMPFGIGKRLCIGARYARMQILTAIAHILRNYTLVTHIKEGGVRYRKEQFQVRLDNCDI
ELVPRQL

>SfruCYP6B50

MFIYLPTLITFVCAIYLYFTRTFDFWKKRNVKAPNPIPFFGNYIDVFFRRKHMGVLYHDIYE
QYPDEKVVGLYRMMSPTLLIRDLDIVKQVLQKDFESFPDRGVYYSKDKLGNNLFHADVD
VMKGIRKHLTTVFTPNKFKSNFYMLANRADQFLEYMGRISDENGEVNMLPALRKYGADS
IMMTAFGIDLNPYDENNLICDVLDEEIQYPSYFLELELLFPGSLARFDLSIFPTKIAQYFKQII
EAGATLGVTEKTERNRAIDIMMELKRQGTITSRKGDDEKENWVEITDELLAGQAFIFYLA
GYGNNCLLGSYALYYLAKNPEKQDILIQEIDEVILKHDGKFSYEALKEMKYLEMVFEETL

RLNPMTNAVVRNAARDVQLEGTDIIVPKNTILAISPYSIQHDEKYFPEPEKFKPERFSAENA

RENHPCAMLSFGLGPRSCLGQKFAQIQFGICMVKILLKYRVECTKNTPESITYTPLRLLLTP

NERIYLRLVNRDK

>SfruCYP6AB12

MIFIAIAAVLLITLYIYGTRNFNYWEKRGVKHDKPYFLIGNNYKEYFGKASMTQVADELY

WRYPNEKVVGFFRSTRPELLIRDPEIAKRILITDFAHFFERGLNPHNTVFEPLMQNLFFAEG

DLWKLLRQRMTPAFTTGKLKAMFPLIVERAEKLKTRALAAAAEGKSLDARDLMARYTT

DFIGACGFGLEADSLNDENSPFRQLGIKIFKVRPQEIMTAIAKELFPELCRNLKIWTRVEKEI

NELVVEILRKRDYKPCGRGDFLDLMLECKQKGTMIGESIQNKKPDGSAETVTLEFNDSLIA

AQVFVFFAAGFETSSSATSFTLHQLAYHQDVQKKAQEEIDRVLAKHGGKLSYDAIKEMHY

LENVFKEGMRMFPSLGFLYRQCTKGFTFPELNFSIDKSVLVLIPLQSMHNDPKYFPEPEVFR

PERFDPDEFDSNNKYVYMPFGSGPRACIGERLGAMQSLAGLAAILSCFTVEPAKETLRHPV

VDPHSSVVQCIRAGLPLMFRERSKRVN

>SfruCYP6AE43

MDGLVVLASLLFVIFSLIYYLSYKKHSYWKKKNVPHADPLPILGNYGNYILQKEYPGKMI

QKLCQKFRNEPYFGAYFGTLPVLIVKDPEIIKHVLTKDYYYISGRESSDYYDQEMMTQNM

FFGSGDRWKVLRQNLTPLFSSLKMKNMFYLIEKCTIGFEDMLNADLKMSNVIEVRSTVAK

YTMDCICSCAFGVESNTMAQKENNPFRFMADVIFESSRYRGFKIICRAAWPSIFYGLGFKL

FPKTIDRFFFNLLTGVYESRGYKPSPRNDFVDLLLNFKTEDYITGDCLSNTKTGEENRVKL

KVSDEMLIAQSVMFFSAGFETSATTTSFTLYELAKHPEIQKRAAEEVDEFMRRHNNKLVY

DCVTELPYLEACMYETLRMYPVIGNLTREVMVDYVLPTGLKLDAGVRVHIPVIDMHYNP

EYFPEPYKYNPERFMPGNKENINPNTFFAFGSGPRLCIGMRFAKMQVMSGLITFLKKYKV

ELAEGMATKLEFDPKTFLTQPIDNCISLKLTEREGWEQRNFLRT

>SfruCYP6AE44

MITLYLLLAIPLALYGVYLVSKRKFRYWEQKKVPHLPPKPILGNFSEYILQQKFYGRVEQEI

CNKFPEEPYVGSYLGTEPTLIIQDPEYIKTVMTKDYYFFSGREVSAYCEKEPLTQNLFFTYG

DRWKVLRQNLTPLFSSAKMRNMFHLIEKCARIFENMVDQEVQKGKDIEVRGLTAKFTMD

AIGNCAFGVETQTMVKTDNNPFTRVGDVIFDTARMRALKGVLRSIWPAMFYSFGGKAVP

ADVDSFFFNLMTSIFKGRNYNPTSRNDFVDLLLKFHNNKTVTGDSMRNLKGDSGKKVSL

EVDDEFLIAQCFLFFAAGYETSATTLSYTLYELAKNPEAQELAIQDVDNYLRRNDNVLKYE

CVTELPYVEACVDEALRLYPVLGVITREVIEDYTFPTGLKLEKGLRVHLPVYHMQHNPNY
FPEPEQYRPERFLGEEKKNIKPYTYFPFGEGPRLCIGMRFAKMQITAGIITLLKKYRVELAP
GMSETIQFEPRSVITAPIGGIRLKFIEREGWQQRVFKVPSEA

>SfruCYP4G75

MSYTTAESVVPTSTWTATNLFYVLVVPALILWYAYWRMSRRHLYELAAKLHGPPGLPLLG
NALEFTGGSHDIFKNVIEKSIPFDGESVVKIWIGPRLLVFLYDPRDVELILSSHTHIDKADEY
RFFKPWLGDGLLISTGQKWRSHRKLIAPTFHLNVLKSFIDLFNANSRAVVNKLKKESGEFD
CHDYMSECTVEILLETAMGVSKTTQDQSGFEYAMAVMKMCDILHLRHTKIWLRPDLLFK
FTDYAKTQTKLLDVIHGLTKKVIRRKKEEFNSGKRPTILNDYSTNNAEETTKTTSVEGLSF
GQAAGLKDDLDVDDADVGQKKRLAFLDLLLESSQSGVVISDEEIKEQVDTIMFEGHDTTA
AGSSFFLSMMGIHQDIQDKVIEELDKIFGDSDRPATFQDTLEMKYLERCLMETLRMFPPVPI
IARHLKQDITLPSCGKQVPAGTTVVVATYKLHRRPDVYENPTKFDPDNFLPEKSANRHYY
AFVPFSAGPRSCVGRKYAMLKLKIILSTILRNFRVYSDLKESDFKLQADIILKRAEGFKVRL
QPRKLAKAC

>SfruCYP4L9

MILFLVSVVLVLVLLVSWISLVRESHRFNVNGPMPLPIIGNAHMFISKSTEFLNLLAKYSEQ
YGKAYRVHFLSVPYVIICDAKYAQDVASSQELIFKGGPYSLMTCWLGQGLLTSAGQRWKS
HRKFLTPAFHFNILQNFLPVFCKNQRVLTEKLRGLADGRPIDMFPIVALAALDNVTESIMGT
SVDAQGHESESAYVKSIEEMSAILAMRIQIPILGPDAIFNLTPMKNRQIKALKVLHGHSTKV
IETRRQELKKANITTLDTSNDTGIRNKHAFLDLLLLGEIDGKKIDDESVREEVDTFMFEGH
DTTTSGISYTLYCLSKRRDVQEKVYEELKTIFGDDMERDPTYQELGQMKYLELVLKESMR
LYPPVPLIERRITRDCEVGGLKLVKGTSVVLNIYQIQRQPDMFEDPLEFRPERFEEALKNPF
SFLAFSAGPRNCIGQKFAMMELKITISEIVKHFYILPVDETPQLSADLILRSKNGINVKFMPR
K

>SfruCYP9F1

MILELLIFVITVLVAYYFYSSWKINNYFKQRDVKYIPGVPFFGNIFYSTFLIRHFVDDLQSVY
DAFPDEKYVGFLENLAPVMLIKDPELIKSITIKDFDHFTDHKEFFTTDSDPLFAGSLLMMK
GDKWRQMRTTLSPAFTGSKMKMMLPLIVDSADNIVEYLNDHQTEDVDVDDLMRRYTSD
VIATAAFGLKVNSLKDRDNEFYRVGSSLFEFTLTQRLLMFSTLFPSLSKKLGTRLFPETTYN
FFRTIVSSTLEYRKREKVERPDMIQLLMETPIEWTPDELTGQVFIFFAAGFETSASGLTMAIH

ELALHPDVQERLYQECSKFKNDKELTFDKLSQLKYLECVINETLRIWSPAIFMDRTCVKAY

ELPPPREGGKPCIVKPGEVVYNMVNCLHMDPKYFPEPKVFNPDRFSDENKHNIQPCTFAPF

GGGPRICIGVRFAMMEIKVLLHHIILNFKIVKTKKTADPIKLKPHVFNIRTMTGTWVRFEKR

Q

>SfruCYP337B5

MLFTLVFTIIILCYVLIHRNFYNKSYWKKKNVVQTNFNLMLKFVFGKQSMPEILRDIYNDH

PDVLYVGTIAGTTPTLLLKHPDDVQAVLAGDFQSFHSRGIKFGPHDLLADNLLFMNDVKR

WKLLRQKLSPVFTTFKLKYMFKIIEKVARDFVEVVEDDIHLRKDPFNILYTYTTASIGASVF

GIDTQNNRNAMDSPFLEMTQKTLKPSLYSNIAALLANTSPRLYDFLNIKVFGEHEEFFIGAV

RAVLEERQRKSIRTHDFIDLCLGLQLHGFMHDFSTNYSIEPTVEVLAAQAFFFFLAGTDTSA

NTMHYTLLELSNNPEVLVKVHEEIDRVFDECNEEFTYNEVEKLQYLDQCVNEAMRKYPTI

GVIQRLCTKDTVLPSGVAIEKDEIVMISPFAMHRDEKYFPEPDKFDPDRFSPENVSKIPKYV

FLPFGEGNRICIGVRFARLQVKAGLAWLLRRFTLREQNCTPTFERSPFGLRSPTDKYELKL

RD

>SfruCYP9A32

MLLLLTWVVVVIITAVVLYLRQTYSYLRRDGINHLPTVPFFGNLFWVMFQKEHFVDLIAKV

TNAFPDDKIVGNYDMMTPILIIRDLEMLKRVTVKDFEHFIDRRSFTSELDPMFGRGLLLLH

GDEWKAMRSTMSPAFTSSKIRLMVPFMEEVCLDMIKVLKQRIKDSGTSYYDMECKEVMT

RYANDVIASCAFGLKVESLTADSEFYVNSKAITKFTFWRFMKVMFYRLFPSVAGMMQLSL

VPKETTDYFTNVVLGTMKNREQNKIVRNDMINILMEVKKGQLTHEKEGKDADAGFATVE

ESHIGKKPHNYEWTDTDLVAQAALFLFAGFDTISTAMSFILYELALNQDVQERLVREIREY

DAKNNGKLDYNSIQNMTYLDMVVSEGMRLWPAAPFMDRVCVNDYNIGRPNKEATKDLII

RKGQCIVIPAYALHRDPEYFPNPTKFDPERFSHENRDKIVPFTYLPFGLGPRNCIGSRFALCE

VKVMLYLLLRDLKLTTCDKTPVPARLSKNGFEMLIYGGAWVRLSVRNE

>SfruCYP6AN4

MFLLLLPLVLILLYHYVTRNHDYWEKRNVKYEKPVPIFGTIYPNLVAKKSITEIAVELYNKY

PNEPAVGIYRGTTPELIVRDLDVVRRILNVDFAYFYPRGIGRNPKEEPVFLNLFHVDGDIWK

LLRQRLTPTFTTAKLKNMFPLVVQCAEKLQTVGEDIVNRGGDCDVRELMARFTTEFIGAC

GFGIQMDSINNEHSLFRALGRNIFHRSWRNYVAVALYDLFPEFRTIIQKSLRETKIMDAIAEI

VENIRKQRNYKPIGRNDFIDLLLELESKGKIVGESVEKRDANGKPEQVEMEMDLTCMVAQ

VFVFFAAGFETSSSATSFMLHQLAYHPEEQRKIQENIDQVLAKYNNKLCYDSISEMTALSN
GFKEAMRLFPSLGTLHRVCAQKYTIPEMGITLDPGVKIIVPVQAIQVDGKYFENPTEFLPD
RFNDTSADRHKFAYLPFGEGPRQCIGARLGEMQSLAGLAAVLHKFSVEPAANTKRHLEVN
HGSNTVQSIRGGLPLKLRLRSKQSHVAA

>SfruCYP9A58

MIIILIWVAVLIAVVVLYLRQLYSTFSRHGVKHFKPFPLLGNMNKIIFRMKHMAFDLQDLYN
AYPEEKFVGRFEFMNEAVLIRDLELVKKVCVKDFEHFVDHRSFGDDSFFGRTLFLLRGQE
WKDMRSTLSPAFTSSKIRLMVPFMVEVGDQMIVSLKKKIAESKGGYIDIDCKDLTTRYAN
DVIASCAFGLKVDSQTEQNNEFYVMGKAATNFNFLQILKFFLILNIPRVTKMFKLDLVTKS
VQKFFTNLVLDTMKEREIKNIVRPDMIHLLIEAKKGKLSHDDVKSNDDATGFATVEESSVG
LRNVVNREWSDNDLVAQAFLFFVAGFESVSSSVCFLLYELAINPDVQERLAQEIREYDAKN
GGKFDFNSIQSMKYMDMVVSELLRRWPTAVATDRICQKDYNIGKPNATAEKDFIVRKGTA
IMIAAFAFHHDPQYFPEPEKFDPERFSDENKHKINPTAYMPFGVGPRNCIGSRFALCEMKVI
TYQILRHFELSPCEKTCIPPRLATDNINLRLQGGHWLRFKLRK

>SfruCYP9A59

MILTLIWVVVVLIAAAVLFLRQAYATFDRYEIKYFKAVPILSDMVTMIRPKEHIGLDLARTYN
SFPNERFVGKLQFLKETIIVRDPELIKKMTVKDYEYFLNHPNTGPPESIFSKFLLFMKEWKE
MRSTLSPAFTSSKIRLMVPFMEEVGDQMILSLKKKIENSKDGYVDIECKDLTTRYANDVIA
TCAFGLKVDSHNDENNQFYSMGKILSAFSLSKILLYMILTNVPYVMEILDWDFIPKSAQRY
FKNLVLDTMKNRELQNIVRPDMIHLLMEAKKGKLKHEDEQISDEGAGFATVEESSVGKK
ALTNKVWTDDDLVAQAFLFFIAGFESVSSSMSFLLHELALNPDVQERLVQEIKEHDEKNG
GKVDFTSIQSMKYMDMVVSEVLRLWPPFVMFDRECTRDYNLGKPNDSASREFVVRKGV
ELWIPTYAIHRDPQYFPDPDKFDPERFSDENKHKIKPFTYTPFGTGPRNCIGSRFALCELKV
MAYQLLRHVELSPCEKTCIPAKLSTDTFNLKLEGGHWLRYRLRK

>SfruCYP9A60

MFYLILVAIFVCVLALYLNYVCSTFNQYGIKHDATVPFIGPTIKLLRAKEHIGQDLKNLYDR
YPEERFLGRFELLKDSIVIRDIELLKKITIKDSEHFLDHTSNGFESLFERSLFFMQGQEWKD
MRSTLSPAFTSSKIRQMVPFMVEVGDQMMTSLKNQIENSKDGYVDIDCKDLTTRYANDVI
ASCAFGLKVNSHTDKNNDFYVTGKTAFEFKLHLLLVFFLISKVPYLKEALNWSVLPKKTQ
DYFINLFMSTMRERELKKIVRPDMIHLLMEANKGKLSHEEVKSTDTAAGFATVEESSFGQ

RTMNRVWKDEDLVAQAFLFFVAGFETVSSSMCFIMHELAVHPDVQDRLVQEIREHDAKN
GGKFTFDSILNMKYMDMVVSEVLRLWPPNVVLDRVCTKDYNMGKPNPAAKKDFIIRKGS
PIMIPSYAFHHDAEFFPEPEKFDPERFSEENKHKINPMAYMPFGVGPRNCIGSRFALCELKV
MLYQLLREMELSPCEKTCIPAKLDTKSFNNRLVGGHWLRFKPRN
>SfruCYP321A7
MLFLPLSLIAVGVLLAWYLIGKRNESYWKKRGVKFYSKNKVAGPFWDFLTSQRALFEILG
DLYKQHRQEPAIGIGQFLTPALFVIDPKNVQQVLSGDFQSFHHRGLETIEGDQLSDNIIMMN
GPRWKLMRQNMTPLFTANKLKNMYYILDKSAQDFVQYLKNNPKTRDGNFFETLMLFCN
AAVCGAIFGIGTNSIFDTPFLKVAKSLSQSNLSIRLKFILANLSPKLFKLLGLKVFNEYEDFFI
GSISKVMKAREQEKVKRHDFADIALAIQKNGTMRDRTTGYEIEPTTGILSAQALFFFTAGV
EPCANAIFSTLILLSSHPEILERVHQEIDEHFEKYSNNITYDVICEMKYIDKVLSEAMRIFPPI
GQLTRQCVQNTVLPVGNIPVEKGTKMFTPIYAIHHDPELYPDPEVFDPERFSKDRRPNDDI
YMPFGMGNRTCIGERYAKLQVQAGLVHVLRNFTVKPQKHQEVIFAHDALNVRPRNVDV
QLIPRNI
>SfruCYP321A10
MVLLPITLALITLVLVWYFIGIYNENYWRKRGVVFYSKNKVLGPLWDFLVSPRPLFQVFGD
LYQQYRHEPVIGLGHLLTPAMFVINQKNVQQVLSGDFQSFNHRGMESIEGDQLSDNITFM
TGPRWKLMRSNMTPLFTANKLKNMYYIMDKSAQDFVQYLKNNPKTRDGNLFETFMMFC
NAAVCGAIFGIGSESIFDSPFLKVARGISQSNLSMKLKFVSFIVSPKLSSLLGLKFFKDYEEFF
VKSIGEVLKRRDQENVKRHDFADIALAIQKNGTMRDRTTGYEIEPTTGILSAQALFFFAAG
VEPCANAIYATLYLLSVHPEILEDVHQEIDKHFEKYNNNITYDVICEMEYTEKVLSEAMRM
FPPIGFLTRQCVQDTVLPVGNIPVQKGTKIFTPIYDIHHDPELYPDPEVFDPERFSKDRRPND
DIYMPFGMGNRTCIGARYAKLQVQAGLVHVLRNFTVASKQTDEKVTFKHDSFNVRLKNV
DVQLIPRNVK
>SfruCYP340L1
MGVLVLALVFCVVFLTWCWRPRRRSPPKYPGALPVIGHVPKILKNLNDLWTYFRKICDY
MVEHDGLISLQVGPHSIYVVSDPDDASVIANTCLEKSYHYKFSNNFVKNGLVTSDSATWK
VHRKILNPAFSPLVLNTYVDEMNVQAQHLVSKLTLYAEKGPIDVKGFITESVLRLICRTSLG
LKPEDQETIDTDYAKAIDEISRVYNERGFNVWLYPSFMYDLSALKRKEQELITRIENIINPVI
QKRRSDLKGKYITNDDASTDSVKGKFKPLLDLLLHLADEEHIFSDTEIREHLDTFVLAAND

TTSAVLRNTLLMLGTYPDVQERLYKEVKEVLHNEDLGKHDLTKLVYSEAVIKEVLRLHPP

VPFVARDLQSDVVLPKYTLRAGNTCALSLYDLNRHSSWGPDAKEFNPDRWLNPDTLPQN

PYAFAGFSIGKRNCIGKKYAMMTLKTSLAHIVRKFHISGDITDLKWKIEVVLKPTTPALVTL

TLRS

>SfruCYP305A1

MIPTFLLLFVVTLILYGFISTIKPRKYPPGPVWFPFIGSSGILQKMTKKYGSEWRACLELSRQ

YSTNVLGMRLSTELLVVVFGEKNVRQVFNDKEFDDRPDNFFARLRCLGYKNKGITFANG

EVWKEHRQFAVKNLKHVGYGKTLMEKEIQNELSSLLKQIKENNDKPINIVNLLSESVINVL

WKFVAGERIKEEKLKHLLDLFRQRGKAFSVAGGMLNQMPWCRFIIPELSGYSLIKKLNGEI

SQVIEEAIEKHKNKEVEGHDFIYKFLNEINANKNATFTEEQLKIVCLDFFIAGSQTSSNFLGF

AFLKVLKSQDIQEKIFNEIDAVIGDRPPCWNDNERLVYTSAFIQEVHRYYPIVCMAGPRSL

QADTSIDGYRIPKGATVLMSLADIYFDPELWDDPNVFRPERFIDESGMLKNSEHIYPFGSGR

RRCPGDSLAKSFIFITFVGILQKYRIHVSNGTVPSDIPVIGILSSPKPYTAEFTLRK

>SfruCYP49A1

MVAPTASSVVLDLGAEPKAIDKSSAMSNARCYAVMPGPRALPIIGNSWRFAIGKNPWKTR

ALDVSLWFLRALAGDGGAAKVSKLFGHPDLVFPFCAEETARIYRREDLMPHRAKAPCLSH

YKQNLRKDFFGDEPGLIGVHGEPWSRFRSKVSKALIAPEAARAAVPDLEHVTDDFVHRM

ECILDDNRELPKDFLTELYKWALESVGAWALGTRLGCLKDNDAEAKEIIRCIHGFFHSVPV

LELSAPLWRIYSTPAYKTYIEALDAFRTLCLKRLTDKGICSQIAKASGEKVATILALDLLLVG

VDTTAAAAASTLYLLANNERAQRKLQTEMDKNLPRDRGMTSKDLDRMPYLKACIKESLR

IKPVILGNGRCIQSDAVISGYSVPKGSHVVFPHYILSNEERYFPSPKEYVPERWLRDNEVQA

PDQAPSGSIANADKPGCPYTRAMEVCRKQKEVGIHPFASLPFGFGRRMCIGKRFAEAELQ

LLLAKIFQKYNVSWRHGELTYSVTPTYVPNEPLQFTLHKRNGNNTSL

>SfruCYP4L4

MILFLVSVVLVFALLVSWISLVRQSRRFNVDGPSPLPLVGNAHLFVVKSSEFLNLVQRLSEK

YGKVFRVHFFSTPYVVICHPKQAEELVSSIEHITKGRSYSFLTCWLGQGLLTSTGQRWKSH

RKFLTPAFHFNILQNFLPVFCKNQRVLTEKLRGLADGRPIDMFPIIALAALDNVTESIMGVC

METQKDSQSEYVKSIEELSAIVTMRMQIPFFGEDAIFNLLPYKTRQDKALKVLHGQTNRVI

ETRRQELKKAKITALPDSNDIGIKNKHAFLDLLLLAEIDGKKIDDESVREEVDTFMFEGHD

TTTSGIVYTLHCLSKRRDVQEKIYEEMKTIFGNDMNRDPTYHELSQMKYLELVLKESMRL

FPPVPLIERRIMKDCEVGGIKLLKGTSVVVNIYQIQRQPDLYDDPLEFRPERFEAPLKNPFS

WLAFSAGPRNCIGQKFAMMELKITISEIVKHFYILPVAQDPELSADLVLRSKNGVQVKFMP

RK

>CsupCYP6SN3

MTLAIILVTLLSLIILLLLYCLLGNRQYWKQFNVKGPEPSALFGNYKDVILRRVTSFDFHEK

IYNSHPNEKVVGLYRFNTPVLLLRDLDIVQQVFIRDFDSFVNRGIEFGDKLSTNLASSCDNT

WRLLRARFSPIFTSGKLKNMLHLVERCGDQLLEHLDATISSSIECDVVNITKRYAIMSISAC

VFGIDRDMTKPDEKLEEVNKMIFTQYQALELFYVCPELLKKMHFNALPKKISEYFIRLVDE

VINKQRNGVPSNRKDFMDLLLEMKNMTELKGGKKNDGDEITVLPITDEVIAAQAMVFHG

AGMETSATTLAFALHELAKNQDVQEKLFQEIKQQVFEKNNGELSLETLKQLTYMAQVFD

ETLRMYPIIDLQRKVSASSVQIPGTDITLKRDDYIFVPIRGIHYDEKYYPDPYKFDPERFSPE

NVKSRHPCAFLPFGLGPRHCIGARFATVQNRLFLVKFLSHYKVEPPAVTKKFTYNPMKITL

AATDGVPLKITRRNIS

>CsupCYP306A1

MDLIFLWLFIFVVGYWIFRKINQWKNLPPGPWGMPILGYLPFIDPKQPHLTLTKLAKTYGPI

YGLGMGNVYTVVLSDHKLVRDAFAKEAFSGRAPLYLTHGIMRGNGIICAEGALWKDQRK

QVSSWLKSFGMSKHSISRDKLEKRIAAGAHELLENVEKSSGSPVDLVHMLTNSLGNVVNE

IIFGFKFPPDDKTWNWFRQIQEEGCHEMGVAGAVNFLPFVRFISPSTRKTIQVLVRGQAQT

HRLYGSIINRRRKMLDLVTPKGAEYPLHENLFDEHPEGHMKCITYSKYTANEEHFFDPNTL

IATDDECILDNFLKEQKRRFESDKESARFMTDEQLLYLLADMFGAGLDTTSVTLSWFLLY

MALHQDEQDLVRKEILSVYPEEEEIDGSKLPYLMAALCETQRIRSIVPVGIPHGCLQDTYL

GNYRIPKGAMVIPLQWAIHMDSDVWENPEEFKPSRFIGPDGSLLKPQEFIPFQTGKRMCPG

DELSRMLSCGCAARLFRRRRIRLASQPPTPEEMRGTVGVTLSPPAVKFYCDSV

>CsupCYP4AU10

MLVLALIFVCVVVYYIRFRHMRKHLYQISEILPGVGELPLIGHTHWFIGGPEKLLKNIQELA

ELVDSSGGTSKVWIGPSLYFVTLNPDDVQKILENCLEKDGSYKFLQQWLGDGLFVAPVDL

WKVHRRVLLPIFHNKILEDFVPVFGQQASILVKRLEEQVGKPDFDIFKYITSCMLDIVFETA

MGEKMDVQHNPDTPYLRARSCVMSIINMRLFKAWLQPDALFKLTPYAKIQQENIDYTHKF

TDEVVMKKKEQCGSQDQQKEGKKDLLELLLHREIKFTNEQLREHIDSITIAGNDTTALVIA

YALLLLGSHEEAQDIVVDELNSIFGESDRAPTKEDINKMHYLERVIKETMRLYTVVPIIGRR

TQKDIKLTNFTLPAGVGCAVAPFVMHRSKRLWGPTANCFDPDRFLPENSVDRHPSPFWDD
GDEKCSRHDSSSL

>CsupCYP4AU11

MDEMTASLPSPPGLPIIGNATMFIGDAEKMLKNLEKLADLAYQHKGCIKLWLGPKLYVAIG
TPEDAQVVLENCLDKDVVYRFLRPWLGHGLFIAPVGLWKAHRKVLLPVFYNKVVEEYL
GVIGVQANVLIERLSEQTDQKEFDVLKYITACTLDIVFETAMGERMDVQHSPETPYLRAR
HTVMTILNMRLFKVWLQPDCLFNLTPYAKEQKDNIDLTHRFTDEKLRAVLDLLFGREIEFT
DEQLREHIDSITIADRMPFLGIQNVVLGAVERVIKESMRLYTVVPIIARNIDKDTYLPGCGV
TIPAGAGAVVGAFAIHRSKMVWGPNAKDFDPDRFLPERSDKRHPAAFIPFSYGSRNCI

>CsupCYP4G90

MSYAAENLVQASTWTATNMFYMLLVPALVLWYAYWRMSRRHMYELAAKLNGPPGLPIIG
NALEFTGGSADIFKNLVSKSVPYDKEGIVKIWIGPRLLVFLYDPRDVELILSSHVYIDKADE
YRFFKPWLGDGLLISTGQKWRSHRKLIAPTFHLNVLKSFIDLFNANSRDVVDKLKKESGA
FDCHDYMSECTVEILLETAMGVSKSTQDQSGFEYAMAVMKMCDILHLRHTKIWLRPEFLF
NFTQYAKNQTKLLDVIHGLTKKVIKRKKEEFKTGKKPSVLSAPETTTSTEIPATKTTSVEGL
SFGQSAGLKDDLDVDDNDVGQKKRLAFLDLLLESSQSGVVISDEEIKEQVDTIMFEGHDT
TAAGSSFFLSMMGIHQDIQDKVIAELDQIFGDSDRPATFQDTLEMKYLERCLMETLRMYPP
VPIIARQLNQDITLPSNGKQVPAGTTVIIATYKLHRRPDIYPNPTKFDPDNFLPEKSANRHYY
AFVPFSAGPRSCVGRKYAMLKLKIILSTILRNFRVQSDLKESDFNLQADIILKRAEGFKVRL
QPRKRMAKA

>CsupCYP4G91

MLEETSGTRYSTARLIFYPLVALVTFLWIYHRWQKQTKFVKMGNKLPGPDPLPFLGNVHL
ALGKNPSDIMKLALEMAQLYLSVARIWVGYKLLVFLAEPDDVEVILNSHVHIDKSDEYRF
FKPWLGDGLLISTGQKWRSHRKIIAPTFHINILKSFVGVFNKNSKDVVEKLRAEVGNVFD
VHEYMGRVTVDILLETAMGITKETQDNAGFDYATAVMKMCDILHQRHYKFWLRLDSMF
KLSSLYKQQVKLLGIIHGLTNKVIREKKKIYLENKARGIIPPTLEELTRTRNYDGDINDTKSL
ADTVFQGYKDDLDFIDENDVGEKKRLAFLDLMLESAQNKTSNITDREIKEQVDTIMFEGH
DTTAAGSSFVLSLLGVHQDIQDKVYDELYEIFGDSDRPVTFADTLNMKYLERVILESLRLY
PPVPIIARKLKHNVKIVTNNYVLPAGTTVVIGTFKVHRNSLYYKDPEKFNPDNFLPENTQN
RHYYSFIPFSAGPRSCVGRKYAMLKLKILLATILRNYKTISVVPVDRSSAVDDVYLS

>CsupCYP4L27

MLVLLSVVLVLLVLLLSWFYFYLDKKCIDVKGPPPYPLIGNGHLLAVDSSEFIRVFQRLEEE
YDQTTRIYLFSQRFVLTANPKFIEEVVSSTKLIEKGISYEYVKPWLGEGLLTSSGPRWKAHR
KFLTPAFHFNILQQFLPIFCKNAKILSEKLRKLATKESSLNIFPIIALAALDNVTESIMAVSFN
AQRDTESEFVKAVDTMSSIISRRMRNPFLDIVFNLTPYRRAQEKALKILHGQTIKIINARQA
DLKKMNLAELELHSDHGIKNKHTFLDLLLLAEVDGKKISDEWIREEVDTFLFEGHDTTTS
GITYCLYCLAQNQDVQEKVLEEQQSIFGNDFCGNISGGHLQQMKYLELVIKESLRLFPSVP
FIQRKISEDTVINGLRIPKDTSIVIDFFNMQRHPRLYDDPLKFGRERFENTARNPFTWLPFSA
GPRNCIGQKFAMMEMKVTISEIIRKFKVLPTGEEPQLSADLILRSLNGVKLKIVPRS

>CsupCYP4M38

MLFIILISTVCFLVFLHVLFNCHEKARLVRSIPGPDNIFLIGNALEFLATPVEIFGLTRKAANK
WKIIYRFWAFNLAAVNVFTPEHVEKIISTTKFNSKSILYSFMRPWLQDGLLLSDGAKWQQR
RKILTPTFHFNILQRFCVIIEENSRRMVEMLNNSAGEKVDVVPLMSEFTLNSICETAMGTRF
SEEMADKGESYKAAIHSIGKGLVQRVTRIMLFSDLIFNLSAFGREQIKSIQIVQNFTKKVIK
DRKANIEQYGAENLDDMDNNEYNMNNKRKRTAMLDLLIQAQKVNLIDDQGIQEEVDTF
MFEGHDTTACGLMYCLMLLANHKDIQDKIFEELFTIFGNSDISASYDDLSKMRYLECCIKE
SLRLYPPVPFISRQLTESTDLGGYKIPSDVFCHIHIYDLHRREELFPDPLKFDPDRFLPENCIG
RHPYAYIPFSAGPRNCIGQKFAMMEMKSAISAILRRFEIYPVTRPWDLRIAADLVLRSTEPIY
VIFEKRNTLSSK

>CsupCYP4M39

MIVYFLYIVIFLCCVHVVFNYNGKARALKRIPGFRDRFIVGNALELLLSPVDLMQLPRKSA
KNLAKIFRFWSFPIGAVVIYDPEDIEKVLQSMKHNEKALIYRILQPWLKDGLLLSNGLKWQ
QRRKILTPSFHFNILKQFLVTLEENSARLVKDLEGKVEQEIDIVPLISEFTLNSICETSMGTKL
SDETSSAALSYKNAIYELGKKFVDRFTKVHYNIDFIFALSSIGRQQKRYLEIVHDFTKRVIK
DRKEYIETFGIDIDEMNVSDEDIFFKSKRKTAMLDLLISAQNKGLIDDNGVQEEVDTFMFE
GHDTTAAALAFCLMSIANDKGIQERLFSELDGIFGDSSQPATMDDLSRMQYLECCIKESLR
LYPPVHLMGRTLGETVTLSDYVVEKGTYILILIYDMHRREDLFKDALKYNPDRFLPENSIG
RHPFSFIPFSAGPRNCIGQKFAMLEMKSSLSAILRRYRLEPVTGHGDIEITADVVLRTSHPIL
VKFVRREK

>CsupCYP6AB45

MAKSYLQQKSITEMTVYNYWKYPKEKVVGMYMSSWPALVLRDPEIIKEVLTSDFLYFYP
RGLNPHEEVLEPMMKNLFFVDGDLWRLLRQRMTPAFTSGKLRAMFPLIVERAEKLQTRT
ANAAAKGNALDARDLMARYTTDFIGACGFGLDADSLNDEESAFRKLGIKIFEVQLIDAVK
LFLRDLFPVALKRMKVMGKVEGDIHDLVKSILKQRNYQKSGRNDFIDLLIECKNKGLLKG
ESIERKKQDGTPEEASAILDEMLMAAQVFVFFAAGFETSSSATSFTLHQLAYNPEVQKKVQ
DDIDQVLARHNNKLSYDAVKEMKYLDWAFKEGMRMFPSLGFLIRKCAKQYTFQKLDLTI
DPGVRVFVPLQALHNDPLYFDNPEEFRPERFDPEVFDSRQKNIYLPFGIGPRACIGERLGH
MQSLAGLAAVLSRFSVRPAPETVRYPTVSPFSDIVQSIKGGLPLLFEAREISSS

>CsupCYP6AB48

MIFTVVLIVAFILFYLYNTRDFNYWKERGVKHDEPMLLFGTNAKNYLLQKSYTEMLVETY
WKYPKEKVVGIFRSSSPGLVIRDANFVKRILTTDFAYFHMRGLNQHPEVFEPLFRNIFFAEG
DVWRLLRQRMTPAFTSGKLRAMFPLIGSRAERLQAYTAAAAAAGRTLDARDLMARYTTD
FIGACGFGLDTDSLNDESSAFRKLGANIFNFRFRDILTLYLKDMFPEIFKNFKIMGQVEDEII
SLVKAIMKERNYQPAGRNDFIDLLLECKSKGLMIGESMTKVKDDGTPEMASMELDDVTM
AAQVFIFFVAGFETSSSATSFTLHQLAYHPEIQKKVQDEIDRVLLKHNNKLNYDAIKEMTY
LDWTFKEGMRMFPSLGYLLRKCARQYTFPDTNITIDAGIRVFIPVQAMQMDPLYFDNPEE
FRPERFDPEVFDSSLKYVFLPFGTGPRACIGERMGQMQSLAGLAAVLSRFTFRPARESVRQ
PSVMPASGVIQIIRGGVPLLFEERKMTA

>CsupCYP6AB46

MIFIALLLVALVVLYIHGTKNHNYWKDKGVKHDKPVFLFGTNTRNYLLRKSFTELLAEYY
WKYPAEKVVGAYRSNTICLVLRDPDLIKQVLTTDFLYFYGRGLITNEDVVEPMMKNLFFV
DGDLWRLLRQRMTPAFTSGKLRAMFPLIVERAEKLQTRTANAAAEGRSMDARELMARYT
TDFIGACGFGLDADSLNDEESDFRELGVKIFTITFRDAIVIYLKDLFPGLFKSFKQLGAVEH
ELTDLVKAILKQRNYQPSARNDFIDLLIECRNKGILKGESIEKFKQDGSPEVASAVLDEALM
AAQVFVFFAAGFETSSSATSYTLHQLAFNPEVQEKVQEDIDRVLAKHNNKLSYDAIQEMT
YLDWTFKEAMRMFPSLGFLIRKCAKKYTFPKIDLTIDPGVRVFVPLCALHMDPQYFDNPQ
EFRPERFDPAVSDSTIKNIYLPFGVGPRACIGERLGHMQSLAGLAAVLSRFSVRPAPQTVRH
PKVNPASGIVQSIKGGLPLLFEERKSCRP

>CsupCYP6AB47

MFFIALFLIALTALYWYSTKNFNYWKDRGIKHDKPIPLFGTMARNYLFQKSLTEIAVYNYW

KYPKEKVVGMFMAIAPALCLRDPEIIKEVLTTDFLYFYPRGLNSHDEVIEPMMKNLFFADG
DLWRLLRQRMTPAFTTGKLRAMFPLIVERAEKLQTRTANAAAKGKALDARDLMARYTT
DFIGACGFGLDSDSLNDEESAFRKLGIKIFKVEIMQAIVLCLKDFFPEVFKHFKILGNVEGD
VKDLVRAILKQRNYQPSGRNDFIDLLLECREKGTLKGESIERKKEDGTPEEATAVLDEELM
AAQVFVFFAAGFETSSSATSFTLHQLAFNPEVQKKVQDDIDRVLAKHNDKLSYEAIKEMR
YLDWTFKEGMRMFPSLGFLIRKCAKQYTFQKLDLTIDPGVRIFVPLQALHMDPLYFDNPQ
EFRPERFDPDVFDSNLKNIYLPFGIGPRACIGERLGHMQSLAGLAAVLSRFSVRPAPETVRH
PTVSPSSDIVQSIKGGLPLLFEPRNISGS

>CsupCYP6AB49

MITVIIIVLTLIAIYLYNTRNFNYWKKRGIKYQKPVPFFETNTENFLLRKSYTETLTNWYKC
YPNEKVVGFFRASIPGLLIRDPELIKRVLITDFAHFYERGLHPDKDGIEPLLQNSFFAEGDLW
RLLRQRMTPAFTSGKLKAMFPLIVERAKHLQARALAASWEGSAIDARDLMARYTADFIG
ACGFGLNAGSLNEENSAFRKLGQEIFEFNFKMALTTILKDLVPTLTRSLKYMDRVENQVFS
LVKQILEERNYKISNRDDFIDLMIDCKNKGSIEVESLQKVKPDGKPELARLELTDKLIAAQV
FVFFAAGFETSSSATSYTLHQLAHNPEAQKKVQKEIDDVLSKHDNKLSYEAIKNMSYLEW
TFKEGMRLFPSLGYLARKCTKKYTFTDLNFTIDEGVFIIIPVQGINTDPQYFDEPLKFRPERF
SPRNISETNKYVYLPFGDGPRACIGARLGLMQSLAGLAAILSKFTVEPAPETKKHLTVEPKF
DIVQNIEGGLPLRFIER

>CsupCYP6AB50

MITIALKVILAITFYIYNVRNFGCWKKRGIKHDKPVISFGTNTWNILMKMSRSELAEELYF
KYPKEKVVGFYRSSMPELLIRDPEIMKWILTSDFKCFYGHGAMFPLIVERCEKLQSRTLSA
VTCNRALDARELMARYTTDFIGACGFGVDSDSLNDENSAFRKLGVKIFNISLRDSIVLVIR
DVFPYSEKYLKLLKRIEPDILLLVKNILTERNYKPCGRNDFIDLMLEYLNKPMEAESIENVG
ENPIHVKMILNEGLFAAQVFVFFVAGFETSASSTSYTLHLLAFYPEVQKRVQADIDRILAKH
DNKLSYEAVKEMTYLEWTFKEGLRMFPSLGFLMRRSTRKYKFEDINLDIDKGVNIVIPVQ
ALHMDAKYWDDPDEFRPERFHPENFGATQKMVYFPFGEGPRNCIGSRLGLMQSMAGLA
AILSKFTVEPAPETVRYPQISPKSNIIQSITGGLPLMFRERKQV

>CsupCYP6AB51

MITIAVVVILAVTFYIYNVRNFDYWKKRGIKHDKPVFLFGTNARNILMKKSRSELAEELYF
KYPNEKVVGFYRSSMPELLIRDPEIMKKILISDFTSFYARGFHPYKQKVEPLMKNLFFAEG

DLWKMLRLRMTPAFTSGKLKAMFPLIVERAENLQQRALNLAKKNIPIDARDLMARFTTD
FIGSCGFGLDSDSINDDDSEFRRLGRDAFNPPLTTVICGILKMLFPTICKDLQFFGHVEKRSF
DLVNKIQASRNYKPIGRNDFIDQMLEYKKRGPIEIESIEKVQSNGKAERLSFELTNELIVAQ
VMVFFGAGFETSSSATSYTLHELAYNPEIQKKVQNEVDEVLRRHDNKLSYDAVKEMTYL
EWTFKEGMRKFPSLGYLMRECTRKYTFEDLNFSVDEGVKIMISVAGLHNDPKYWHNPEE
FRPERFGPEEFTSIQKDVYQAFGDGPRVCIGARLGLMQSLAGLAALLSKFTVKPAPQSIRK
PPVVPTSDLVQNIDGLPLIFIERQ

>CsupCYP6AB52

MFFVTIVAIFTLILYFYDTRTFKYWKERGVKYVEPVLFFGTYTDVYLEKKSRSQIAEEIYW
KYPNEKVVGAFKSTTPELIIRDPDLIKHVLINDFPSFYCRGLNHTYDVEIFRKHLFAIDGDL
WRMLRQRMTPAFTSGKLKAMFPLIVERCEKLQSRTLSAVTGKRALDARELMARYTTDFIG
ACGFGINSDSLNDENSPFRKLGANMFKVTLRDSIVAVLKDVFPYSEKYLKTFKRVEPDILN
LVKRILTERNYKPCGRNDFIDLMLEYLNKPMEAESIEKVGENPIHVKMILNEKLFAAQVFV
FFGAGFETSASSTSFTLHQLALNPDVQKRVQEHIDQVLTKHNNKLSYEAVKEMTYLEWTF
KEGLRMFPSLGFLMRRSTRKYKFQEINLDIDKRVNIIIPVQALHMDSKYWDDPEEFRPERF
HPDNFGASQKMVYFPFGDGPRNCIGSRLGLMQSMAGLAVIFSKFTVEPASETVRYPKVEP
KSNVVQSIRGGLPLMFHERKQV

>CsupCYP6AB53

MFIILSIFVTLVFVLYVYGTRNFNYWKKKGIKHDKPYPFVGNAFNQLVFKDTITDFFVDIYR
RYPEERFVGVYMSNDPILIVRDPKDVQRVLISDFHNFYPRGVLKHQDEPDPLVKHLFALDG
DLWKLLRQRMTPAFTTGKLRAMFPLIIERAEKLQLLLADSARKNEEIDVKDFLARYATDFI
GACGFGINMDALQDKNSIFRKLGLRIFKANFFDIVRNTLKLLFPKLTKYVATVPPDVTKMT
THLVSTIMAERNYEPSGRNDFIDLLLEVKKKGKMIGESLEKKNPDGSPVIVEQELDDLLIA
AQVFVFFAAGFETSSSTSSFTLHQLAFHPENQEKCQKEIDEVLKRYDDKLSYDAIKEMKYL
KQCFFESMRIFPSVGVLIRKSASKYTFPDSDLTIDENVQVMVINQAMQLDERFFDEPEKFIP
ERFDPENANNIPNHVFLPFGEGPRACIGKIINVVI

>CsupCYP6AW1

MFFSVTLLLLMIITFLTFTLAVFHFTTKGRKYWQLKNVPYKEPCPIFGNFGPIFTMRKSYAS
MLNYFYDKYRNEKYVGIFQARRPTLMVIDMDLVKSVFSTEFSNFSDRVSAVTDTQREPLL
RNLANMSGAEWKAMRHIVSPTFSSAKMKAMFPLIADCAHTMKKILLEEASAEVDIPKLM

CRYTTDVIGSCAFGVDPGSLKNPDSPFLKMSSKMFKPDRSTILKRYCRVFFPRLFKLLNLRS
YSPDVEMFFTSIIKQVLSERRNTEMVRHDFLQLMLNVQNNDSTFVMTDELITSNSFIFMLA
GLETSSTTLSFCLYELAKDKDVQDRVRKEIMQYLTSYGALNYDSVSAMRFTTQAVIETLRL
HPPTPMTTRLCTSPCTLTGTDLKLKVKDPVLIPIICIQNDSKYFSNPNKFDPERFDDDLSPPG
FFAFGDGPRSCPGERFAQLIVVAGLATILSNFSVEPCARTTPTIQYDSRSVMLKNKGGIWLR
FKPL

>CsupCYP6CT1

MAVFVILAVCSFILIYVWFQFKFTYWSRKGVFGPKPIFPYGNIKDVITRKSQFFQPYCDIYF
KYKHLPYVGMYCFHRPVLCVNDLELAKHILVRDFEHFQSRGTYSGGEGDRLAGNLFNIH
GTVWKKLRLKMSPSFTSGKLKIIYPIMVKIANDALTYADMLHRNGESVNFSELYGRYAMEI
IANIGFGVECNGFNYAKSEFYDRGREYFDPRTLYWTIIRGFAFIAPDTFEKLKLKRISSRIEN
FFYSLVKHTVEHRKKYSYKRNDFLQTLIELMEEQDKKGVTNRIEFPFTMDDLAANTMLY
MIAGYETSATTGQFAAYQLARNPHIQNKAREEILGVLHKYGGQFSYEAQNEMVYMNMV
LEETMRMYPSMRAIFRRCTKDYQMPGSDLVVEEGTLVFVPCQAFQMDPEIFPEPEQFDPE
RFTPENKAKLHPCHWMPFGEGPRKCLGVRQGYIQSKLALVKILQEYELQLDQRTLVPMRI
KTTSLVPQAEGDVWIKLRRLDEIKTEA

>CsupCYP6CV4

MILNACFCFTLLLLSLCCLFCYKKFSYWSDKNIKGPTPWPIVGNFANVMFQKKSVNQCIK
DVYFKYKGEKLVGLYRGFQPMLLVNDPEYIKHILVKDFNCFQNRGMSTSKSRLSDNLFSA
EGEEWKILRQKLTPVFTSRKLRDMVPIIQECVESFLLFVDTLANRNVDYEIRDMTSKYTM
KVIGKCAFGLDVDTFTDEKNQFSEMANLIFKQTLSDRLLTVLDMIFPGLKKLFVTRLDIQD
YFVSLVRKVLLEREGKPLIRKDFIDFMLELRDCGKINRKLDNEKVCEIEIDDYMVAAQAFV
FYTAGYETSSATMSFMIHELALNPEIQDRVYEEISQVLAKYNGEINYESIKYMKYLEMVFD
ETLRKYTVAGILFRKASSRYTFAGTNLTIEKGTLVLISATALSTDSAYFPEPDKFNPENLSSE
NLSKLPKCVYLPFGEGPRNCIGMRFGKILSLLAMAAFLKKFKVEPANKTKRELTFNPKGMI
LISNEGIWTKISRRQT

>CsupCYP6CV5

MALIACFTLSVILVTVFCLYCKKKYSYWREKGVKGPVPWPVVGNFGNVILQKESLDQCIK
DIYSKYKGEKLVGLYSGFRPVVLIRDPELIKHVLIRDFDVFQERGNSSRGSRLSDNLFSANG
EKWKIMRQKLTPVFTTRKLKEMVPNIQKCIANFLQYVNNLVEKNIDHEIRALTAKYTLEVI

GSCAFGLDLKLYTDEENKFSSMAKQIFTPTFRSRLIVLLDMIIPGIRKKFSTRPELQEFFVDL

VRKVQKERKGRPLSHRDFMDLMIELKEEGKISRKLEDGKVEIEIDDYMVAAQALVFYSAG

FETSSAAMSFLIHELALHTDVQDRLYKEICEVLEKYDGEINYDSVKDMKYLEMVLDETLR

KHSLATVLFRKSSLAYTFPGTDVTIPKDTSVMISASGLTKDPKYFNNPNEFNPENFMPEKL

NNLPQCVYLPFGEGPRNCIGMRFAKVQSLLGTAAFFKQFKVEPSEKTKQELVMDPKAIVV

TCKEGIWVKITKR

>CsupCYP9A68

MLFLIWCLVLLASLALFFKGKYSRFSSYGVKYLRPVPLLGNMSSILFRIEHFSEQIYRLYNE

FPNERFIGRYEFLNAVVMVKDFELIKRITIKDFENFLDHRNILDEKTDPLFGRNLFSLKGQE

WKDMRSTLSPAFTSSKMKLMVPFMVEVGDQMIETLKAKIDASKSQYLDVECKDLTTRYA

NDVIASCAFGLKINSLVEENNEFYRRGKEASNFTFRQVLVFFGSQNFPKLIQFFRIGMFKDV

TTKFFKQIVMSTMNEREIQKIIRPDMIHLLMEAKKGKLLYEEQTTNGSDAGFATVEESNVG

KRNIKKVWSDIDLVAQAMLFFIAGFDAIAAIMCFALHEIALHPEIQDKLVEEIKENELKNGD

KFDYNSVQNMTYLDMVVSEVLRLWPPGIVLERQCNNDYNIGKANDKCQKDYIIRKGEGV

LIPVWGIHRNPEFYPDPMKFDPERFSEENKHLIKPFTYLPFGVGPRNCIGSRFALCEVKVML

YQLLQHIELSPSVKTTIPVKLSTDTFNLKMKNGHWVKMKLRE

>CsupCYP9A69

MMLILIWLTVLVVAFALYLKENYSKFKRKGVKGPKPIPLLGTGIGVFFGKEHMTDYMVRM

YQTYPNERFFGQFELTRPVVVVRDIDLIKQVTTKDFEYFLDHRSMVEADIDPLFGRNLITL

KGQEWKDMRSTLSPAFTSSKIKLMVPFMMEVGNQMTESLKKKIKESNVNYIDVDSKDLT

TRYANDVIASCAFGLKVDSMMDEDNEFYKMGKFVTNFTTLMLVKVLFFIYTPAIMRILKI

KFLPENTSNFFKGLILKTMKDREKNNIIRPDMIHLLMQSKKGKLAHEENACTDKDTGFAT

VEECNVGMKMDQKDWSDDDLVAQALLFFLGGFDTVSTAMTFSLHELALSPEVQDKLLA

EIRENGQKNNGKFDFNSIQKMVYMDMVVSEVLRLWPPAPVLDRICTKDYNIGKANDEAT

QDYIVRKGESVSLAVWAIHRDANFFPNPLKFDPERFSEENKHNIRPFSYMPFGLGPRNCIAS

RFALCELKVMLYQLLQHFEISPCEKTCIPSQLEAGKFNLSLKGGHWIRIKIRE

>CsupCYP18A1

MFMFLQNSKILWGIGQVVYFCVSRASTPLLLTFAVALLVARLLLVLREIKKLPPGPWGPPVV

GYLPFLGIRHKTFLQLARHYGSLFSARLGNQLTIVLSDHKLIREAFRREEFTGRPNTPLMHT

LDGLGIINSDGRLWKSQRRFLHEKLREFGMTYMGNGKRVMETRIKHEVHDLIENIRRTNG

LPVDLNPMLALAVSNVICGITMSVRFSHGDARFERLNFLIEEGMRLFGEVHYGEYIPLYNY

LPGKAQAKEKVVKNREEMFAFYQTLIDEHRQTLDINNARDLIDTYLIEMDKAKTEGRSKD

LFEGRDHELQLKQILGDLFSAGMETIKSSILWMIVFMIRNPDVKQKVQEELDAVVGPNRLP

NMEDMARLPYTETTILETRMSSIVPLATTHSPTKDVHLNGYKIPAGAQVVPLINCVHMDP

NLWDEPKKFNPSRFIDENGKIRRPEYFMPFGVGRRMCLGDILARMEMFMFFSCLMHQYD

VVLHEGDPLPSLEGIVGATIAPKAFRVKFVPRSAPEPVAPVSLPDHLTLRNVGAH

>CsupCYP301A1

MAPTAELNEEVFTNAKPYSQVPGPKPIPILGNTWRMVPVIGQFDISEFAKVTKMFMEKYG

RIVRLGGLIGRPDLLFVYDADEIERMYRREGPTPFRPAMPCLVKYKSEVRKDFFGELPGVV

GVHGEQWRRFGSKVRRPILQPQTVKKYVTPIELVTLDFIKYMEDARDEHGDLPAEFDNDI

HRIGRVALDVRLGCLSSRLSSASDPQRIIDAAKYALRNVAVLELKAPYWRYFPTPLWTKYV

NNMNFFVELCSKYINEALERLKTKKVTSENDLSLLERVLKSEGDPKIATIMALDLILVGIDT

ISMAVCSILYQASLRLKQQDKMAEEIKRVFPDPDKPITYADLDKLHYTKAFVREVFRMYST

VIGNGRTLQEDDVICGYHIPKGVQVVFPTIVTGNMEQFVSDPEEFKPERWLESDGRLHSFA

SLPYGFGARICLGRRFADLEIQVLLAKVSYAI

>CsupCYP307A2

MSAILLAVVIVLIAYKFLLPRKNTVTLKRVNRTGEVINTLRTAPGPTPLPIIGNLHLLGKHES

PFQAFTELSKQYGDIFSLKLGNTPCLVVNNLELIREVLNQNGKFFGGRPDFIRFHKLFAGD

RNNSLALCDWSNLQTRRRNMARRHCGPKQHTDNFTRIGSVATFESIDLIQTLRSIANTTES

SINLKPMLMSTAMNMFCHYMCNVRFDPENDVEFKKIVDHFDEIFWEINQGYAVDFLPWL

EPLYKKHMDKLSGWSQDIRTFILSRIVEQREMSLDVEGPEKDFLDGLLRVLHDDPSVDRN

TIIFMLEDFLGGHSSVGNLVMLCLAAVARDPEVGKKIKAEIDGHTKGKRAITLSDRSSLPFT

EATILECLRYASSPIVPHVATENASVAGFGVEKGTVVFINNYELNTSEQYWREPEKFDPSRF

LERTKIRVRRNSHCDSGMESDSERTGPIDKATETEKEVVSVRKNIPHFLPFSIGKRTCIGQTL

VTTMSFVMFASIMQEFDVAAENLEDLRQKPACVALPKDTFNLYLVPRKH

>CsupCYP321F1

MFLLLSLVVTIFIFTFAFIIGRYNERHWKKRGVIFDKRNNLIIGPYYEFLTGNRPLFEIFGDM

YRKYRDEPAVGFSTLFTPSLFVIDPKNVQYVMQGPMFIDRGIDYHKEDYLADNILFMSGV

RWKLMRQKMTPLFTPIKLKNMYYIIEKRTQDFIQYLHKNPEKQKGNTFNMLSTFCCNAIG

DAVFGVSYASTFISPFLDITRKAFSSNWWTDIRFTISSVSPTIFKALKITFFKQFEQFFIGAMK

QVFRQREKEGVKRHDFADMCLSLQKSGVMKHEETGFQMQPTDELLAAQGFFFFTAGVEP
TAAAVFGAMIEIGRHPEIQKRLQEEIDGAFDKNNGQLTYDAVMGLEYLDKVLTESLRTFPPI
GFLTRRCTEETFLPVGNIKIDKGTKIYIPSYAYHHDPEFFEQPHVFDPERFSDDTKQRLGVIY
QPFGFGGRICIGMRYAKLQVKSVLAHIIHHFDLKTVIGSGGIKFAKDQTQVRIKNVDVVYF
PRKLIS

>CsupCYP321F2

MFLFLLLVATVIILTFVFFIGWYNERYWRKRGVIFDKGNNHIIGPYWQFLTGDRALFEIFGE
MYKKYHDEPAVGFSTLFTPSLFILDPKNVQYVMQGSMFIDRGIDYHKEDYLADNILFMSG
VRWKLMRQKMTPLFTSIKLKNMYYIIDKSTKDFVQYLHKNPEKQRGNTFDTLSTFCCSAI
GDAVFGVSYASTFVTPFLDITRKAFSASHWNNLIFTMSNFTPALFKVLQLKFFKQFEIFFIA
AMKQVFRQREKEGVKRHDFPDICLSLQRNGVMKHEETGFQIQPTDELLAAQGFFFFIAGV
EPTATSIFGAMIEIGRHPEIQKRLQEEIDRVFEKYNGQLTYEAIIDNEYLDKVIKESLRMYPP
LGFVTRRCTEPTILPVGNIKVDKGTKLYIPIYAYHHDPKYFVEPYVFNPERFDDSKKNFGVI
YQPFGFGNRICIGMRYARLQVKSGLAHILRNFDLKTVVGSGGIKFGKDRIQLRVHNVDVIY
TPKKVKST

>CsupCYP321F3

MFLFILLLFTIFVLTAAYLKGRYNETYWRKRGVKFDNRNRLMGPFWDFITGDRAFFEILVD
MYKKYPDEPAVGFGSLWTPALYVKDVTNVLYVVQGSMFIDRGVDYIEDDYLADNILFMS
GARWKLMRQKMTPLFTSAKLKNMYYIMDKSAQDFIEYLHNHPVKQKPKETYETLTTFCC
AAVGAAVFGVSSKSTFDSPFLDMAKKAARANLLTNIKFALASLSPKLFKALRLKFFKEFEP
FFIGAMKQVFRQREQENVKKHDFADICLSIQKNGVMKNEDENIEMQPTDELLAAQGFFFF
IAGVEPAASSVFGAMIEIGRHSEIQKRVQEEVDNVFDKYDGKLSFDAISEMEYLDNVINEA
LRVYPPIGFSTRRCTEDTVLPVGNIKIEKGTAIFTPIYEYHHDAKYFENPQEFNPDRFADKK
NLGVIYQPFGFGNRTCIGMRYAKLQVKCGLAHIIHNFELKTVVGKGGIKFHKEQVQLRIEN
VDVEYIPRKEKRM

>CsupCYP338A1

MLNLFIIIVLIVALTFDYVTKFFSYWYVRLIPNNTPIPFFGTDYYRVLGIRSNTEEVNALYAQ
YPKEKFVGAIKSRIPDLVVKDPDAVKTILSTDFADFCRRGHSLDKSRDVCMRNNLFYAEGE
TWTLLHNSVESIFNDMKYDFEESLHDRLSGTNGDTNVQQLLSKVLDCFFKVILLDGRSDE
TLITYVRTAAQRRTFMEKLKTYLKHVFPSLFVLFGMTTIPGEPSSKTKRELEKSKILNKIRN

SNIYPLRSNDKHKGSSSDIEFAFGILATFITEGYIPCLNLLTALFYDLATNPETQGKARSNDE
YLNAAIKETLRLHPPYSVISRQCVKMYHYPDSKLVMDKNVTVNVPVESIHRDEKYYKNA
ETYNPERFLGGENSSHSFAYLPFGAGPRKCLGKLFLPLFRDVVCL
>CsupCYP341A15
MILILACFALALWIVRFRYRRQHMYKLASKIPSPTKEMPVIGLAHKLMGSTEDIMDEMKA
YSYAAMENGGLVKGWLSHILYFMLLDPVDNEKVLKTCMEKDNLHRFIRAVLGNGGIFAS
VKIWRRRRKILVPAFSPKIVESFVKIFTQQSETLVKKLSNQVEKGKFSVWPFLSTYTLDSVS
QTAMGVKINAQNNTHSPFLVSLNRILHLVCERIFHLWLQPDWMYMLFPQYTEHKACLKQ
LHDFIDEVIIKKREEIQKESKEKTEADAEYDLDNYKTKSFLDHLIHLSGKEGGYTNLELREE
VLTLTIAGTDTSAVAMGNTLKMLATYPEVQTRVYEEIYDIFGDSGRQLVKEDLHQLKYLER
VVKETLRLFPPVPFIIRRVDENVTLPSGRVLPAGSGVVVSIWGAHRDPKHWGPGAEKFDPD
RFLPERFLPQHVCSYMPFSQGPRNCLGYQYALMSIKIAVAAIVRNYKVAPDLQNSDKPYIR
VKLDVMMKDVDGYQIALEKRKPSPNTNVTISS
>CsupCYP367A9
MYITLVFLIALPVFLYWLYWRQANKRLLELASKIPGPPALPFIGGARMFIKNPKDQIKIIASL
FQNYGEYVRFWLGPDLNIAVKNPSDIRLLLTSTNVNKKGPLYLFLEPFIGPGILCGGSTWRL
HRKIATPSYNKKAVGLFMQVFNEESEELVKILCKKGPETYDVYHDIVQSTTSAVCQTLMG
LPKEELKEIKYLQAIIEETPKIYDLIFRKMTLWWLQIPLVLWLTGNLKLQNYYMKLVDDFS
EEVVMRRRKALEKNVGPEESMGVVDRYILSKEMSHQQIKWDSFTLFTTSQEAAAKIASG
VIMILGLLPDWQKKVYDEVITVVGPDAAVNPEHLKQLHYLDMVYKETLRYFSIAGLIQRT
VVSEIAIKDGEIILPVGTSLLIPIHELHRDLKNWEDPLRFDPGRFLPENVQKRDPNAFVPFSL
GPMDCLGRVYATSLIKTMAVYLVRKLHIESEGPDNDIDLDIAISVRPRQGYRVTTRPRHAN
GYSKTNGVNNKHQLLNL
>PxylCYP9F2
MITEILVFIFTTIVFYALYTYKRGHYYFQSRGLKYIQGVPLFGNTLRSTFGGHHMIYDIDQV
YKAFPNEKYVGYMEGTKPHVLVRDPALIRQLTVKDFDHFLDHKSFFTDQIFMDSLFMMK
GDRWRHMRATLSPAFTGSKMKQMVPFMNEISLNIVDYLKEKEGETINVDDIIRRYSNDVI
ASTAFGIQVNSLKDPDNEFYRTGQAVMTFTLYQKMKLVFMTMFPGLMKHADIFPQSATEF
FKRIVSETMQYREKNNIERPDMIQLLMEAKKGTLKAQAEDKLDKIGFATTENIEVKPQLEK
SDWTQDELAAQAFLFFAAGFESAASAIALAIHELAINPAVQSRLYDEVKQTHDKHGRVNY

EVIQGMKYLDCVFSETLRKWSPAIVMDRVCVRPYTLPPASEGAKPVKVNVGDVLYNVVN
SLQLDPQYYPDPLKFDPDRFSDERKHEIQPTTYVPFGNGPRNCIASRFAILEAKVLMFHLIL
NFEVLKTEKTLDPIVLRAGDFSIKVKGGSYVQCRARK
>PxylCYP6BG1
MLYAAALIVVALAALYWWSTSKFDYWSRRGVKFPRPAPLLGNYGAYLLQRRNLGEVTRA
ICRQFPDEPLVGAFYGPHPVAVVQDPALLKLITTKDFYYFNGREVSEHTHKETLTRNLFFTY
GDKWKVLRQNLTPLFSSAKMKNMFPLIECCTHDLEKLLDEQSKTNKNGVDIRSVISRFTM
DCIGACAFGINTGVMQKDSEVNPFRQIGDKIFEVSIIRGFLTAFRSIWPSLFYALRLSMFPPQI
PAFFHSVLMTVFKERSLTASSRQDFVDMILALKKSNYITGDSVNNMKGGNDKLQIKVDDE
FLVAQCVLFFAAGFETSSTTSSFTLFELAKQPELQQRVIDEVDSYLSTRSEVGYDCTTELPFL
EQCIEETLRLYPVLPVVTREVVEDYTLPSGLTLEKGLRVHIPMTHLHRNPKYFPDPDEYNP
DRFSPENKKNIVPFTYFPFGEGPRICIGMRFAKMQMMAGLVTLFKSYRVELAPDMPTEVT
YSSTAMVTQFTTGIKLILVPRVKTAN
>PxylCYP6BF1v1
MPYLDVAVALLAAFIAFTLWTNRRWNYWKKQNVKYLTPIPFLGNVADVIFQRDTFGAVTQ
RICQQFPDEAVVGMFYCSNPAALVQCPDMLKTVMVKDYAYCSSKEVSVHSHKEPMTKN
MFFTFGDKWKLIRQNLTPVYTSAKMKNMFPLVQDCCRIFQKVLDDEIGKGRVVEVKSLIA
RYTMDCITSCAFGVDSGTMSKGEEGNPFTETGHLLFDERPIAGVKNVLRYGYPSFFYSVG
LELYSSKIYRFFRSVILDVINSRNGAKSSRNDMVDLISDWKKNKYITGDSIDNGIDGGNKK
VRIEVDDELLVSQCVLFFQAGFQPSALTSAYLLYELAKNQDIQERVLAEVDEYWSTRDEV
QTDCVTALPFLAQCMEESLRMYPPVSVLMREIYKDYTLPNGVHLKKGMMIHIPVYHLHH
NPKYFPEPEVFRPERFSEEGRKSIVPYTYLPFGDGPRMCIGYRFARLEIFSSLAVLLKKYRVE
LAPHMPRKLQFLTTSRVLTSIHGIHLRLVDRVN
>PxylCYP6BF1v4
MPYLDVAVALLAAFIAFTLWTNRRWNYWKKQNVKYLTPIPFLGNVADVIFQRDTFGAVTQ
RICQQFPDEAVVGMFYCSNPAALVQCPDMLKTVMVKDYAYCSSKEVSVHSHKEPMTKN
MFFTFGDKWKLIRQNLTPVYTSAKMKNMFPLVQDCCRIFQKVLDDEIGKGRGVEVKSLIA
RYTMDCITSCAFGVDSGTMSKGEEGNPFTETGHLLFDERPIAGVKNVLRYGYPSFFYSVG
LELYSSKIYRFFRSVILDVINSRNGAKSSRNDMVDLISDWKKNKYITGDSIDNGIDGGNKK
VRIEVDDELLVSQCVLFFQAGFQPSALTSAYLLYELAKNQDIQERVLAEVDEYWSTRDEV

QTDCVTALPFLAQCMEESLRMYPPVSVLMREIYKDYALPNGVHLKKGMMIHIPVYHLHH

NPKYFPEPEVFRPERFSEEGRKNIVPYTYLPFGEGPRMCIGYRFARLEIFSSLAVLLKKYRV

ELAPHMPRKLQFLTTSRVLTSVHGIHLRLVDRVN

>PxylCYP6BF1v2

MPYLDVAVALLAAFIAFTLWTNRRWNYWKKQNVKYLTPIPFLGNVADVIFQRDTFGAVTQ

RICQQFPDEAVVGMFYCSNPAALVQCPDMLKTVMVKDYAYCSSKEVSVHSHKEPMTKN

MFFTFGDKWKLIRQNLTPVYTSAKMKNMFPLVQDCCRIFQKVLDDEIGKGRVVEVKSLIA

RYTMDCITSCAFGVDSGTMSKGEEGNPFTETGHLLFDERPIAGVKNVLRYGYPSFFYSVG

LELYSSKIYRFFRSVILDVINSRNGAKSSRNDMVDLISDWKKNKYITGDSIDNGIDGGNKK

VRIEVDDELLVSQCVLFFQAGFQPSALTSAYLLYELAKNQDIQERVLAEVDEYWSTRDEV

QTDCVTALPFLAQCMEESLRMYPPVSVLMREIYKDYTLPNGVHLKKGMMIHIPVYHLHH

NPKYFPEPEVFRPERFSEEGRKSIVPYTYLPFGDGPRMCIGYRFARLEIFSSLAVLLKKYRVE

LAPHMPRKLQFLTTSWVLTSIHGIHLRLVDRVN

>PxylCYP307a1

MSALILIAFVALVVYKLFSRKTVAWRKEAKYGGAQVAVLERAPGPVPFPVVGSLHLLGKS

ESPFQAFTELAKTYGDVFSITLGSSPCLVVNSLDRIKEVLNQNGKFFGGRPDFMRFHQLFA

GDRNNSLALCDWSNLQLRRRNLARSHCGPKQHTANYAQIGNVATFEAVELINTLKQVTSS

TQSAIKLKPFLMASAMNMFTHYMCNVRFDSENDKDFRRIVDHFDEIFWEINQGYAVDFLP

WLSPFYKKHMDKLSNWSSDIRSFILSRIVEQREMNLETDGPEKDFLDGLLRVLHNDSTVD

RNTIIFMLEDFLGGHSSVGNLVMLCLAAVCRDPEIGKKIRAEIDGVTKGKRPVSLLDRKNL

PYTEATILECLRYASSPIVPHVATENAAVAGYGVEKGTIVFINNYGLNKSEKYWNEPEKFDP

SRFLEKSKFRMRRNSLCDSGMESDGERTGPLNKVTELEKEVLSVKKNIPHFFPFSIGKRTCI

GQTLVTTMSFIMFANIVQEFEVAAENLDDLKQKPACAALPKETFGLYLVPRKR

>PxylCYP9E2

MQFLTSNPLVIFILQEWKLLSLLTIIFMLYFYYTKTFTFFEEKGVKFMKPYILLGNLGPRLML

KVSMHGFQLHIYNHFKGSPYGGIFEGRRPVFYVFDPDLIKAVTIKNFDHFVDRNVLNSRQP

GYLSRSLINLKGRDWKDVRSTLTPSFSSTRLRNMLPLIEQCTTQMVQFLKAFDKKEVEMK

DVMGHFTLEVIGACAFGIKCDALTDENAHFVQVASKFNDMAVSKRLLIFFVLIFMPRMFRF

LNLSFLNPESTKELLKILTAAKAERRSTKSKRNDFLQLLIDSAEPEKTESVKPGEHKKHLDE

DTIDAQALLFLIAGYETSSTVLSFAIHVMATHPELQRRLREHVSEVTDGKEMSYEVLAELH

YLEAFLLETLRVYPPVSRIDRVVTKPYTVPGTSINLDPGTVVAIPVYGIHMDPDIYPEPTKIR
PERFLGDEKNDRPSHLYLAFGAGPRNCIGLRFAMFSVKLAMVTLLKNYKFSPCSKTKDPIP
FEKKGILLKAHGGVWVTLESLKE
>PxylCYP4V2
MWLILLLSLLVLIGCVALLKIVAWRRLVKRSAAIPTLSGWVPVMGHVQNLLQSQVKIAEFV
DGIQKETRKRGGITTMWIGPLPTYLITDPDDALTLSNACLKKPYIYNFLDAWLGNVCLLYS
LPHKWRAQRKQLNPFFNPRHLNGFLEVFNEQAAMLVNMFEPHVGGEAFDFKHYLVDITL
DTIKTNMFGRQEDTVGQNKCAYKEAIEDVLNIYMKRFSNIFLHSDLLYQFTKSKGVEDKA
VHLLQKTASDALIKVKSAATSEDQPSDKNFKPFVSLMLELLEDGSLSEAEIQQHLDGFLFA
GHDTSATQLIYIFMAIGHYPEVQERIYKELKEVLGDKPRAVTKEDLPKLVYLTAAIKESMRL
YPIAPVVMRDVDRDVKLKNVTLPSGTFAFILISGINRSGVWGADADQFDPGRWLEPARLP
ASPAAFATFSVGKRDCIGKTYAMMSMKTTIAHIIQNYRLKADINQLKCQLDVMLKPQSGC
ELSVELRRTMSSS
>PxylCYP6B4
MPIKTRQQKLNYYYYICNTADFIESLRILSSNIILVKMLDVYFTIMVIIVVIYYLSQRKFNYW
KVRNVKYLSPMPFVGNYSAYIFQKKTLGETVQSICQKWPDEPLIGAFYGTEPVLIVQDPEY
IKLVTTTDFYYFNGREVSDYIHKETLSRNIFFANGNWWNLFRKNLSPFFTSINIKRMFPLIA
NHCKSLDKLLETETQVSKDLDVSSLMMRYTLDCICSSTLGIDPTALSTTPSNHPFVHVGRM
IFSTSTYRGIQTVFRAVWPNIFYGLGFKMFPNDITDFFLSVVKKQLQEREDERQNRHDLIDF
VAKLQKEEYITGDSIHNLLKNSAPEKIRMKIDDVLLVTQVVSIFAAGFETSATTMSFVLFEL
AKYPEIQQRVLDEIDQYNATKQGGLNYTCITELPYLYQCIQETLRLYPSLQVVTREVFKSY
RFPNGVQVDRGVRVHIPVLHMHRNPKHFKDPHVFNPDRFSLEQKHTVSDYVFMPFGKGP
RLCIGSRFGKMQMMAGLVSIFTKYRLELPTGASTEVQFEPRSMPTQPKKGLHLKFISRNST
FN
>PxylCYP306A1
MDLFFVWLMTFVLGFWICKKIKEWQSLPPGPWGLPILGYLPFLNPKHPHLTLTELSKKYG
SIYGIWMGNIYTVVLSDHKLIREAFAKESFAGRAPLYLTHGIMQGNGIICAEGSLWKDQRK
LITTWLKGFGMSKHSASRDKLERRIGDGVQNLLQDIKRNCDNSIDLSQMMSNSLGNVVN
EIVFGYKFPPDDKTWTWLRQIQEEGCHEMGVAGVVNFLPFIRYFSSDMRKTIEVLKRGQA
QTHRFYASMIAKRRELLGLKKPVGAEYPPHDNLFDEHPEGHIKCVKYSKHAQTTDEHFFD

PSILIPTEGDCILDSYLHEQKRRFEEGDDLARFVSDEQMHYLLADMFGAGLDTTAVTLAWF

LLYMALYPDEQELVREEILSVCPSEGAVDGAMLPRLMAAVCEAQRIRSIVPVGIPHGCLQE

TYVGKYRVPAGAMVVPLQWAVHMDERVWEDPEEFRPARFLDQDGSLLKPQEFIPFQTGK

RMCPGDELSRMLACGLVARLLRQWRLRLASPPPSPQEMRGVVGVTLAPPKVLYRCEPVA

>PxylCYP6B6

MLLLVALAAVALAALYWWSTSKFDYWSRRGVKFPRPAPLLGNYGAYLLQRRNLGEVTRA

ICRQFPDEPLVGAFYGPHPVAVVQDPALLKLITTKDFYYFNGREVSEHTHKEITTKNMFFN

SGDKWRVLRQNLTPLFSSAKMKNMFHLIEKCARDFEVLLEEEAKKSEIVEARTLMSRFTM

DGIGACGFGINSGVMQNDAKNNPFAEVGEMIFEESKWRTFLTAFRAIFPSIFYGLRFSLFPD

AINSFFHSVLMSVFKERKWEPTSRQDFVDMVIGLKKNKYITGDSMRNMKGGSDKLQIEV

DDDFLVAQCVLFFAAGFETSSTASSFTLYELARHPEHQQRVIDEVDAYLATRGEVRYDCTT

ELPFLDQCVEEGLRLHTTIVTREVAEDYTLPNGVKLDKGLRVHIPLFHIQRNPKYFEDPDD

FKPERFSPEERKKIVPYTHFPFGEGPRACIGMRFARMQMMAGLVTVFKSYRVELAPSMPR

EPQYCAGTMVVRFKGGIQLRLIPRENGQKYKPT

>PxylCYP6B6X1

MAALSIVFAALILVITILYFLSKWRYSYWKKRNVPYLEPTPLLGNYGQYILLRETVGEVVS

KILQQFPDEPVVGAFYGTEPALIVQDLQLLKLITTKDYYYFSSREISNHTHKEVMTQSLFFT

YGDNWRVLRQNLTPMFSSAKMKNMFYLIVNCSKEFESLLDKEIKVNDVVDARNLMARF

TMDCIGTCVFGIETRAMINEKDNPFWDVGASILGNTYSRGLRWILRAIWPAIFYGLGFKTF

PDSAVNFFNKFVGAVFKERMKTDAKRQDFIDLLSEFKRNSTIVGDKMSNMKTSSSEKVSIE

VTDEILISQCMAFFAAGYETSANTGSLTLYELAKHPEAQQRVLDEVDRYLRARENKLGYD

CLTELPYLEQCISETMRLYPVLTVLTRELVDDYSLPGGLRLPRGLRVHIPVLHLQLHPRYFP

EPRRFRPERFSPEERHNLLPYTYMPFGEGPRICIGMRFARMQMMAGLITILKKYRVELAAG

MSSSLTLDPRAIVTMPRERLRLRLVPRAGWDTRLYQ

>PxylCYP6B5

MLSPSFCCFIIILLLIYYWFERKSNYWKRRHVKYLTPIPFVGNYSAYIFQKKTIGETVRNICQ

KMPEEPLIGAFYGTEPVLIVQDPEYIKLVTTTDFYYFNGREVSDHIHKEILTRNLFFTYGDN

WNLFRKNLAPFFSLGNMKNMFPLIKEHSKSLEQLLESETRTGDLLDARHLMMRYTLDCIC

SCSLGMDPKALSSTDSDHPFIKVGHMIFLNTISRGIQTVFRAIWPSIFYALGFKIFPLHITNFF

MAIVSKQIHEREGKQSQRHDLIDFVAALKKENYITGESIKKYKSSTNNEKIHMKVDDEIIVP

QLISIFAAGFETSATTMTMVLFELAKHPDIQQRVLNEVDKYNNDNNGNIDFDCTKDLPYL
DQCIQETLRLYPVLLAVTREVFEGYTFPNGVRVDRGLRVHIPVFHIHRNPKYFKDPDVFDP
DRFSPEKKQMILPFTYMPFGKGPRFCIGSKFARMQMMAGLVAIFKKYRIELPGGVSGLVEF
EPRSMSTQTKTGVHLKLIFRHNKME

>PxylCYP321E1

MILAIILLLVIIIITFSFLLGSYNESYWAKRNVKYHGGKNAIATFSEFLFTSRGIFDIFGNIYKL
YPEEPAVATPSLLQPALFVKHPENIQHVLTDNFKNFYHRGVEIAKKDKLAQNVPFLNGSRW
KLMRQKMTPLFTSAKLKNMHYIIDRCAQDYIGYLKEHVNDKNANAFETLSVYSCSSLLA
PIFGIHSGQSTVTSPLLNMARNATKPTLKANLKFILNSLSPKVFQMLGLSFFGEYEEQFIGAI
SQVIRQRKEENVKKHDFADIAVSLQNAGTMKDESSGCEIEPTDEVLAAQAFFFLIAGVDPV
TMGIYGTLFELAKRPDILKQVQEEIDGAFENNTEMGYDVIGTMEYLTKVLEESLRLHPPIS
LLSRECMGESILPVGNIRVSKGTRIDIPILAIHHDPKYYPDPDVFDPERFSAENKNSRPNMTF
MPFGEGGRLCIGQRFAYLQMKTGLAHILRNFTVKVDDVTKKMTYLKSSLIIRPANDINFTF
VPRNKE

>PxylCYP9G2

MIAEILIFILTTLVAFAFYSYYKNQNVFKSKDMKFLPGFPMFGNIIKSSFGKNHMFYDLDRV
YRAFPGESYVGYVEGFVPLYLIRDPSIIRLITVKDFDHFVDHRRFATDDLFNESLFMMTGDR
WRDMRSTLSPAFTGSKMRQMVPFMNETSQNIVQYLRETEGQDIDASRLIRCYTNDVIASTI
FGLQVNSLKDPENDFYKAGQSLVVGNSLTRRPSFFIVMTIPALSKFFPFFPKETTDFFRGIVL
KTMQHRENNNIERPDMIRMLMEAAKGTLKMQTHDKLDDIGFATTDEADIKPKGEMRQW
TPDTLAAQAFLFFFGGFESSASVIVMAVHELAVNSEAQGKLYEEVKEYHEKHGKMTYEG
VQKMTYLDCVANEALRKWSPAVITNRVCVKPYVLPPPREGGKPVQLEVGDGIYNSVSSV
HWDEQYYPEPEKFKPERFNDENKHKIQPFTFMPFGTGPRNCIASRFAILELKVLLYHIVLNF
EIQKCGKTSDPVQLAPGDFNIRAVGGSWVKFRSRN

>PxylCYP9G4

MIAEILIFILTTLVAFAFYSYYKNQNVFKSKDMKFLPGFPMFGNIIKSSFGKNHMFYDLDRV
YRAFPGESYVGYVEGFVPLYLIRDPSIIRLITVKDFDHFVDHRRFATDDLFNESLFMMTGEK
WRHMRSTLSPAFTGSKMRKMVPFMCQTSQNIVEHLKETEGQNINVGELIRRYTNDVIAST
AFGIEVNSVKDQKNRFYEYGQKIINFSSVQKAKIFLSVIFPKSGFQIFPKDSLDFFKHIVTST
MEYREKNNVERPDMIQLLMEASKGTLDGPTEDKSDTVGFATTELTAVKPKGEVQKWSTD

QLAAQAFIFFLAGFDSTASVLVMAFHELALNPEVQETLYQEVREHHEKHGTVTYDGVPTL

KYLDGVLNETLRKWPPTLILDRVCQKPYTLPPPREGGKPYTVKEGEVIFNFVNSLHMDEK

YYPEPEKFKPERFSDENKQNIKLSAFIPFGSGPRSCIASRFAILELKVLLYYVSLNFILQKCE

KTRDPIELTPGEFQVRCIGGSWVKFQARK

>PxylCYP6CV2

MALLTITAFVIFASLLYFWVKHKYAHWEKKGIKSPPVVPFIGHFGRIITFKESIDGFTKRIYE

DFPNEPAVGTYRCLTPAVVLRDPELIKQVMIKDFPVFEDRGIRALDDPINDNLFVAEGETWR

ILRQKLTPIFTSGKLRNMMPLIDKCVGEYLEYVDGLVARGVEHEVRDLSSKYTLEVIGSCA

FGVDMDVFNEKENIFRDVGNRIFSPSLLSRFAQFADFLIPGTLNFLHKTKLYNINERTQTFFL

NLVNKIVGEREGRPKSRKDFMDLMIELKEQKFVEKREHDKVAKLEMSPELMAAQAVVFY

AAGFETSSATMSFLIHELAFHQDIQQRLHDEICAVTEKHGGKLTYDALSEMTYLEMCFDET

LRKYPIVGTLIRKALVDYQFPGTKLKIDKGTVVFLPVGGLQSDSQYFENPDVFDPERFSPE

NKGKIPQCVYMPFGEGPRHCIGMYS

>PxylCYP18A1

MFSNSKLVWGLSQVAHICVTRTSVGVAAAAALLVLRLLALLREVRKLPPGPWGPPVVGY

LPFVGVRHKTFLELARSYGTLFSARLGNQLTVVLSDYRLIREAFRREEFTGRPSTPLMHTL

DGLGIINSEGRLWKSQRRFLHEKLREFGMTYMGNGKKIMEGRIKSEVHELIANLHAAEGA

AIDANPLLALGVSNVICGITMSVRFSQGDARFERMNQLIEEGMRLFGQIHYGEYVPLYNY

LPGKAQVQVKVLKNREEMFEFYQQLIDEHRATLDVDNARDLIDVYLIEIEKAKTEGRAGE

LFEGRDHELQIKQILGDLFSAGMETIKSSLLWMIVFMLRNPDVKRRVQEELDSVVGRERLP

TIEDMPNLPYTETTILETLRMSSIVPLATTHSPTKDVNLNGYKIPAGSQVVPLINCVHMDPA

LWDEPARFNPARFIDETGKIRRPEFFMPFGVGRRMCLGDVLARMEMFMFFACVMHQFDV

SMEEGAAAPSLEGTVGATIAPEAFRVRFLSRAVPVPAPDHPHLRNVGCH

>PxylCYP4C21

MYFMSHKADDVRTLTNMFVEKPYYYNRFSKMWLGQGLLTGPGHIWKRNVKNLSATFAG

SVIEGFQDIFNRQAAKLTANLKKEIGQPPFDPLHKYFAYATLETICQTALGVTPLSDSIVTPE

YYHAFNRNLQLIVDRAFNIFTHPDFLYRLTPGYREMKKNCAILHNVSRTVIELRRKEYEEK

KKKELTNEPDAIKSKFRPFLDIIMELSDTDPLLTDEQIRNEVDTVILGGQETSATTMLFTLLA

LGSNMDVQEKLYQEIKEIFGDSKRPVQKEDLARLKYCEAVIYESLRLFPPVPAAMRVIETD

TELDGVTIPAGAMFVLNVLGAGRSFAAYGPDARKFRPERWLDGPPINPASFLAFSYGRRAC

IGKRYALTFLKTLLTHIIRDLKIHSCYEDLRLEFSISLRASHGNLITVEARS

>PxylCYP4G15

MSTSPVEEVQGYHYSRLVFYPLLLVASSLWMLYRWQQQSRYFRMGNKLPGPACVPIFGNA

LLLLGKSPDQLVKLGLEYADIYGTVIRGWIGSKLVIFLTDADDVEPILNSQVHIDKATEYRF

FKPWLGEGLLISSGEKWRSHRKMIAPTFHINILKSFMTTFNQNSVNVVDKMKHEMGRTFD

VHDHMSGVTVDILLETAMGITRKTQDESGFDYAMAVMKMCDIIHQRHYKFWLRFDSVFK

FTSFFKKQIELLGIIHGLTNKVIKNKKETYFMNKAKGIIPPTLEELTKSEDNDGTLANDGKT

LADSVFKGYRDDLDFNDENDVGEKKRLAFLDLMIESAQNGTHNISDHEIKEEVDTIMFEG

HDTTAAGSSFVLCLLGVHQDIQRRVYDELYDIFGDSDRPATFEDTLKMKYLERVIFESLRM

YPPVPVIARKLKKDVKIATKGYVLPAGATVVIATFKIHRNGKYYKNPDVFDPDNFLPENTQ

DRHYYSYIPFSAGPRSCVGRKYALLKLKVLLSTILRNYKLISEVPESEFRLQADIILKRTDGF

RVKIEPRRRVPSTAS
